# Educational priorities of low-and middle-income country medical diaspora organisations: A critical discourse analysis

**DOI:** 10.1371/journal.pgph.0003481

**Published:** 2024-07-16

**Authors:** Ishrat Hussain, Lois Haruna-Cooper, Sharon Isiramen, Nina van der Mark, Mishal Khan, Mohammed Ahmed Rashid

**Affiliations:** 1 Faculty of Medical Sciences, University College London, London, United Kingdom; 2 Faculty of Medicine, University of Debrecen, Debrecen, Hungary; 3 Faculty of Public Health and Policy, London School of Hygiene and Tropical Medicine, London, United Kingdom; King's College London, UNITED KINGDOM OF GREAT BRITAIN AND NORTHERN IRELAND

## Abstract

Rising global migration levels have led to growing diaspora populations. There has been interest in the role of diaspora healthcare professionals (HCPs) from low- and middle-income countries (LMICs) in development aid to their origin countries, although there has been comparatively less focus on their educational activities. This study examined the stated educational priorities of LMIC medical diaspora organisations, with a particular focus on the tension between promoting professional opportunities afforded by medical migration and contributing to healthcare workforce shortages due to migration away from LMICs.We gathered a textual archive from webpages and public documents of 89 LMIC medical diaspora organisations in high income countries, predominantly the US and UK. We employed Foucauldian critical discourse analysis to examine presented rationales around educational policies and practices, with a focus on encouragement towards, and discouragement from, medical migration. Two discourses dominated this archive. The first was of *preservation* and framed the educational work of these organisations as a means of providing unity and social networks to diaspora HCPs, with a focus on maintaining their cultural identity and heritage, and medical connections with their origin countries. The second was of *aspiration* and framed their educational work as providing support to diaspora HCPs to advance their careers and maximise training opportunities, often through directly enabling and supporting migration to high income countries. There was a discursive absence around *brain drain* with no policies or practices that overtly sought to deter against, or offset the negative effects of, medical migration. Notwithstanding the valuable contributions that LMIC medical diaspora organisations make in global health, the discursive framings that shape their educational work are linked primarily to protecting and progressing diaspora HCPs rather than on LMIC workforce challenges. Further research is needed to examine potential impacts of these positions on HCP migratory behaviours.

## Introduction

Although international migration is not a new phenomenon in human history [1] global mobility has been steadily increasing since the Second World War under the umbrella of an international ‘liberal’ order [2]. Whilst globalisation has been dominated by the dual pillars of trade and capital flows, migration has been described as the ‘third leg of the stool’ on which the global economy rests [3]. Yet migration is fundamentally different to trade and investment there is an important human consideration as decisions are often taken by individuals and families. Despite the benefits of migration seemingly outweighing the costs overall [4], it raises many important cultural, ideological, and humanitarian dilemmas for policymakers and politicians.

The continual movement of people all over the world had led to the formation of immigrant communities in host countries. Oftentimes, according to a variety of circumstances, these communities actively maintain links with origin countries [5], resulting in a growing recognition of the importance of understanding diaspora. The term ‘diaspora’ was originally applied exclusively to communities living in exile [6], but is now widely used to describe any ethnic minorities building and maintaining communities in hosting countries. Common ethnicity is conceptually important as it lays the foundation of diasporic communities, and what defines them as a group [7]. In this paper, medical diaspora is defined as physicians who have moved from their country of origin to another country.

It has long been established that diasporic communities play an important role in the economic development of their countries of origin, not least through their well-known role as senders of remittances, which are often a significant source of foreign exchange in countries with developing economies [8]. Governments in countries of origin are increasingly recognising their diasporas as sources of knowledge, skills, investment, and commercial connections [9] Moreover, the contributions of migrants and diaspora to sustainable development have also been acknowledged [10] including in global heath [11]. A recent scoping review identified a growing literature examining diasporic engagement with health systems in their origins countries, showing a plethora of different activities ranging from research and training programmes to financial remittances [12].

Medical diaspora groups have received a comparatively high degree of attention, both in the medical sciences [13] and social sciences [14] literatures. This may partly be explained by the perennial debates about the transferability of medical training across countries and contexts [15], as well as the global workforce shortages and inequalities caused by ‘brain drain’ as physicians migrate from the Global South to the Global North for a variety of training and personal reasons [16].

In recent years, this interest has extended from engagement of diaspora in the capacity of individual physicians or small groups acting informally, to a focus on the work of medical diaspora organisations. A 2019 study, for example, formulated an inventory of low- and middle- income (LMIC) medical diaspora organisations in four Global North countries, finding 89 organisations in total [17]. It was beyond the scope of this descriptive study to examine how these organisations position themselves with regard to migration, although the authors concluded that ‘more in-depth research is warranted’ [17].

To date, there has been no critical examination of the role of medical diaspora organisations around migration, with the dominant policy position championing an expansion in their involvement due to their cultural understanding of, and commitment to, their host countries. Inherent to the diaspora position, though, is a historic migratory choice either individually or ancestrally, which potentially represents a particular worldview about global relocation. Notwithstanding deep and sincere diaspora commitments to building capacity and improving quality in their origin country, there is a tension with regard to the framing of migratory choices. This is particularly apparent in the domain of diasporic support in education and training, given that this typically influences physicians who are likely to be contemplating migration for career development.

Recognising this tension, we sought to examine the ways in which LMIC medical diaspora organisations framed their education and training support to origin countries, with a particular focus on their positions on migration through ideas of building capacity and addressing brain drain. Using Foucauldian critical discourse analysis (FCDA), a framework that recognises that discursive notions are constructed at specific times in particular socio-political conditions, we sought to shed light on how the activities of these organisations were prioritised and justified. This approach focuses attention on the way that discourses influence social practices. The research question guiding this study was: *what are the dominant discourses that LMIC medical diaspora organisations use to frame their educational activities for HCPs in origin countries*, *and how do these relate to migration*?

## Methods

Given our interest in educational activities and the positioning of these with regard to migration, which is an inherently social and political issue, we opted to employ FCDA, which enables the interrogation of how some discourses resist or reinforce power and knowledge relations between subjects (e.g., HCPs) and their conceptions of phenomena (e.g., migration). Discourse refers to ways of thinking, writing, and acting, including the limits of what can and cannot be said about a given topic [18]. They therefore define what counts as acceptable criticism [19]. Discourses that emerge as dominant at one particular time often appear to be ‘natural’ or inevitable, allowing the assumptions that support these discourses to go unexamined [20].

Whilst FCDA is underpinned by the work of French philosopher Michel Foucault, he does not offer a single approach to examine discourses, and his work has been taken up in different ways [21]. FCDA explores how language and social structures relate to one another and the exercise of power, analysing properties of language and interactions in their social contexts [22]. We specifically drew on the Foucauldian concept of ‘archaeology’, which involves analysing current assumptions about accepted forms of knowledge to draw attention to how ideas of ‘truth’ are embedded in language [21]. This approach requires one to “unearth as many …discursive elements as possible, assembling them into a developing understanding of the discourse of which they are constituent parts” [21].

The assembly of an appropriate archive is an essential step in FCDA [21]. As previously mentioned, an inventory of LMIC medical diaspora organisations has recently been collated, and this provided the starting point to assemble the textual archive for this study [17]. Although this study used searches of Google, Google Scholar, LexisNexis, Scopus, and PubMed to identify organisations, it was limited by English language searches. Organisations using other languages and those without an online presence will, therefore, not have been captured. Notwithstanding these limitations, the list presented an established repository of organisations of interest for us to interrogate. The organisations identified through this study are characterised by national and ethnic categories (e.g. Persian, Nigerian) rather than generic minority identities (e.g. BAME).

Our research question focused on the educational activities of these organisations, and relevant text about this was typically available on organisational websites, or in documents accessed from these websites. As our archive consisted exclusively of publicly available information on open websites, research ethics approval was not required as per the University College London Research Ethics Committee guidance.

Three broad questions guided our repeated examinations of the texts to identify dominant discourses and their connections (or lack of connections) to migration:

- What are the dominant discourses?
- What are the connections between the dominant discourses and migration?
- What are the relative emphases of the dominant discourses and migration?

The assembled archive was analysed to identify key words and statements that describe educational activities. We looked for direct statements that rationalised particular educational activities or approaches, as well as exploring related ideas and the positioning of statements to identify how prominent they were overall within the document. We also paid particular attention to the contexts and purposes of particular statements and ideas. We then analysed the links between the identified dominant discourses and potential messages about migration, going on to describe the effects and implications of the dominant courses on the outlooks and behaviours of physician learners in countries of origin.

To enhance the dependability of our analyses, we examined the archive through repeated close readings and through communication among the research team to identify prominent statements and recurrent concepts. In each iterative close reading, we analysed the texts for direct statements and principles linked to migration. We also systematically identified and searched for potential proxies of migration and delineated how it was constructed within the texts. We extended our analysis to make sense of the underlying contexts necessary to construct notions of migration from these statements. We analysed the positioning of statements about migration and its proxies in comparison to the dominant discourses to identify relative prominence within the texts.

The research team were mindful of how their own positions, experiences, and personal and professional backgrounds influenced how they approached this work. The team was made up of HCPs at various levels of clinical seniority, as well as non-HCP members from different disciplinary backgrounds, including from global health and medical education. Although all members of the research team are currently based in a HIC setting, some had migrated from LMICs, and others had personal and family backgrounds from LMICs. Throughout their analysis and writing, the research team recognised that their collective personal and professional positionalities and expertise shaped their understanding of the topic area, whilst also acknowledging the many other positionalities not represented.

## Results

Although education was identified as a priority area in the aims and mission statements of virtually all of the LMIC medical diaspora organisations included in this study, we noted discrepancies in the extent to which they offered current (recent or upcoming) educational events. Of the 89 organisations included in this study, 46 (51.7%) had information about current educational events and 27 (30.3%) offered current events specifically focussed on migration. These events typically focused on supporting applications for, and transition following, migration from LMICs to HICs for healthcare training or practice. Example topics are listed in Table 1 and the full data table is included as a supplementary document S1 Data. Some organisations strongly expressed their commitment to education in strong terms and often had broad aims and ambitions, including to ‘support and assist medical education in general’ and to ‘create and maintain a fostering educational environment’. Three organisations had no recent public information published and could be considered inactive.

**Table 1:** Educational topics linked to migration.

U.S. Residency training applications
U.S. Residency match system
U.S. visa regulations and green card applications
Applications to U.S. Educational Commission for Foreign Medical Graduates
Applications to UK General Medical Council
Transitioning and onboarding in U.S. and UK healthcare systems
Interview preparation and ‘mock’ interviews
Job searching advice
CV preparation advice
Personal statement writing advice
Preparing for United States Medical Licensing Examination (USMLE)
English language training for doctors and nurses

The commonest topics covered in educational events related to clinical topics, which covered a wide array of topics at all levels and across all medical and surgical disciplines and specialist areas. Additionally, a number of non-clinical topic areas were covered, including medical ethics, advocacy, gender equity, and leadership. Sessions were most frequently targeted at diaspora HCPs, although some specifically targeted HCPs in origin countries. A significant proportion did not specify a target audience and some events were explicitly open to any attendees by being marketed to the public. A significant majority of educational events were focussed on postgraduate training and continuous professional development, and a smaller proportion targeted undergraduate healthcare students.

The commonest format for educational events was online meetings and webinars, although in person events also featured amongst a wide range of other formats offered, as listed in Table 2. ‘Global health’ featured in the descriptions of events rarely, used to indicate coverage of clinical conditions of global importance rather than referring to mainstream global health topics. Notions of capacity-building and knowledge transfer did not appear.

**Table 2:** Formats of educational events.

Seminar
Webinar
Podcast
Annual scientific meeting
Conference
Symposium
Journal club
Mentorship event
Networking forum
Clinical evidence updates

We identified two dominant discourses in our archive, which together shaped the educational priorities and practices of the included organisations. These discourses were of *preservation* and *aspiration* and each are explored in turn below. In addition, we noted a conspicuous discursive absence around brain drain, with none of the organisations studied either referring to, or hinting at, negative consequences that can arise as a result of migration of HCPs away from LMICs.

The first discourse we identified was of *preservation* and framed the educational priorities of organisations as a means to maintain, and sometimes re-establish, a sense of community of belonging. This included preserving links and connections with the origin country as well as preserving and building links between the diaspora group. As well as the term *preservation* being used directly, related terms including ‘protection, ‘promotion’, and ‘association’ regularly appeared. There was a clear focus on relationships and on the social aspect of educational events, with a strong emphasis on ‘community’ and ‘network’ as goals.

The other important aspect of *preservation* related to a sense of closeness amongst the diaspora group, including ideas of preserving relationships and friendships through ‘unity’ and ‘fraternity’. This was accentuated by many organisations explicitly foregrounding culture in their educational priorities, including descriptions of ‘cultural wealth and heritage’, ‘cultural enrichment’, ‘cultural exchange’, and one organisation purporting to offer ‘a unique blend of educational, cultural and social activities’.

The other dominant discourse we identified in our archive was of *aspiration* and framed the educational priorities of the organisations as a means to advance the professional careers of the diaspora HCPs that they represent. In addition to the term *aspiration* being used directly, related terms frequently featured, including ‘realising potential’, ‘professional excellence’, and ‘career prospects’. The most notable educational topics that were linked to this discourse related to supporting and facilitating medical migration. The largest subset of these topics were directly related to pursuing postgraduate medical training in the United States, with related subjects being covered including detailed information on visas, professional examinations (specifically the United States Medical Licensing exam), observerships and placements, certification with the Educational Commission for Foreign Medical Graduates, residency selection advice, writing personal statements, mock interviews and application guidance, and integrating to the U.S. healthcare system after arriving. A number of organisations focussed specifically on catering for those who ‘desire to train in the US’ and providing them with a comprehensive ‘road map’ on how to achieve this. Although the main focus was on migration to the U.S., a smaller subset of organisations based in the UK offered equivalent educational events related to migration to the UK, again with a focus on this as an aspirational and progressive pathway.

The *aspiration* discourse was not exclusively focused on supporting migration and was also used to justify courses that focussed on advancing the careers of diaspora HCPs. Organisations sought to offer educational opportunities that would improve their knowledge and skills, help them access better employment opportunities, and achieve leadership positions in the healthcare system. Although the focus on aspiration was predominantly about achieving excellence, some organisations recognised the challenges faced by diaspora HCPs and thus framed their educational activities around ameliorating this and maximising the chances of success in spite of these hurdles.

In addition to the two dominant discourses, we also identified a noteworthy discursive absence related to *brain drain* in the archive. Given the centrality of migration to HICs to the diaspora experience, it was unsurprising that it was a focal topic in this archive. However, whilst the *aspiration* discourse provided the backdrop for educational activities that supported further migration, there was no counter-discourse that focussed on negative or unintended consequences of migration such as brain drain, particularly on LMICs. Although some organisations expressed allegiances to their origin countries and a smaller number identified interests in their educational priorities, this was focussed on knowledge, skills, equipment, and research in LMICs. This could be conceptualised as *brain gain* as opposed to *brain drain*, although it nonetheless did not form a dominant discourse and the predominant focus on these events was on the *preservation* discourse earlier described. Only a single organisation in the entire archive acknowledged shortages of HCPs in their origin country and there was overall no discursive formation around providing educational solutions that would ameliorate or manage this.

## Discussion

The use and meaning of diaspora has expanded in recent years with greater interest in global economic and cultural flows and the crossing of related borders. Dufoix [23] describes an ‘unbelievable proliferation of studies focused on the theme of connections—of all sorts—that are established, preserved, or undone beyond borders.’ Brubaker[24] outlines three areas critical to the continued validity of diaspora as a concept, which define the tensions central to its evolution: dispersion, homeland orientation and boundary-maintenance. That these conditions are met in the LMIC medical diaspora organisations analysed in this study suggests that the findings of this study can reliably be situated in the broader academic study of diaspora.

The concept of ‘homemaking’, which has been used synonymously with the term ‘diasporic nostalgia’, refers to ethnic groups organising their belonging in a newly adopted homeland by keeping connected with their parental or ancestral society [25], and is widely acknowledged in the field of diaspora studies. As Sengupta [26] notes, preservation of culture is an essential element of this process for ethnic minority groups in a predominantly white milieu. The discourse of *preservation*, linked to both identity and tradition, was dominant in the medical diaspora organisation archive in this study and has been widely recognised as a key aspect of the diaspora experience all around the world [27–30].

Aspiration is the second dominant discourse identified in this study and is likewise a well-established component of diaspora scholarship. Appadurai [31] conceptualises aspiration as a cultural capacity, constructed ‘in the thick of social life’ and embedded with family-mediated values that shape people’s imaginations for the future. This understanding draws attention to the reality that some have more opportunities to exercise their capacity to explore pathways to their imagined futures—in part because of the unequal distribution of social and cultural capital [32–34] links this directly to the diaspora experience. Drawing on the work of Bhabha [35] and others, Kardaszewicz [36] similarly emphasises the importance of understanding a ‘good education’ as part of the diaspora identity, expressed as ‘the daily engagement with the local environment and in the actual experience of pursuing educational aspirations’. Like preservation then, aspiration is a recognised feature of the diaspora experience.

Given that there has been a recent trend for policymakers to advocate for a greater role for medical diaspora organisations to improve healthcare outcomes in LMIC origin countries, this study has important implications for where and how this support may best be targeted. Given that HCP workforce shortages are a perennial challenge facing all healthcare systems, and especially LMICs, the educational scope of medical diaspora organisations should be crafted in ways that are cognisant that their educational interventions are discursively positioned in a way that is broadly supportive of migration. Policymakers in HICs who are focussed on managing differential attainment and limited opportunities for international medical graduates, meanwhile, may use the findings of this study to consider how the aspirational discursive framings of medical diaspora organisations can inform future supportive interventions. Practitioners working in global health and LMICs, including those in the organisations covered in this study and others, may use the findings of this study to reflect on how their language and ideas may present particular positions and perspectives related to migration to HICs.

This is the first study to critically examine how LMIC medical diaspora organisations frame their educational priorities and how this related to migration choices of HCPs. The finding that cultural preservation and aspiration are important in the educational offering to these HCPs warrants further investigation, particularly in light of the need for solutions on how migrant HCPs can best be supported to integrate and thrive in HIC settings. In particular, the role of community and belonging could be explored in further research to understand the extent to which existing educational interventions for this group provide this. Further research is also required to understand how the organisational discourses uncovered in this study do (or do not) align with the perspectives of individual HCPs who work for them. Explicitly examining the perspectives of these organisations and HCPs around migration would also be helpful to understand the extent to which the discourses identified here are deliberate or otherwise. Finally, further research is needed to understand how HCPs in LMICs conceptualise these organisations and their educational interventions, and whether or not particular educational offerings change their perspectives about future migration choices.

The strengths of this study lie in the examination of a diverse set of LMIC medical diaspora organisations using a methodological and theoretical approach that helps to uncover unsaid and previously unseen patterns that relate to power and knowledge. In particular, this approach has helped to uncover a noteworthy discursive absence that has potential implications for how these organisations can and should operate. The major limitation is that, as previously described, the list of organisations is limited by language and online parameters. Moreover, it relies exclusively on document analysis and therefore requires further empirical research, as described above, to help contextualise and enrich the findings.

## Conclusion

Considering our increasingly globalised healthcare landscape, diaspora HCPs are an important group to understand both in terms of individual healthcare systems, as well as the global medical community. Although not all diaspora HCPs are members of medical diaspora organisations, these organisations are nonetheless becoming recognised as an important component of support for LMICs. This study has highlighted that the education provided by these organisations is primarily focussed on enhancing the lived experience of diaspora HCPs, focussing particularly on helping them to preserve ties with their origin countries as they aspire to excel and achieve success in their new HIC positions. The primary focus of their educational interventions is supportive of migration to HICs, with little evidence of discouraging, or identifying negative consequences of, such migratory choices. Further research is needed to examine the implications of these findings in terms of the wider context about educational support and migration. In the meantime, there is a need for policymakers to consider how these organisations can best be positioned to support HCPs, including diaspora HICs and their counterparts in LMICs.

## Supporting information

S1 Data (PDF)
