# Supplementary material for: Educational priorities of low-and middle-income country medical diaspora organisations: A critical discourse analysis
Source: PLOS Glob Public Health. 2024 Jul 16;4(7):e0003481. doi: 10.1371/journal.pgph.0003481 (PMC11251574; doi:10.1371/journal.pgph.0003481)
Supplement: S1 Data — (PDF) [file pgph.0003481.s001.pdf]

Medical diaspora organisations	URL Link	Aims of the organisation	Medical Education for healthcare professionals – Undergraduate/Pre-Clinical	Medical Education for healthcare professionals – Postgraduate/Clinical	Migration related education
Academy of Persian American Physicians	https://www.facebook.com/acedemyofpersianamericanphysicians/	The Academy of Persian Physicians was established in 1988 with unified effort and cooperation of a group of accomplished and well-known Iranian physicians and surgeons. The most important objectives of the Academy are: 1. Conservation of the Iranian cultural wealth and heritage. 2. Preservation and promotion of the ethical, scientific and practical values of the medical profession. 3. Assistance to social programs for the enhancement of the Iranian quality of life. The members of the academy are dedicated to their commitments and responsibilities and are determined to persistently improve their quality of medical practice and valuable ethical issues. It is the policy of the Academy not to be involved in any political or ideological activities. Therefore to be able to concentrate on the professional, cultural and social programs.	NA	NA	NA
Afghan Medical Association of America	http://www.afghanmed.org/	The Afghan Medical Association of America is dedicated to improving the current health status of the Afghan nation and has been actively involved in extending medical and educational aid to Afghanistan. This association is not a part of any group or political party and is concerned with the basic human rights of the people of Afghanistan.<br>The AMAA is an independent association and not influenced by any government or groups inside or outside Afghanistan.	NA	NA	NA
Afghan Medical Professionals Association of America	https://www.ampaa.org/	The Afghan Medical Professionals Association of America (AMPAA) is a charitable 501(c)3 organization dedicated to uniting Afghan healthcare professionals, providing medical education, and providing medical assistance and care for all people within the United States and Afghanistan. Our mission is humanitarian and without a political agenda.<br>Since mid August 2021, AMPAA has been working alongside US government partners, state departments of health, Centers for Disease Control and Prevention, and private sector. AMPAA is the go-to organization in supporting US government processes and resettlement efforts for Afghan refugees as it relates to medical care in a culturally competent manner.	NA	23rd May 2023 - Health and Wellness of Afghan Newcomers seminar<br><br>December 2022 - Cultural Competency Training for Medical Providers<br>- We offer print material, modules, virtual sessions, webinars, and in-person training.<br>- In response to the health issues of Afghan resettlement process as a result of the US withdrawal from Afghanistan, AMPAA has produced a number of healthcare-related videos in cooperation with IMANA. These videos are presented in Pashto and Dari to educate new arrivals and provide our community with important information about the health issues	24th September 2022 - 31st annual scientific meeting<br>- The focus of the meeting was on new Afghan arrivals with medical background and their ability to re-enter the medical field. The event also provided explanations regarding the Pathway to Graduate Medical Education for international medical graduates in the United States, the United States Medical Licensing Examination (USMLE), Educational Commission for Foreign Medical Graduates (ECFMG), Certification, and Residency.<br>- The event also included two panel discussions focusing on opportunities and careers in allied health, public health, radiology, and pharmacies, and the medical disciplines.
Albanian American Medical Society, Inc.	https://www.albamedsociety.org/	1. To hold educational seminars in the Albanian populated territories of the Balkan Peninsula (Albania, Kosovo, Macedonia, Montenegro) thereby to offer on a charitable basis advanced training and medical education to the Albanian health care professionals, through scheduled lectures, discussions, publications and other means.<br><br>2. To create and maintain a fostering educational environment between the public academic institutions in the Albanian populated territories and those here in the US wherein its members may meet to exchange medical knowledge and participate in continuing medical education.<br><br>3. To create and maintain an educational exchange program for Albanian healthcare professionals to come to the United States so they may learn from the US healthcare system and in turn improve the healthcare system in their home countries. To assist students and other healthcare professionals who desire to train in the US by mentoring them and providing educational opportunities through observerships in the US healthcare system.<br><br>4. To provide charitable healthcare services to the underserved communities in both the United States and in the Albanian territories of the Balkan Peninsula through general medical checkups, consultations and education of patients on disease prevention through medical missionary work.	7th March 2022 - This evening we will be having a webinar in partnership with the Albanian American Medical Student Society of Loyola University Chicago! The webinar will be an open ended format where current medical students can answer questions and give perspective to medical students looking to enter their fourth year and preparing for residency.<br>12th April 2021 – Med Student Panel	Journal Club<br>- 19th April 2023 – Predicting the safety and effectiveness of inferior vena cava filters (PRESERVE) trial: Outcomes at 12 months<br>- 16th March 2023 - Prevalence and Risk Factors for Pharmacoresistance in Children With Focal Cortical Dysplasia–Related Epilepsy<br>- 9th February 2023 - Once-Weekly Semaglutide in Adults with Overweight and Obesity<br>- 16th November 2022 - External Ventricular Drainage: "A nationwide prospective multicenter study of external ventricular drainage: accuracy, safety, and related complications<br>- 20th July 2022 - Surviving Sepsis Guidelines<br>- 23rd May 2022 - Ten-Year Outcomes of Off-Pump vs On-Pump Coronary Artery Bypass Grafting in the Department of Veterans Affairs.<br>- 21st April 2022 - Diagnosis and Management of Inflammatory Bowel Disease<br>- 9th March 2022 - Thrombectomy Complications in Large Vessel Occlusions: Incidence, Predictors, and Clinical Impact in the ETIS Registry<br>- 6th February 2022 - Xenotransplantation: How close are we to a human clinical trial?<br>- 19th January 2022 - Cavity Progression and Prevention<br>- 28th October 2021 - Breast Cancer: Screening, Diagnosis, and Treatment<br>- 25th August 2021 - Dermatology Emergencies: Severe Cutaneous Adverse Reactions to Drugs	27th May 2023 – Applying to residency information panel<br>- This event brings together panelists from both US and international medical schools to provide guidance and share their knowledge with medical students who are applying to residency programs in the United States. Our panelists, who have successfully matched into US residency programs, will offer valuable insight into the application process, requirements, and what programs are looking for in candidates. Attendees can expect to learn about the differences between applying as a US medical student versus an international medical graduate, as well as tips and strategies for crafting a strong personal statement, selecting the right programs to apply to, and excelling in interviews. This event is especially beneficial for IMGs, who may face unique obstacles and require additional support during the application process<br>17th October 2022 - Albanian American Medical Student Society of Loyola University Chicago Collaborative Speaker Series<br>7th March 2022 -

		5. To plan a variety of public events through which these stated goals can be materialized	- The webinar will be an open ended format where current medical students can answer questions to prospective medical students about the application process and medical school in general.<br>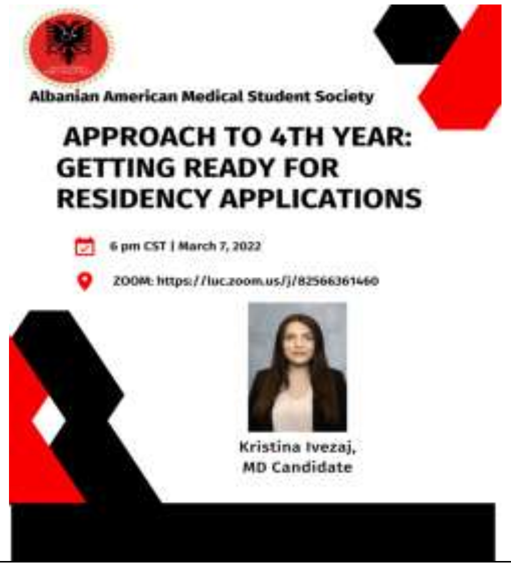 	- 12th July 2021 - Gastrointestinal symptoms associated with COVID-19: impact on the gut microbiome<br>- 16th June 2021 - Surgical Outcomes in Acute Mesenteric Ischemia: Has Anything Changed Over the Years?<br>- 2nd June 2021 - Pathologic Antibodies to Platelet Factor 4 after ChAdOx1 nCoV-19 Vaccination<br>- 17th May 2021 - Persistent neurologic symptoms and cognitive dysfunction in non-hospitalized Covid-19 "long haulers<br>- 20 April 2021 - the management of heart failure and Ermelinda Ndoka (MS-1) will review the article "Dapagliflozin in Patients with Heart Failure and Reduced Ejection Fraction<br>- 17th March 2021 - Prevention and Treatment of Colorectal Cancer<br>- 9th February 2021 - A Randomized Trial Comparing Antibiotics with Appendectomy for Appendicitis	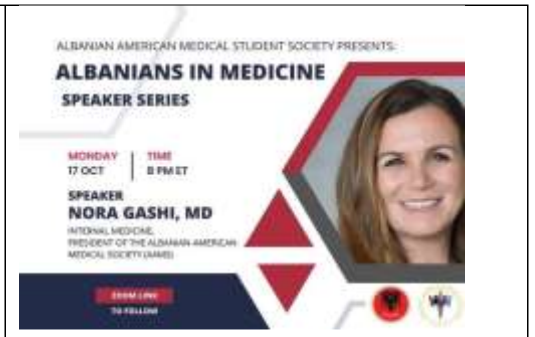<br><br>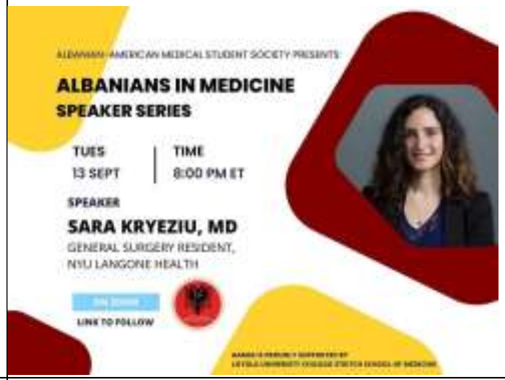
American Association of Cardiologists of Indian Origin	http://www.aacio.org/	1. Provide a central forum for physicians and scientists of Indian origin in the U.S.A. who have interest in cardiovascular diseases. This will promote close relationship amongst members for mutual benefit and advancement. We will work with organized medicine at local, state, and national levels and project the interest of our members.	NA	5th March 2023 – In person cardiology educational meeting, presentations on dyslipidaemia, amyloidosis<br><br>6th November 2022 – Virtual cardiology educational meeting, presentations on treating heart failure in CKD, out of hospital cardiac arrests, ECHO in ECMO<br><br>3rd April 2022 – Virtual cardiology educational meeting, presentations on ECHO in assessing HF, bleeding mitigation on antiplatelet therapies, managing stable CAD	NA
		2. Promote and maintain high standard of academic excellence and clinical practice of cardiology through educational, social, and scientific activities. This will be in the common interest of its members and the communities they serve.		14th November 2021 – Virtual cardiology educational meeting, presentations on personalizing medicine in cardiovascular medicine, heart failure	
		3. Maintain close liaison with local, national, and international cardiovascular societies and organizations.		22nd May 2021 – Virtual cardiology educational meeting, presentations on dual-antiplatelet therapy, 2D/3D ECHO adult congenital heart disease, cardiac wellness	
		4. Establish a continuing relationship and communication with similar societies in India.			
		5. To recognize and honor outstanding cardiovascular professionals of Indian origin in various disciplines.			
		6.Address the special cardiovascular health problems of the Indian community in the US.			
American Association of Physicians of Indian Origin	https://aapiusa.org	Founded in 1982, the American Association of Physicians of Indian Origin represents the values and interests of more than 80,000 practicing physicians in the United States.<br>In addition, it also serves as a platform for more than 40,000 medical students, residents, and fellows of Indian origin in this country. The AAPI YPS/MSRF (Young Physicians Section/Medical Students, Residents and Fellows Section) is a vital and integral part of AAPI and their participation and contributions in the AAPI leadership and field of medicine is highly valued. 1in 7 people in	NA	Webinars<br>- 20th May 2023 – Physician burnout<br>- 30th April 2023 – Obesity and lifestyle medicine<br>- 29th April 2023 – Kidney failure<br>- 18th March 2023 - Physician Compliance with the Stark Law, Anti-Kickback Statute and Professional Liability Cases<br>- 12th February 2023 – Congenital heart defects<br>- 3rd December 2022 - Ultra-fast detection of all Human Cancers using non-invasive liquid biopsy	Webinars<br>- 8th April 2023 – New EB-5 law benefits and comparison with EB-2 for foreign medical doctors of Indian origin<br>- 25th April 2021 – Preparing an application for Match 2022<br><br>30th November 2021 - Virtual Career Fair

		the USA, at some point in their health care, are touched by a physician of Indian origin and we are truly honored for this privilege and responsibility.<br>AAPI also has a Charitable Foundation (AAPI-CF) that supports free clinics and health fairs in India. To date, more than 7 such clinics are active in various states of India under the AAPI partnership.		- 19th November 2022 - Lifestyle Medicine & Wellness<br>- 1st October 2022 - South Asian Heart Disease:<br>- Current Concepts in Better Prediction, Detection and Prevention of Heart Attack in South Asians<br>- 28th September 2022 - US COVID-19: Who is At High Risk?<br>- 10th September 2022 - Treating Depression in Children and Adolescents in Primary care<br>- 20th August 2022 - Monkeypox outbreak, What Physicians need TO know & Covid Pandemic 2022 brief update<br>- 2nd June 2021 - Clinical Spectrum of Fibrosing Interstitial Lung Disease<br>- 14th May 2022 – CME on hospice and palliative care<br>- 13th November 2021 – Telemedicine perspectives, keeping up with the changes in healthcare<br>- 29th September 2021 - Duchenne Muscular Dystrophy<br>- 15th September 2021 – Public health, advancing health equity and operationalizing racial injustice<br>- 8th September 2021 – Precision medicine for mental health: How to save and improve lives<br>- 11th August 2021 – Update on COVDI-19 variants<br>- 7th August 2021 - Advocacy as a Critical Component of Patient Care: Why Physician Advocacy Matters and How to Succeed At It<br>- 2nd May 2021 – Outpatient treatment of COVID 19<br>- 21st April 2021 - Peripartum Anxiety and Depression: A Silent Epidemic, Public Health Urgency, and Global Impact<br>- 4th March 2021 – Women heart and health<br>- 24th February 2021 - The rising burden of kidney disease in India – challenges, and opportunities<br>- 30th January 2021 - Challenges in Healthcare in the COVID Era<br>- 23rd January 2021 - Mini Liver Symposium<br>- 13 January 2021 - Resuscitation Updates: 2020 AHA Resuscitation Guidelines & Cardiac Arrest Registries<br>- 5th January 2021 - Workplace Wellness – BMI Day AAPI Seven Pillars of Lifestyle Magic NObesity Revolution – Wear Yellow<br><br>6th-8th January 2023 - AAPI Global Health Summit<br>- Psychiatry, community mental health<br>- Maternal and infant nutrition in the development of NCDs in India<br>- Cardiology<br>- Neurology<br>- Blindness prevention<br>- Diabetes and kidney disease<br>- Oncology<br>- Integrative medicine<br><br>17th-19th February 2023 – Winter medical conference<br>- Achieving Excellence in Gender Equity - Improving the Female Physician Experience<br>- MedTech<br>-<br>23-26th June 2022 – Convention & Scientific Assembly – Continuing medical education<br>- Healthcare equity and ethics<br>- Managing chronic disease<br>- Digital/contemporary medicine<br>- Breakthroughs in medicine<br><br>5-7th January 2022 – Global healthcare summit<br><br>18-20th March 2022 – Winter Medical Conference<br><br>19-21st February 2021 – Winter medical conference	Connect with hundreds of physicians not in the Practice-Match database across the nation that are looking to pract in the US. **Over 60% of all attendees will be US citizens.**
American Board Certified Doctors for Egypt	http://www.doctorsforegypt.com/	The organization brings together Egyptian physicians who are volunteering to enhance the academic, clinical, quality improvement and research aspects in Egypt.	NA	12 February 2023 – Haem-onc case discussions<br><br>19th January 2023 – Breast cancer talk<br><br>23rd October 2022 – Haem-onc case discussions	March -April 2023 – Post-match interviews, Various Egyptian doctors describing their journeys to Match in a US residency<br><br>22nd March 2023 – Preparing for the match, Visa J1 exchange visitor program

				28th August 2022 - Breast cancer talk<br><br>24th July 2022 - Haem-onc case discussions<br><br>12th June 2022 – GI oncology talk<br><br>31st March 2022 – Journal club – Diffuse large cell lymphoma<br><br>25-27th February 2022 – International neonatal multispeciality conference<br><br>21 November 2021 – Breast oncology case discussions<br>28th September 2021 - Basic Approach to review article and meta-analysis<br>25th September 2021 - Basics of medical research for IMG<br><br>8th April 2021 – Lecture - Status of Female Genital Mutilation/Cutting in the Egyptian immigrant population in the US.<br><br>4th April 2021 - Overview of Neuroendocrine Tumors<br><br>1st April 2021 – Neonatal multi-speciality NeoVirtual webinar<br><br>28th March 2021 – Paediatric tumour board<br><br>27th March 2021 - Wellbeing, Technology, Immigration, Diversity, program-specific related tracks (Internal Medicine/ Psychiatry/ Surgery/ Podiatric),<br><br>20th March 2021 - Updates on management of preterm labour<br><br>13th March 2021 – Breast cancer tumour board<br><br>7th March 2021 – Haematologic malignancies and BMT board<br><br>7th February 2021 – Oncologic emergencies	15th May 2022 - Checkout this new podcast by Dr Mohamed Saleh, which explores the lives and career tracks of Egyptian physicians who travel abroad for various careers after graduation, offering unique perspectives that are seldom explored, including a discussion on US Visa and green card, challenges related to research involvement during medical school, the physician-scientist career pathway, and other psychosocial issues.<br><br>16th October 2021 – SOAP notes - We will discuss the SOAP notes format and how important to dictate professional, concise, and precise patient note<br><br>23rd July 2021 – Interns a month inn: issues and concerns - Dr. Mohamed Hegazi, the ABCDE president invites the new PGY1 residents to an interactive session via clubhouse to discuss the challenges encountered by the new residents in the US healthcare system. We would like to hear new residents' feedback.<br><br>2nd May 2021 - We are delighted to announce for the upcoming webinar "Alternative pathways; Way to USA" in collaboration with the "American Board Certified Doctors for Egypt" for the third successive years.<br><br>27-28th March 2021 – Virtual residency summit booth - Happy to announce I will be joining amazing panel of medical educators at the First FREE Online Virtual Onboarding Summit for ALL rising interns of #Match2021 hosted by Buddies Space:<br>The Summit will be hosted (March 27-28, 2021) on Hopin with 30+ Speakers, panellist, and workshops by leading experts in medical training who will cover 4 main themes regarding staring residency this year including: #wellbeing , #innovation , #immigration, and #diversity, program-specific related tracks, and more…We'll also showcase several tools and resources that will be available for the residents who are staring their training July, 2021.<br>All Medical #trainees and #educators are most welcomed<br>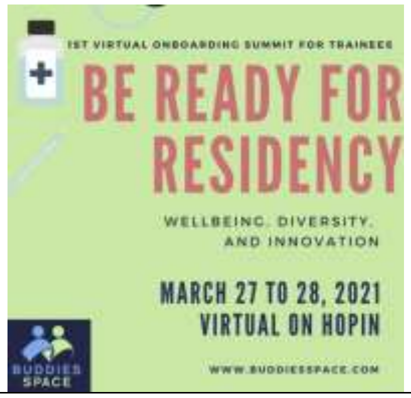
American Lebanese Medical Association	http://www.almamater.org/page/mobile-clinic-2023	ALMA aims to develop a society through which physicians and other health care professionals who share Lebanese heritage or a humanitarian interest in Lebanon and it's people may come together and undertake concrete projects to improve the health and well-being of all of Lebanon's diverse religious and cultural communities.<br><br>ALMA Fosters the creation and development of academic institutions and health care facilities of excellence in Medicine and Surgery in Lebanon. We want to build a Lebanese / American Doctor's Library, that provides access to advanced publications, books, magazines and audio-visual material to Lebanese medical and health care professionals.	NA	14-15th July 2023 - Lebanese Order of Physician's International Congress<br>This congress is an opportunity to focus insightful on the different challenges facing our collapsed healthcare system and how we can benefit from the expertise of the Lebanese Medical Diaspora to rise again by implementing the right "Vision" and "Reform" to build a balanced medical map and a new healthcare infrastructure providing a unified standard of care to all our people. In addition, this congress will highlight the achievements of Lebanese Doctors in different specialties in Lebanon and worldwide under the session "Meet the Experts".	NA

		We plan to assist, through publication, subscription and wide dissemination, Lebanese scientific publications to reach a wider audience and publish high quality scientific material. ALMA Sponsors annual medical convention in Lebanon to enhance advanced training and promote scientific exchanges between American and Lebanese medical professionals.			
America Nepal Medical Foundation	https://www.anmf.org	America Nepal Medical Foundation (ANMF) was established in 1997 by a group of Nepalese doctors, originally from Nepal, and American doctors who loved Nepal. Dr Arjun Karki was the founding president.	NA	26-28th May 2023 - 1st Americas Regional Health Conference - This conference is expected to bring together health professional like physicians, nurses, public health experts, allied health workers, health advocates to provide an integrated holistic approach to health. Any disease or it's risk factor is best addressed through an integrated approach of prevention, curative and management including public health, nursing and physician services. This conference will discuss ways how these disciplines could work together for the optimal delivery of health. This conference is highlighting health issues of people of Nepali origin in the Americas regions and exploring ways of addressing them through partnership and collaboration. Health concern of Nepal will also be discussed with equal emphasis.<br>5-7th May AGM 2023 –<br>-Burn Care ICU need in Nepal<br>-Filling gaps in child healthcare delivery in Nepal<br>-Challenges of starting Liver Transplant in Nepal<br>-and many more topics including networking and collaboration in healthcare delivery<br><br>18th June 2022 - Preoperative Critical Events in Obstetrics training program to be held on 6/18/22 This training program is organized by University of Louisville, Kentucky in collaboration with Paropakar Maternity and women hospital along with NAMS, Department of Anesthesia. It will include both didactic session followed by hands on exercise using simulators.<br><br>14th January 2022 – Update on COVID-19 – Preparing for the Omicron surge<br><br>28th October 2021 - Update on COVID-19 – Virus strains and vaccines<br><br>18th September 2021 - Digitalization of healthcare sector in Nepal panel discussion<br><br>4th September 2021 – Medical research in Nepal<br><br>25th August 2021 – Preventing progression of COVID-19 prior to ICU admission<br><br>21st August 2021, 27th July 2021, 25th June 2021 – Ventilator training sessions in Nepal<br><br>13th June 2021 – COVID-19 related respiratory failure – Oxygen therapy and beyond<br><br>26th May 2021 - ANMF and Alliance COVID 19 Relief Efforts: Update<br><br>15th May 2021 – COVID-19 treatment in Nepal – Remdesivir and convalescent plasma use<br><br>25th April 2021 – COVID-19 in Nepal: New strains and vaccines<br><br>24th April 2021 – COVID-19 case discussion: What's new in Nepal's second wave<br><br>19th April 2021- Tumour board<br><br>22nd march 2021 – oxygen therapy and Covid19<br><br>13th march 2021 – covid19 vaccination roll-out in Nepal<br><br>6th February 2021 – covid-19 vaccines and new strains	28th August 2022<br>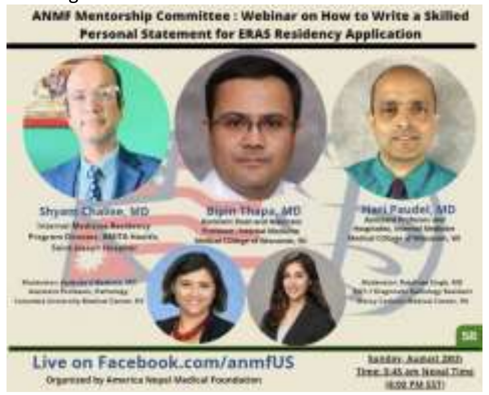<br><br>28th August 2021<br>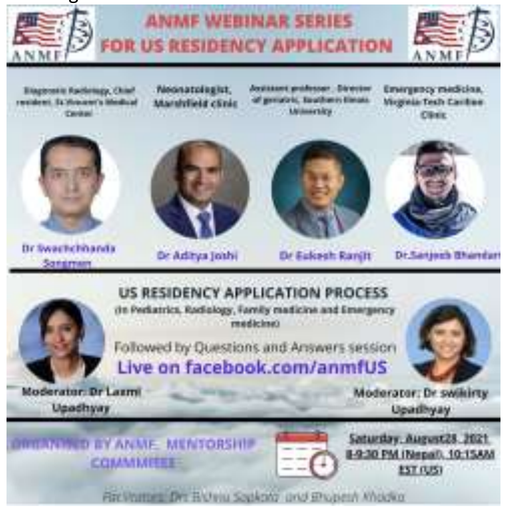<br><br>27th February 2021<br>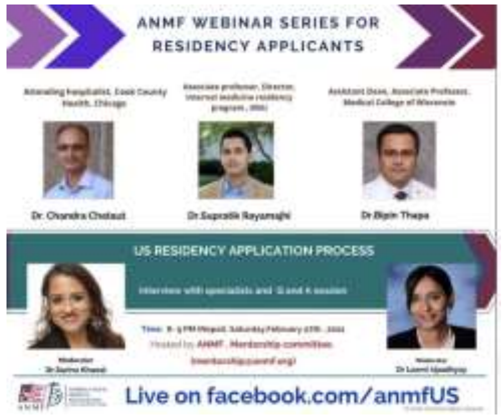
American Society of Indian Plastic Surgeons	https://www.facebook.com/American-Society-of-Indian-Plastic-Surgeons-189293321104812/	NA	NA	NA	NA
American Ukrainian	http://aumf.net	Our mission is to lend support to Ukraine in strengthening its healthcare and bringing it to the highest international standards by	NA	25th February - 25th April 2022 – Webinar series about emergency orthopaedics	NA

Medical Foundation		developing a platform for cooperation and mutual enrichment between Ukraine and the United States.			
Argentine-American Medical Society	http://www.aams.us	The Argentine-American Medical Society welcomes you to join us in representing more than 3000 Argentine physicians, doctors graduated in Argentine universities and medical schools and other professionals who are living and working in the United States of America.<br><br>The AAMS was founded in 1981 with the aim of connecting those professionals practicing across the country.<br><br>Since the beginning, we have been organizing social and scientific events with the purpose of promoting and giving us the opportunity of sharing our work in the field of the medical practice and research. We have been maintaining links with other professional associations with the purpose of enhancing our representation as a growing group of foreign medical graduates.	NA	NA	NA
Armenian American Medical Society	https://aamsc.org	The Armenian American Medical Society is the world's largest medical organization for medical professionals of Armenian descent. For more than 35 years, we have provided vital resources to health practitioners and patients, as well as humanitarian assistance and outreach around the world.	NA	28th June 2023 - Child and Adolescent Mental Health: Common Problems and Recent Trends - "Your Health" Community Education Program interview<br>28th June 2023 – Innovative laprascopic colon cancer surgery talk, Armenia during the pandemic, healthcare during the Artsakh conflict<br>24th May 2023 – What is the future of stroke webinar<br>20th May 2023 - 1st Annual Healthcare wellness symposium<br>10th May 2023 – Structural and valvular heart disease and procedures interview<br>12th April 2023 – Hereditary cancer syndromes in the Armenia population interview<br>22nd March 2023 – cochlear implant program interview<br>8th March 2023 – Diabetic foot care interview<br>28th February 2023 – Infection control webinar<br>February 2023 – Can a broken heart cause a heart attack presentation<br>11th January 2023 – Intermittent fasting interview<br>14th December 2022 – bladder cancer review interview<br>23rd November 2022 – Update on mens health interview<br>29th September 2022 – Common neurosurgical conditions – Intracranial haemorrhage and benign brain tumours interview<br>11th September 2022 - 18th Annual CME and CDE Viva Las Vegas Getaway Weekend - The day offered an amazing slate of presentations by dedicated AAMS members. Topics ran the gamut of relevant health care subject matter, including cardiology, cancer screening, pediatric Covid, the use of robotics in dental implant surgery and more. One of the most talked about —and most timely — topics of the day was implicit bias, empathy and the practitioner.<br>22nd June 2022 – New updates on heart failure guidelines interview<br>26th May 2022 - Infant Nutrition and the Infant Formula Shortage interview<br>21st May 2022 – Spring symposium – updates in medicine<br>27th April 2022 - Introduction to Rheumatology and Different Types of Arthritis interview<br>13th April 2022 - Natural Remedies for Seasonal Allergies interview<br>23rd March 2022 – A brief review of colon cancer (screening and treatment) interview<br>9th March 2022 - Effects of Genetic Factors and HLA on the Severity of COVID-19 in the Armenian Population interview<br>26th February 2022 - Neurosurgical Case Conference online<br>24th February 2022 – Paediatric covid update interview<br>9th February 2022 - Latest Update on COVID interview<br>7th February 2022 - Infection Control Update: It's a New Era webinar<br>26th January 2022 - Depression and Anxiety in the Era of the Pandemic interview<br>8th December 2021 - The Omicron Variant and Vaccine Boosters interview<br>6-7th November 2021 - 13th Armenian Medical World Congress, Setting the Stage for Synergy and Collaboration to Ensure a Healthy and Prosperous Nation<br><br>13th October 2021 - Breast Cancer Management Overview interview<br><br>29th July 2021 - Vaccine Hesitancy interview<br><br>14th July 2021 – Healthcare and the LGBTQ community interview<br><br>19th June 2021 - Symposium on Cardiovascular Diseases and Therapies	18th March 2023<br>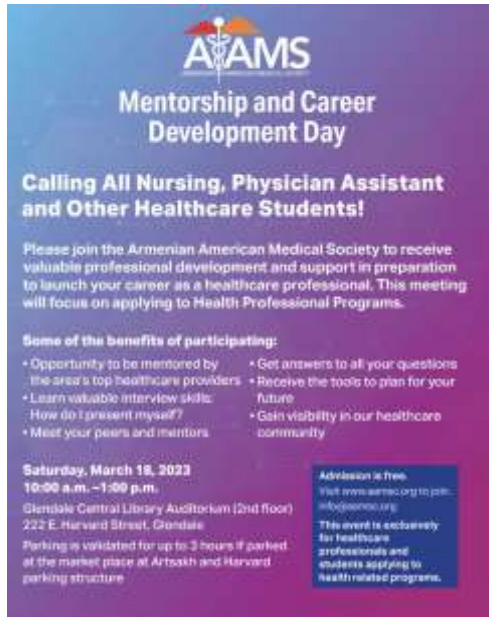<br><br><br>11th March 2023<br>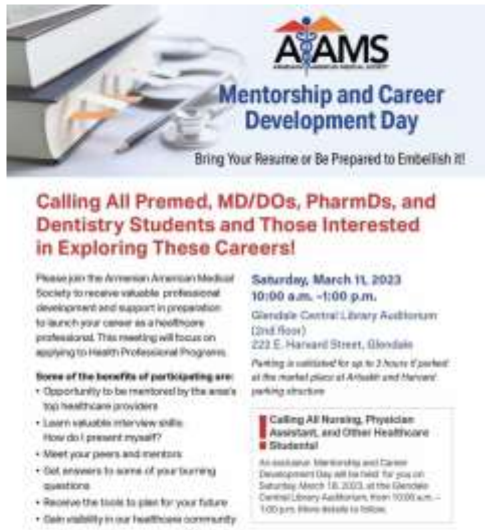<br><br>19th December 2021

				18th May 2021 - Oxygen: Breath of Life interview<br>12th May 2021 - COVID-19 and Oxygenation Updates from Armenia and Beyond interview<br>19th April 2021 - Temporomandibular Dysfunction And Facial Pain presentation<br>24th March 2021 - The Latest on COVID Vaccines in the USA interview<br>10th March 2021 - Hereditary Breast Cancer in the Armenian Population interview<br>24th February 2021 - What We Know About Covid Vaccines interview<br>8th February 2021 - The latest on the Covid Vaccine interview	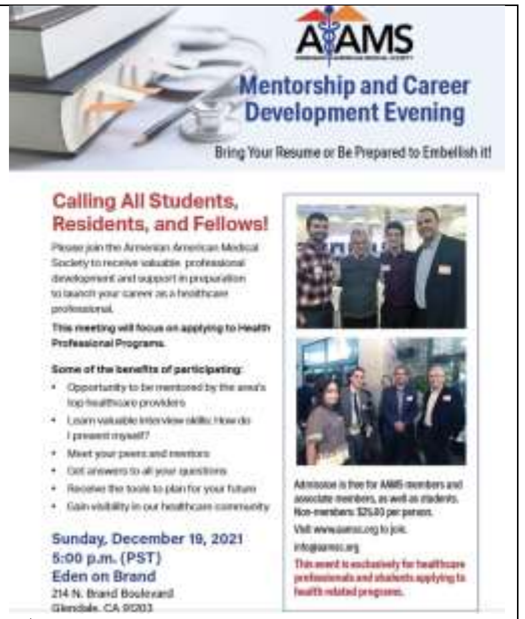<br>3rd November 2021<br>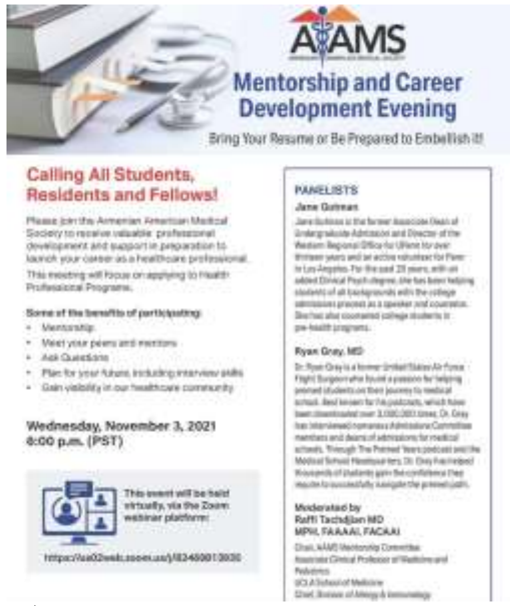<br>14th April 2021<br>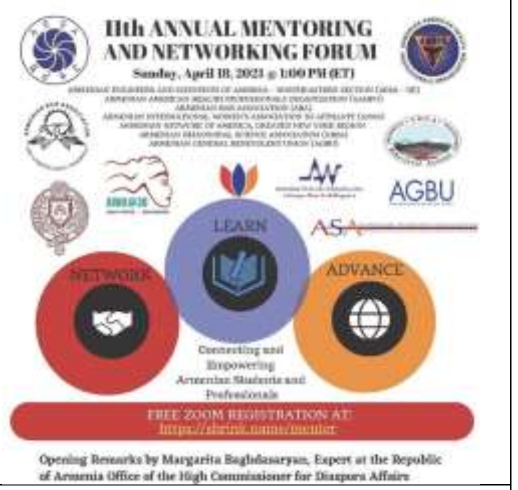
Armenian Medical Society of Great Britain	http://www.accc.org.uk	The AMA-GB brings together Armenians in Great Britain who work in the health sector or who support the aims of the organisation. We exist to promote health initiatives for Armenians in Great	NA	30th March 2021 – Scientific spring meeting (online) – long covid syndrome and corona virus vaccination dilemma	NA

		Britain and the Republic of Armenia and to support professionals and volunteers working in health care, whether here or abroad.			
Association of Afghan Healthcare Professionals UK	http://aahpuk.org	We are a group of Afghan healthcare professionals in the UK, and came together to set up this organisation in 2011 to improve the life of Afghan people. Since then, we have been devoted to use our knowledge and expertise to improve the public health system in Afghanistan and to address some of the health issues of the Afghan community both in Afghanistan and in the UK.	NA	NA	NA
Association of Chinese American Physicians	http://www.acaponline.org/	The Association of Chinese American Physicians (ACAP) is a non profit professional organization of Chinese American physicians and physicians that care for the Chinese community. One of ACAP's objectives is to promote quality healthcare through physician networking and professional development.	NA	20th April 2022 - virtual workshop on symptoms, treatment and management of Hepatitis B<br>22nd March 2022 - webinar discussing Colorectal Cancer screening, treatment, and prevention.<br>19th March 2022 - webinar discussing the pros and cons of cardiac stents and how to prevent coronary artery disease.<br>26th January 2022 - cervical health and cancer / screening webinar<br>3rd October 2021 - ACAP 26th Annual Convention<br>- Breast Cancer Screening in the Era of COVID-19<br>- Medical, Public Health and Social Challenges of COVID-19<br>- Our annual convention will feature an outstanding CME program including updates on technology, treatment and diagnosis of infectious diseases that afflict our patient population. Our supporters will include a wide range of community hospitals, pharmacies, pharmaceutical companies, medical device companies, and healthcare partners.<br>20th July 2021 – Chest pain webinar<br>15th June 2021 – Alzheimer's webinar<br>15th April 2021 – Heart disease webinar	25-26th March 2022 - two day virtual seminar where you can hear from recent Chinese medical graduates share their match experience, success stories, and advice!<br>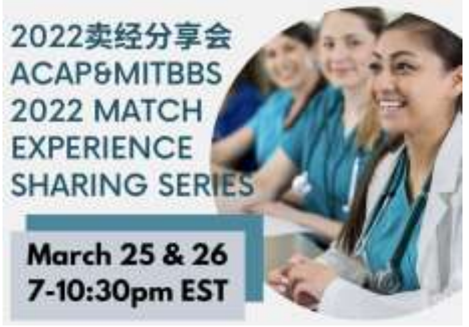<br>27th March 2022 - NEED ADVICE ON MEDICAL CAREER PLANNING?<br>Come and hear our speakers share and discuss their advice and personal experiences about Residency, Fellowship, Application tips, Interview Skills, Job Search; as well as topics related to running your own Medical Practice.<br>14th March 2021 - ACAP Career Development Seminar starts in 3 days - this Sunday March 14 at 10am. Come and hear our speakers sharing their advice and personal experiences about Residency, Fellowship, Interview Skills, Job Search; as well as topics related to running your own Medical Practice.<br>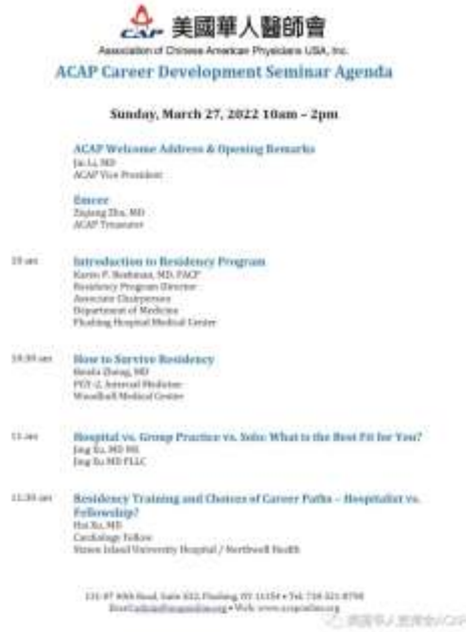<br>美國華人醫師會<br>Association of Chinese American Physicians USA, Inc.<br>ACAP Career Development Seminar Agenda<br>Sunday, March 27, 2022 10am – 2pm<br>ACAP Welcome Address & Opening Remarks<br>Emcee<br>Introduction to Residency Program<br>How to Survive Residency<br>Hospital vs. Group Practice vs. Solo: What is the Best Fit for You?<br>Residency Training and Choices of Career Paths – Hospitalist vs. Fellowship?

					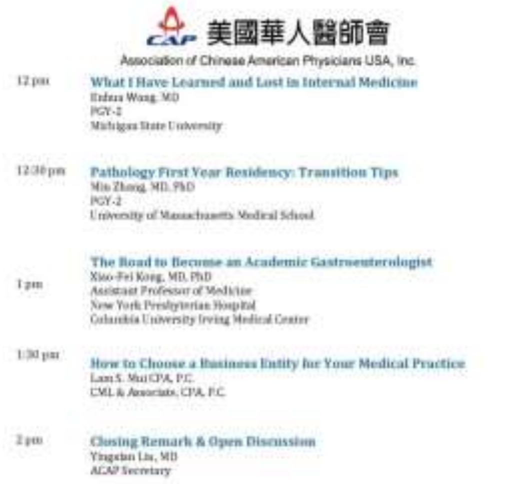<br>美國華人醫師會<br>Association of Chinese American Physicians USA, Inc.<br>12 pm What I Have Learned and Lost in Internal Medicine<br>Erdan Wang, MD<br>PGY-2<br>Michigan State University<br>12:30 pm Pathology First Year Residency: Transition Tips<br>Min Zhang, MD, PhD<br>PGY-2<br>University of Massachusetts Medical School<br>1 pm The Road to Become an Academic Gastroenterologist<br>Xiao-Fei Kong, MD, PhD<br>Assistant Professor of Medicine<br>New York Presbyterian Hospital<br>Columbia University Irving Medical Center<br>1:30 pm How to Choose a Business Entity for Your Medical Practice<br>Lam S. Mui CPA, P.C.<br>CML & Associate, CPA, P.C.<br>2 pm Closing Remark & Open Discussion<br>Yingxian Liu, MD<br>ACAP Secretary<br><br>5th November 2021 –<br>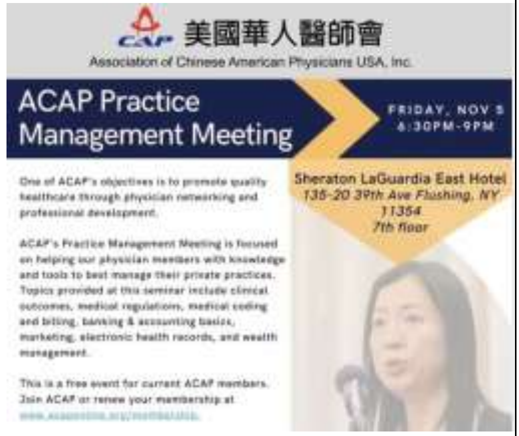<br>2nd August 2021 - ACAP's Mock Interview is a popular annual program that helps candidates apply for Residency and Fellowship programs. Over 20 physicians from local clinic and hospitals will interview applicants and help them to improve interview skills.

Association of Haitian Physicians Abroad	http://www.amhe.org/index.html	The Association of Haitian Physicians Abroad (AMHE), was founded in August 1972, by a group of Haitian physicians determined to mark their presence as a growing ethnic entity in America, to foster professional alliances, and to promote the health and interest of the Haitian immigrant community at large.	27th February 2022 – Teaching session for 4th year medical student in Haiti	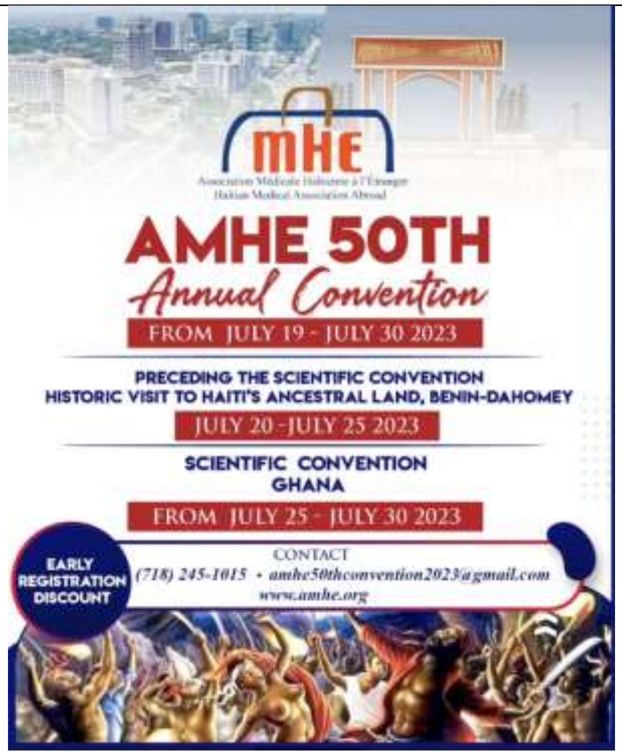<br>8th August 2023 - Notre scientific program at the AMHE Convention was centered on: "Medicine at a crossroad of two continents: America and Africa<br><br>12th May 2021 – Survival and recurrent TB in people with HIV and a history of one episode of TB – Online event<br><br>4TH May 2022 – Online orthopaedic webinar on Legg-Calve-Perthes disease<br><br>30th April 2022 - – Online orthopaedic webinar on shoulder arthroplasties	Mentorship Programme since Nov 29th 2021<br>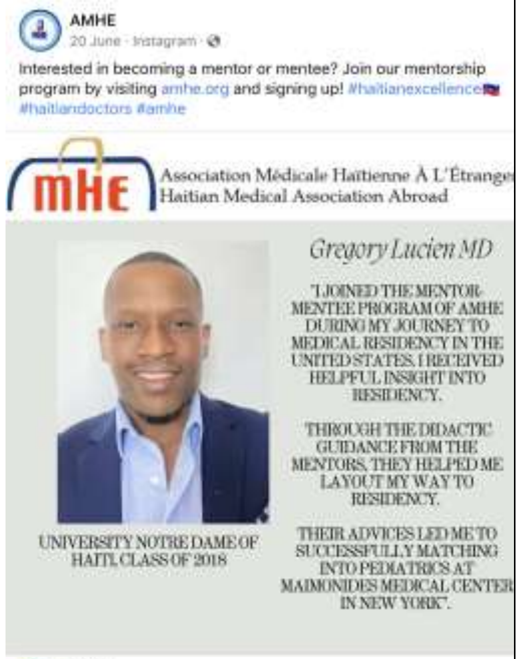<br>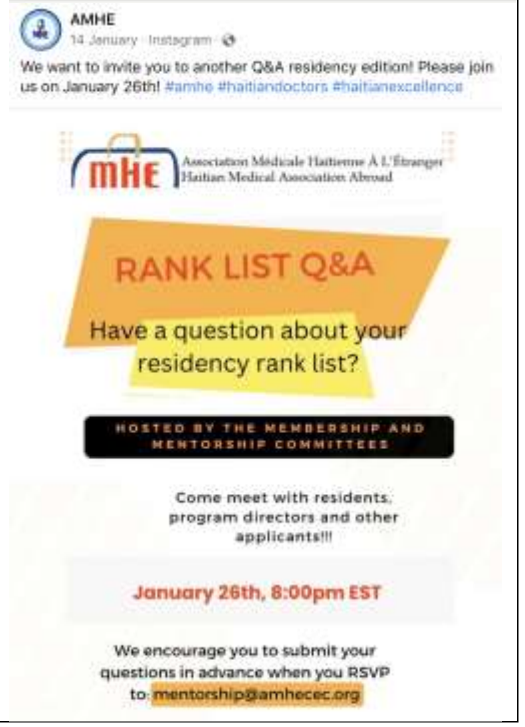

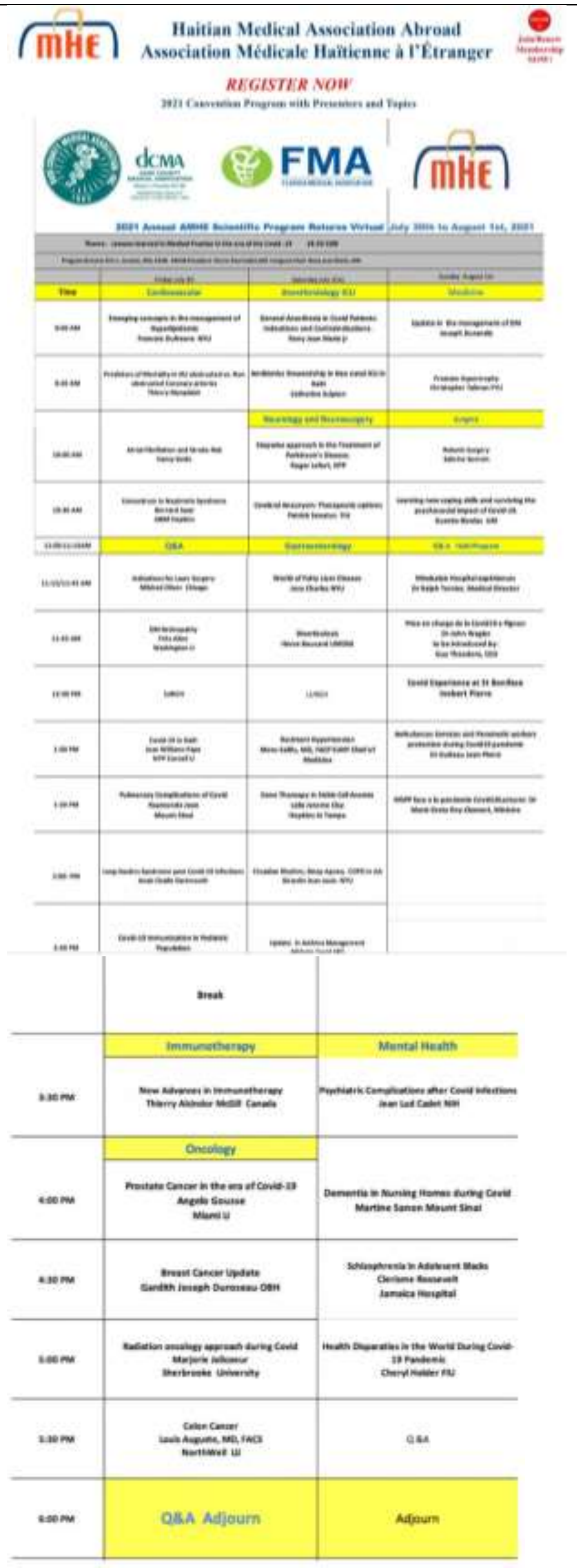

	Break	
	Immunotherapy	Mental Health
3:30 PM	New Advances in Immunotherapy<br>Thierry Alcindor McGill Canada	Psychiatric Complications after Covid Infections<br>Jean Lud Cadet NIH
	Oncology	
4:00 PM	Prostate Cancer in the era of Covid-19<br>Angelo Gousse<br>Miami U	Dementia in Nursing Homes during Covid<br>Martine Sanon Mount Sinai
4:30 PM	Breast Cancer Update<br>Gardith Joseph Duroseau OBH	Schizophrenia in Adolescent Blacks<br>Clerisme Roosevelt<br>Jamaica Hospital
5:00 PM	Radiation oncology approach during Covid<br>Marjorie Jolicoeur<br>Sherbrooke University	Health Disparities in the World During Covid-19 Pandemic<br>Cheryl Holder FIU
5:30 PM	Colon Cancer<br>Louis Auguste, MD, FACS<br>NorthWell LIJ	Q&A
6:00 PM	Q&A Adjourn	Adjourn

5th March 2022 – Cardiology online symposium

14th March 2021 – Pulmonary diseases online course

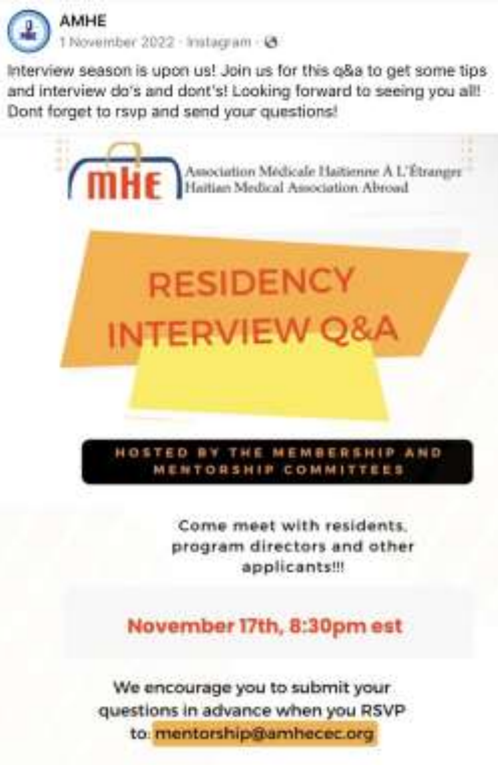

				6th March 2021 – Cardiology symposium onine	
Association of Kerala Medical Graduates	http://www.akmg.org/	AKMG {Association of Kerala Medical Graduates} has been the premier and pioneer Alumni Organization in North America {USA and Canada} for Medical and Dental specialists of Kerala origin, since its inception in 1979-80, thereby setting the precedent for an organization of healthcare professionals of any ethnic or international origin in North America.	NA	CME 2023 agenda (below)	Rising star mentorship programme – 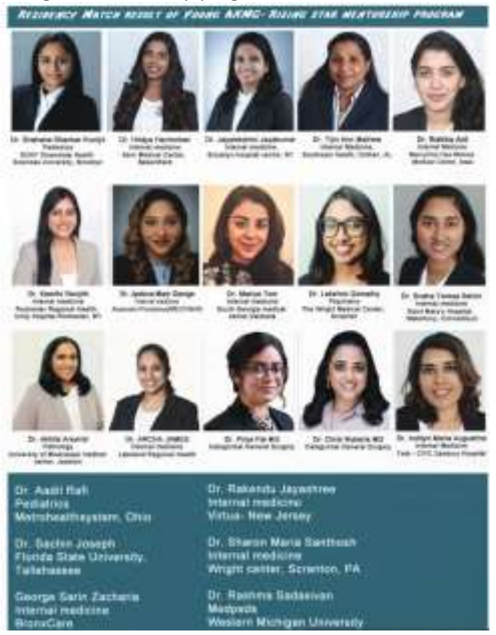

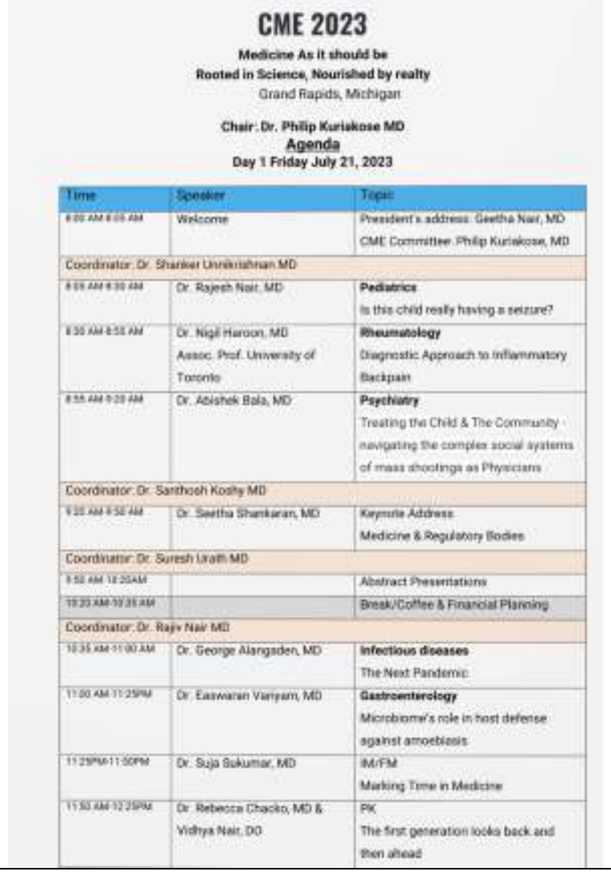

CME 2023

Medicine As it should be
Rooted in Science, Nourished by realty
Grand Rapids, Michigan

Chair: Dr. Philip Kuriakose MD
Agenda
Day 1 Friday July 21, 2023

Time	Speaker	Topic
8:00 AM-8:05 AM	Welcome	President's address: Geetha Nair, MD<br>CME Committee: Philip Kuriakose, MD
Coordinator: Dr. Shanker Unnikrishnan MD		
8:05 AM-8:30 AM	Dr. Rajesh Nair, MD	**Pediatrics**<br>Is this child really having a seizure?
8:30 AM-8:55 AM	Dr. Nigil Haroon, MD<br>Assoc. Prof. University of Toronto	**Rheumatology**<br>Diagnostic Approach to Inflammatory Backpain
8:55 AM-9:20 AM	Dr. Abishek Bala, MD	**Psychiatry**<br>Treating the Child & The Community - navigating the complex social systems of mass shootings as Physicians
Coordinator: Dr. Santhosh Koshy MD		
9:25 AM-9:50 AM	Dr. Seetha Shankaran, MD	Keynote Address<br>Medicine & Regulatory Bodies
Coordinator: Dr. Suresh Urath MD		
9:50 AM-10:20AM		Abstract Presentations
10:20 AM-10:35 AM		Break/Coffee & Financial Planning
Coordinator: Dr. Rajiv Nair MD		
10:35 AM-11:00 AM	Dr. George Alangaden, MD	**Infectious diseases**<br>The Next Pandemic
11:00 AM-11:25PM	Dr. Easwaran Variyam, MD	**Gastroenterology**<br>Microbiome's role in host defense against amoebiasis
11:25PM-11:50PM	Dr. Suja Sukumar, MD	IM/FM<br>Marking Time in Medicine
11:50 AM-12:25PM	Dr. Rebecca Chacko, MD & Vidhya Nair, DO	PK<br>The first generation looks back and then ahead

CME 2023

Medicine As it should be
Rooted in Science, Nourished by realty
Grand Rapids, Michigan

Chair: Dr. Philip Kuriakose MD
Agenda
Day 2 Saturday July 22, 2022

Time	Speaker	Topic
Coordinator: Dr. Santhosh Koshy MD		
8:00 AM-8:25 AM	Dr. Sangeetha Kodoth, MD	Allergic diseases - a migrants burden ?
8:25 AM-8:50 AM	Dr. Ajit Tharakan, MD	Cardiothoracic Surgery<br>Advances in Cardiothoracic Surgery:
8:50 AM-9:15 AM	Dr. Madhu Menon, MD	Hematopathology<br>Future of Medicine from genomics to chat gpt & everything in between
Coordinator: Dr. Philip Kuriakose MD		
9:15 AM-9:45 AM	Dr. Emil Kuriakose, MD	KEYNOTE ADDRESS<br>Medicine & Industry
Coordinator: Dr. Suresh Urath MD		
9:45 AM-10:15 AM		ABSTRACT PRESENTATIONS
10:15 AM-10:30 AM	Break/Coffee & Financial Planning	
Coordinator: Dr. Khaleel Ashraf MD		
10:30 AM-10:55 AM	Dr. Shyam Prasad Aravindaksha, BDS	Dentistry<br>The interface of Dentistry & Medicine
10:55 AM-11:20 PM	Dr. Maya Raiman, MD	Dermatology<br>Oh Gosh, that's a rash:
11:20 AM-11:45 AM	Dr. Girish Nair, MD	Pulmonary Medicine<br>ILD-practical implications:
Coordinator: Dr. Rajeev Nair MD		
11:45 AM -12:20 PM	Dr. Khurshid Ghani, MD &<br>Dr. Biju Poulose, MD	AKMG Speaks: All Burnt Out<br>Saving one's Professional & Personal Life
12:20 PM-12:25 PM	Dr. Santhosh Koshy MD	Vote of Thanks

5-6th August 2022 – AKMG CME 2022 – Chronic diseases and comorbidities

Day 1

Friday August 5, 2022

Time	Speaker	Topic
**Obesity and Metabolic syndrome**<br>Chair: Susan George Bahl, CM, MD, FRCPC, FACP		
0800-0830	Sreekumaran Nair, MD, PhD, Professor, Mayo Clinic, Rochester MN	Take Diabetes and Pre-diabetes seriously
0830-0850	Ambika Ashraf, MD<br>Professor, Univ. of Alabama	Pediatric Dyslipidemia – Do We need to be concerned?
0850-0910	Pappachan Joseph, FRCP, Lancashire Teaching Hospital NHS Foundation Trust, UK	Medical management of Obesity
0910-0950	*Trainee abstract presentations*	Chairs: Drs. Suresh Urath and Khaleel Ashraf
0950-1000	*Break*	
**Advances in Cardiovascular Medicine: A 2022 Update**<br>Chairs: Krishnakumar Nair, MD, DM and Rakesh Gopinathan Nair, MD, MA		
1000-1030	Salim Yusuf, OC, MD, BS, D. Phil, FRCP, FRCPC, FACC, Professor, McMaster University	Could 1 pill reduce your risk of heart attacks?
1030-1045	Jooby John, MD, MPH, Cardiovascular Associates, Orlando, FL	No more cracking the chest: Advances in Coronary and Valvular Interventions
1045-1100	Nisha Pillai, MD, FACP, FACC<br>Asst. Prof. Northwell Health	COVID and the heart: Inflammation, Arrhythmias and long COVID
1100-1120	Rakesh Gopinathan Nair, MD, MA, Professor, University of Missouri-Columbia	Atrial Fibrillation: Shifting treatment landscape in 2022
1120-1140	Krishnakumar Nair, MD, DM<br>Assoc. Prof.<br>University of Toronto	Device therapy in Heart Failure and structural heart disease
1140-1155	Hafiza Khan, MD,<br>Baylor Scott & White<br>The Heart Hospital, Plano, TX	Wearable consumer technology: what a practicing clinician needs to know<br>Bio hacking or Bio nuisance?
1155-1215	Enas A. Enas, MD<br>Downers Grove, IL	Escalating burden Premature Coronary Artery Disease (CAD) in Keralites and Indians: How to prevent and manage?
1215	*Lunch and break for day*	

<table>
<tr><td></td><td></td><td></td><td></td><td>
Day 2
Saturday, August 6, 2022
<table>
<tr><th>Time</th><th>Speaker</th><th>Topic</th></tr>
<tr><td colspan="3">Infection, Immunity and cancer
Chairs: Drs. Jiana Paul, MBBS and Vimoj Nair, MBBS MD FRCPC</td></tr>
<tr><td>0800-0830</td><td>George Abraham, MD, MPH, FACP
President of ACP</td><td>COVID-19: Lessons from the Pandemic for Public Health</td></tr>
<tr><td>0830-0850</td><td>Vinod Chandran, MBBS, MD, DM, PhD, FRCPC, Assoc. Prof. Univ of Toronto</td><td>Traditional treatments and small molecules for treating immune-mediated rheumatic diseases</td></tr>
<tr><td>0850-0910</td><td>Nigil Haroon, MD, DM, PhD, MBA, FRCPC Assoc. Prof. Univ of Toronto</td><td>Advanced therapies for Immune-mediated rheumatic diseases</td></tr>
<tr><td>0910-0925</td><td>Rakesh Mohankumar, MBBS, MRCS, FRCR, FRCPC, Asst. Prof. Univ of Toronto</td><td>Imaging in Musculoskeletal pathologies. Choosing Wisely.</td></tr>
<tr><td>0925-0945</td><td>Nickie Mathew MD, MSc, ABPN, FRCPC, ABPM
Medical director: Complex mental health and substance use services, BC</td><td>Overcoming the opioid crisis</td></tr>
<tr><td>0945-1000</td><td>Renju Kuriakose, MBS, MD, DM
Head of Neurology,
Dalhousie and Memorial University</td><td>The New Lazarus Effect in Parkinson's Disease</td></tr>
<tr><td>1000-1030</td><td>Soumya Swaminathan, MD
Chief Scientist, WHO</td><td>Keynote Lecture: Equity in healthcare & lessons from COVID19</td></tr>
<tr><td>1030-1045</td><td colspan="2">Break</td></tr>
<tr><td colspan="3">Chairs: Drs. Susan V. George MD, MACP, SFHM, FRCP and Khaleel Ashraf MD, MBBS, DMRD, FACP</td></tr>
<tr><td>1045-1130</td><td>Trainee abstract presentations</td><td>Chairs: Drs. Madhu Bhasker and Syam Kumar MD</td></tr>
<tr><td>1130-1145</td><td>Jame Abraham, MD, FACP
Professor, Cleveland Clinic</td><td>Breast Cancer: What do we need to know in 2022</td></tr>
<tr><td>1145-1200</td><td>Oormila Kovilam, MD, FACOG, MFM
Maternal Fetal Medicine, CHI Health
Creighton University Medical Centre</td><td>Aspects of Gynecological Cancers in Reproductive Age</td></tr>
<tr><td>1200-1215</td><td>Paul Mathew, MD, DNBPAS, FAAN, FAHS
Assistant Professor of Neurology,
Harvard Med School</td><td>Headache: It's All In Your Head…or Is It?</td></tr>
<tr><td>1215-1230</td><td>Suresh Nayar, MDS, FDS (Rest Dent) RCS, MPhil, President, British Society of Prosthodontics</td><td>Let's get digital! - in Maxillofacial Prosthodontics</td></tr>
<tr><td>1230</td><td colspan="2">Lunch and end of CME</td></tr>
</table>
14th August 2021 - AKMG Atlanta CME 2021
<table>
<tr><th>TIME</th><th>SPEAKER</th><th>MODERATOR</th></tr>
<tr><td>7:30 am - 7:35 am</td><td>Dr. Subra Bhat / Dr. Khaleel Ashraf</td><td></td></tr>
<tr><td>7:35 am - 7:50 am</td><td>Dr. Dhanu Meleth
Vitreoretinal surgeon, Uveitis specialist, Marietta Eye Clinic
"Systemic Implications of Retinal Diseases"</td><td>Dr. Lizy Thaliath</td></tr>
<tr><td>7:55 am - 8:10 am</td><td>Dr. P. Gopalakrishan
Attending Surgeon, Medical University of South Carolina (MUSC), Florence-Marion Medical Center
"Health benefits and science behind Kerala rituals"</td><td>Dr. Lizy Thaliath</td></tr>
<tr><td>8:15 am - 8:30 am</td><td>Dr. Nisha Nigil
Assistant Professor, Internal Medicine and Endocrinology
"Managing diabetes in 2025"</td><td>Dr. Lizy Thaliath</td></tr>
<tr><td>8:45 am - 9:00 am</td><td>Dr. Venkit Iyer
Consultant Surgeon, Helen Ellis Memorial Hospital, Tarpon Springs, FL
"Prevention of Medical errors"</td><td>Dr. Nisha, K.S</td></tr>
<tr><td>9:05 am - 9:20 am</td><td>Dr. Shailaja Nair
Director, Jefferson Integrated Practice in Women's Health
"Women's Health: Reality and Misconceptions"</td><td>Dr. Nisha, K.S</td></tr>
<tr><td>9:25 am - 9:40 am</td><td>Dr. Neena Thomas-Eapen
Associate Professor of Family and Community Medicine
"Integrative primary care perspectives on a healthy diet"</td><td>Dr. Nisha, K.S</td></tr>
<tr><td>9:45 am - 10:00 am</td><td>Dr. Enas P Enas
President & CEO CADI Research Foundation
"Malignant heart disease in Indians at a young age: Unravelling the enigma"</td><td>Dr. Suresh Urath</td></tr>
<tr><td>10:10 am - 10:25 am</td><td>Dr. Joji Varghese
Interventional Cardiologist, Cardiology Consultants, Abilene, TX
"Vascular disease and Impact of Statins"</td><td>Dr. Sheraj Jacob</td></tr>
<tr><td>10:30 am - 10:45 am</td><td>Dr. Nigil Haroon
Co-Director, Spondylitis Program, University Health Network
"Approach to back pain and Treatment advances in Spondylo-Arthropathies"</td><td>Dr. Sheraj Jacob</td></tr>
<tr><td>10:55 am - 11:00 am</td><td>Poster Session</td><td>Dr. Rojy Jacob
Dr. Suresh Urath</td></tr>
</table>
</td><td></td></tr>
<tr><td>Association of Nepali Physicians in America</td><td>http://anpa-usa.org</td><td>Focussed on the objectives of providing health education and health information to indigent Nepali community in the US by holding periodic health fair and organising other health related activity.</td><td>NA</td><td>NA</td><td>NA</td></tr>
<tr><td>Association of Nigerian Physicians in the Americas</td><td>https://anpa.org</td><td>THE ASSOCIATION OF NIGERIAN PHYSICIANS IN THE AMERICAS (ANPA), was incorporated in 1995 for educational, scientific and charitable purposes as a tax-exempt non-profit organization under Section 501(c) (3) of the Internal Revenue Code.

ANPA represents the professional interests of 4,000 plus physicians, dentists and allied health professionals of Nigerian birth, ethnicity or empathy in the United States, Canada and the Caribbean providing a platform for medical and scientific dialogue,</td><td>NA</td><td>9th June – Current - Closing the gap podcast – Interviews with various health care professionals

21st – 24th June 2023 – 28th Annual Convention – Innovation for effective and equitable healthcare in the 21st century

June 2023 – Project ECHO – Hypertension management</td><td>15th October 2022 – Managing a medical practice in Africa from the USA – Webinar

28th August 2022 – Residency application overview: 'the nuts and bolts'</td></tr>
</table>

		on issues of health within North America, the Caribbean, and Africa, pertinent to persons of descent from the Nigerian Diaspora.		8th April 2023 – The scourge of cardiovascular disease in the black population: Focus on hypertension the silent killer – Webinar<br><br>10th October 2022 – Ramification of the long COVID-19 syndrome on patients' and the healthcare system – Webinar<br><br>1st October 2022 – Chronic kidney disease education – Webinar<br><br>22-26th June 2022 – health care delivery during crisis and conflict<br><br>18th June 2022 – Impact of COVID-19 on health care delivery system – Webinar<br><br>27th May 2022 – current trends in the management of atrial fibrillation – Webinar<br><br>25th March 2022 – current trends in the management of end stage renal disease including renal transplantation – Webinar<br><br>23rd -26th June - 2022 Scientific convention and assembly<br>- Stressors on healthcare workers during COVID-19<br>- Opioid use disorders<br><br>4th December 2021 – Hepatitis B impact on patient and public health – Webinar<br><br>26th November 2021 – Emergency medicine in a resource limited setting – Webinar<br><br>20th November 2021 – accountability in healthcare delivery – Webinar<br><br>29th October 2021 - Current trends in the management of acute kidney injury in the hospitalised patient including dialysis - webinar<br><br>15th October 2021 - Culture of care in healthcare delivery – Webinar<br><br>24th September 2021 - Current trends in the management of aortic stenosis including transcatheter aortic valve replacement webinar<br><br>7-11th July 2021 - Emergency care and emerging infectious diseases in Nigeria annual convention<br><br>26th June 2021 - Improving patient safety by preventing medication errors webinar<br><br>10th March 2021 - Mental health during COVID-19 webinar<br><br>20th February 2021 - Monoclonal antibodies and other point of care therapies in COVID-19 webinar<br><br>7th February 2021 - Pulmonary and ICU management of COVID-19 in Nigeria webinar<br><br>6th February 2021, 16th January 2021 - COVID-19 vaccine conference online	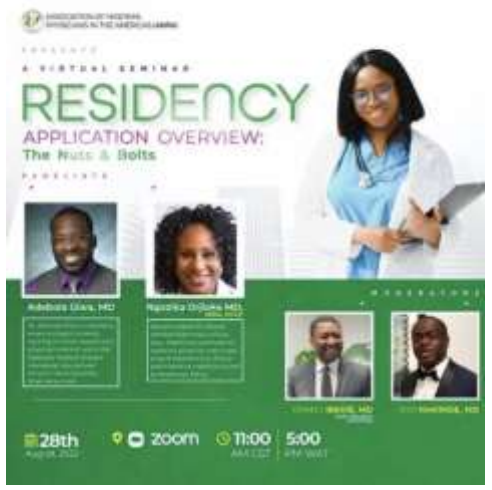<br>28th May 2021 - Dispelling myths about residency in the US webinar<br>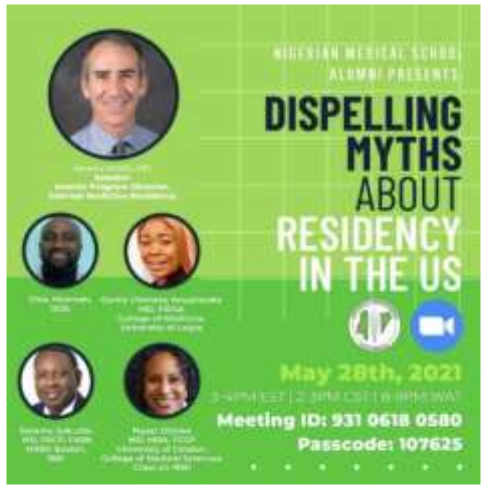<br>22nd May 2021 - Onboarding new residents symposium<br>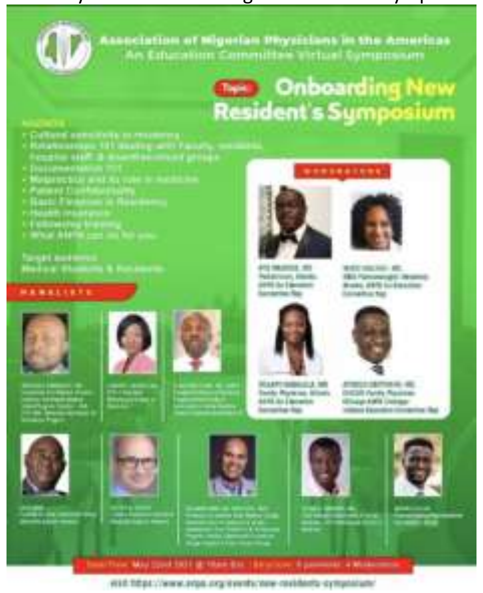<br>26th February 2021 - Residency ranking order and post-match scramble<br><br>30th January 2021 – Journey into medicine and general surgery in the USA – panel talk Instagram live

Association of Pakistani Physicians and Surgeons of the UK	http://appsuk.org	The APPS is an independent, educational and not for profit organisation with no political motives. It is a professional association that provides a platform for Pakistani-British doctors, dentists & allied health professionals in the United Kingdom as well as those coming from Pakistan.<br><br>It aims to protect and promote the interests of its members and raise awareness of health issues among the community. The association will use all its resources through the support of its members and in partnership with other sister organisations, to Modernise the Health System of Pakistan.<br><br>It strongly believes in a lifelong learning process among healthcare professionals and would organise short-term courses, workshops and seminars for medics & the general public. Doctors from all branches of medicine, dentists, allied health professionals and students in medical or allied fields are encouraged to join!		19th March 2023 – Ramadan and health<br><br>5th March 2023 - An APPS Europe meeting in Barcelona with the representatives of Catalonia Spanish Government to discuss a multicentre research project in Europe & UK about screening and treating communities for Hepatitis B & C.<br><br>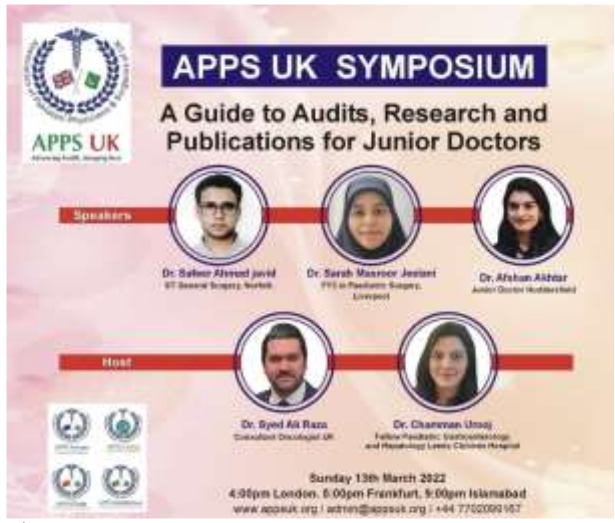<br>6th February 2022 – Psychological impact of screen and games addiction webinar<br><br>31st October 2021 – Pakistan health vision 2030<br><br>29th September 2021 - Why fully vaccinated population, getting worse of COVID webinar<br><br>3rd May 2021 - Impending COVID-19 crisis in Pakistan. What should we do? Webinar<br><br>4th April 2021 - Artificial Intelligence for Orthopaedic Injuries Management via Telemedicine Software – webinar<br><br>25th February 2021 - Using telemedicine to fight COVID-19 in Pakistan webinar	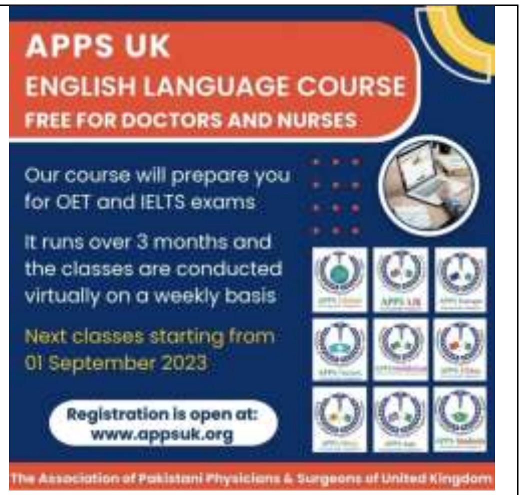<br>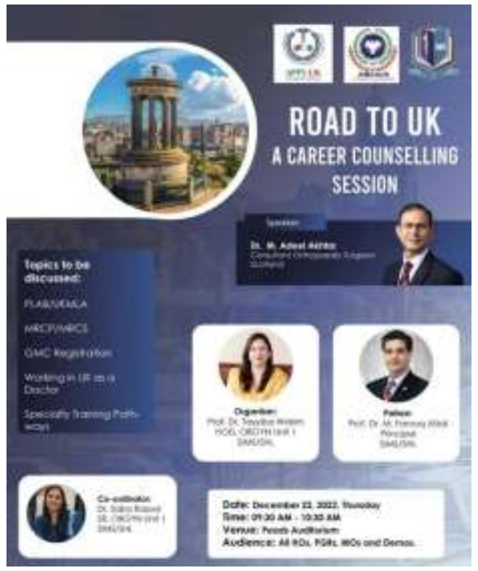<br>22nd and 6th November 2022 – CV writing skills for doctors applying to a master's programme in the UK - Today we did a session with middle grade and seniors doctors of Pakistan to guide them about the Master's Program in the UK being offered in many medical specialities .<br>Event was registered by more than 400 doctors and attended from Canada, UAE, Saudi Arabia and Pakistan .Many interesting points were discussed including eligibility criteria, cost of living ,university fee, GMC registration ,NHS working and further career progression in the UK.

					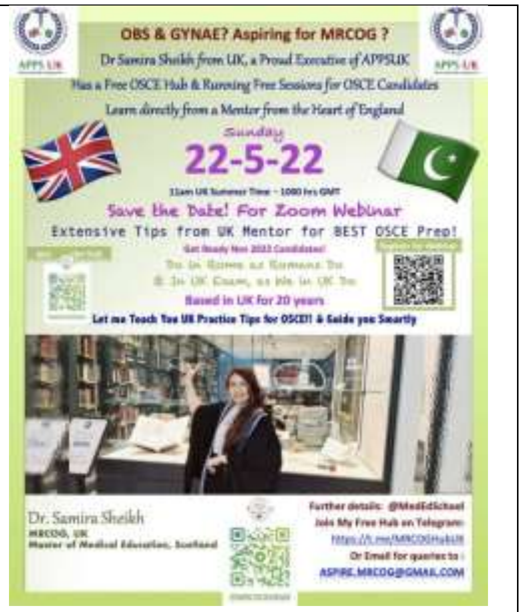<br>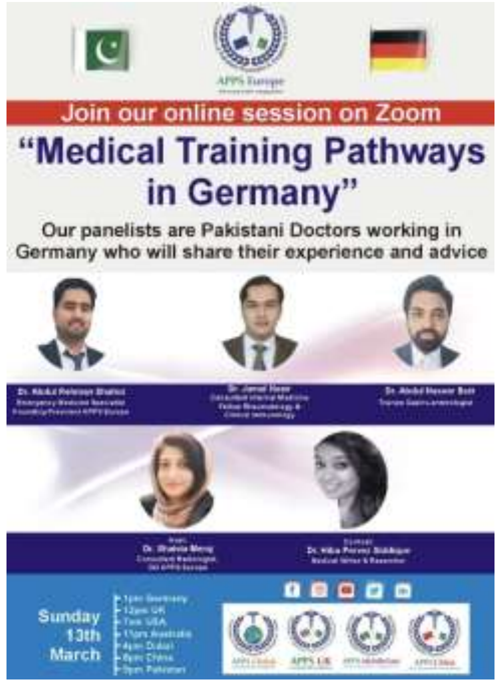<br>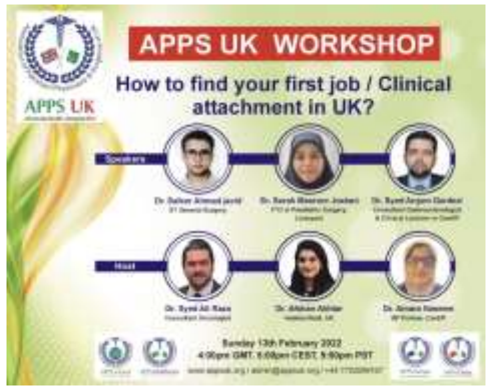<br>20th November 2021 - Registration of Overseas Medical Qualifications in Pakistan & Vice Versa webinar

					7th November 2021 - ADVANCED INTERVIEW TRAINING FOR DOCTORS: This session is intended for doctors who are applying for master's Programme in the UK, MTI or their first job in the NHS.<br><br>26th September 2021 – Writing an effective CV webinar<br><br>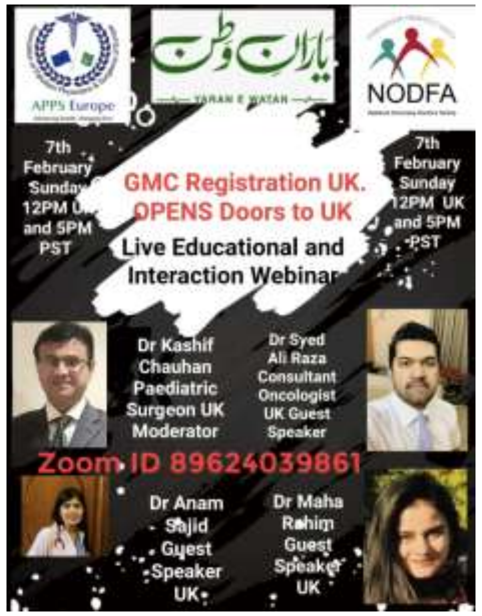
Association of Philippine Physicians in America	https://www.theappa.org/about	Its goal includes establishing continuing education program for physicians, providing aid for the education of physicians and medical students, supporting and stimulating medical research, and rendering free medical care for indigent people.	NA	13/10/22 APPA Gala and the CME speakers on X-Linked Dystonia-Parkinsonism – Conference<br><br>14/8/21 FilAm Health Forum Series #19: COVID-19 Updates – Vaccines and variants - Webinar<br><br>18/5/21 FilAm Health Forum Series #18: COVID-19 Updates – Vaccine allocation n the phllipines - Webinar<br><br>10/4/21 FilAm community members about their experiences with COVID-19 and the vaccine - Webinar<br><br>27/2/21 FilAm Health Forum Series 15: COVID-19 Vaccine Facts vs. Myths - Webinar<br><br>23/1/21 FilAm Health Forum Series#14: Cardiovascular Complications of COVID-19 – Webinar<br><br>10/1/21 FilAm Health Forum Series 13 – Covid 19 vaccine allocation	NA
Association of Physicians of Pakistani Descent of North America	https://appna.org/	The Association of Physicians of Pakistani Descent of North America is an American nonprofit organization headquartered in Westmont, Illinois, United States. It is one of the largest medical associations in the United States. APPNA is the largest medical organization of expatriate Pakistanis	NA	4-9th July 2023 - APPNA 46th Annual Convention	4-9th July 2023 - APPNA 46th Annual Convention

				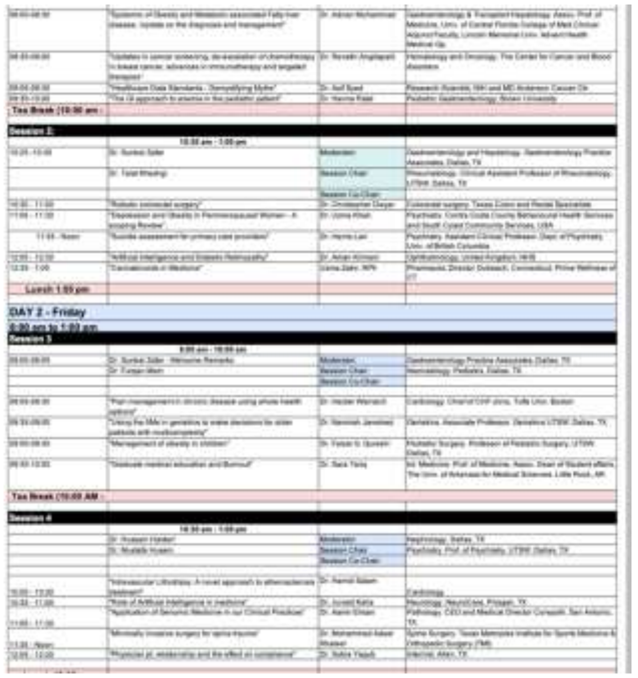<br><br>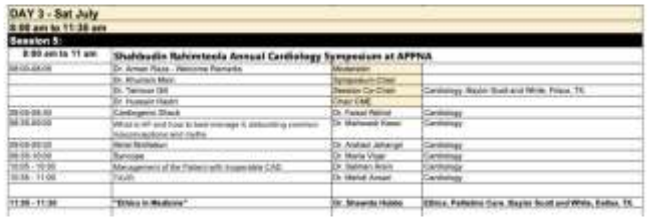 	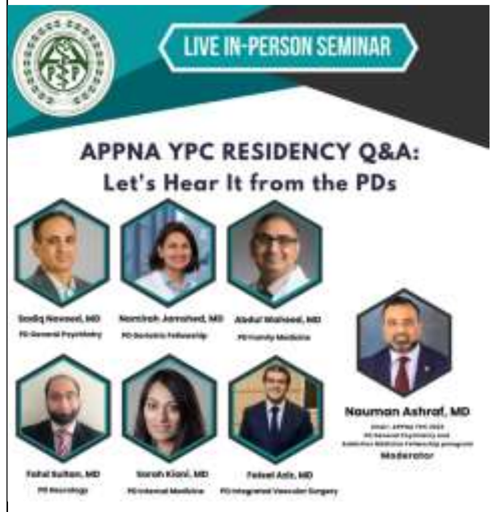<br><br>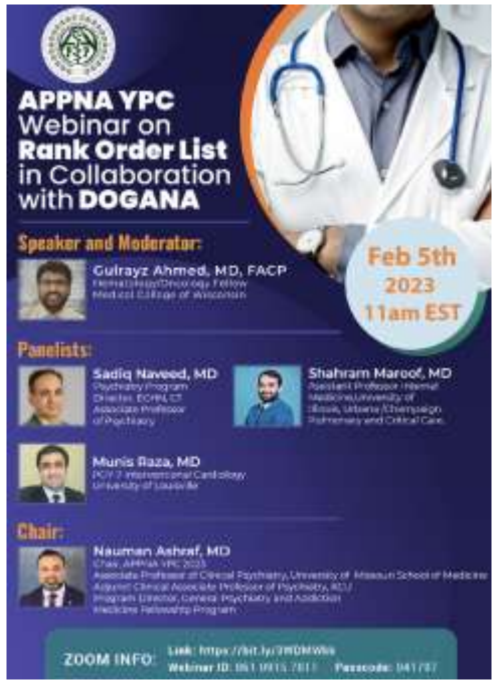<br><br>You are invited to attend a Workshop on "Writing a Personal Staten<br>Residency Application".<br>When: Aug 7, 2021 09:00 AM Central Time (US and Canada)<br>Register in advance for this meeting:<br>https://zoom.us/meeting/register/tJUlce6qrD0qGNJYPuB1aP2tmmm<br>After registering, you will receive a confirmation email containing inf<br>about joining the meeting.<br>Namirah Jamshed, MD<br>Chair, Young Physicians Committee

<table>
<tr><td></td><td></td><td></td><td></td><td>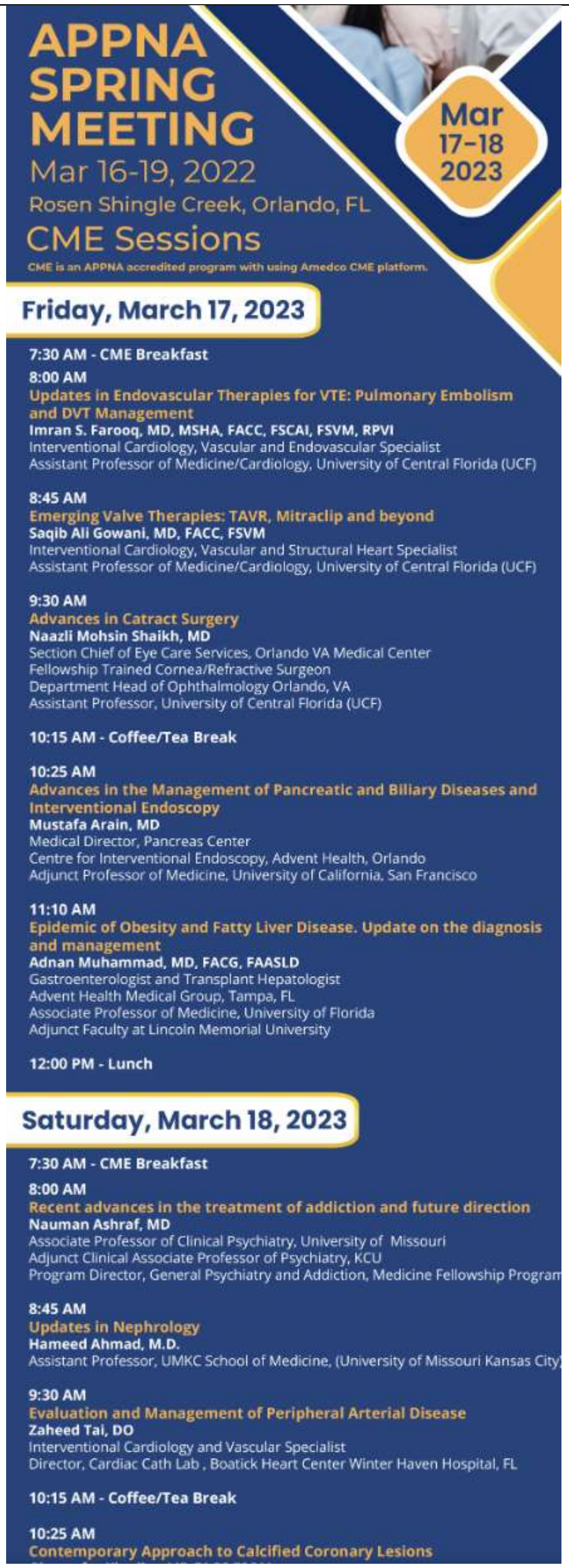
</td><td></td></tr>
</table>

				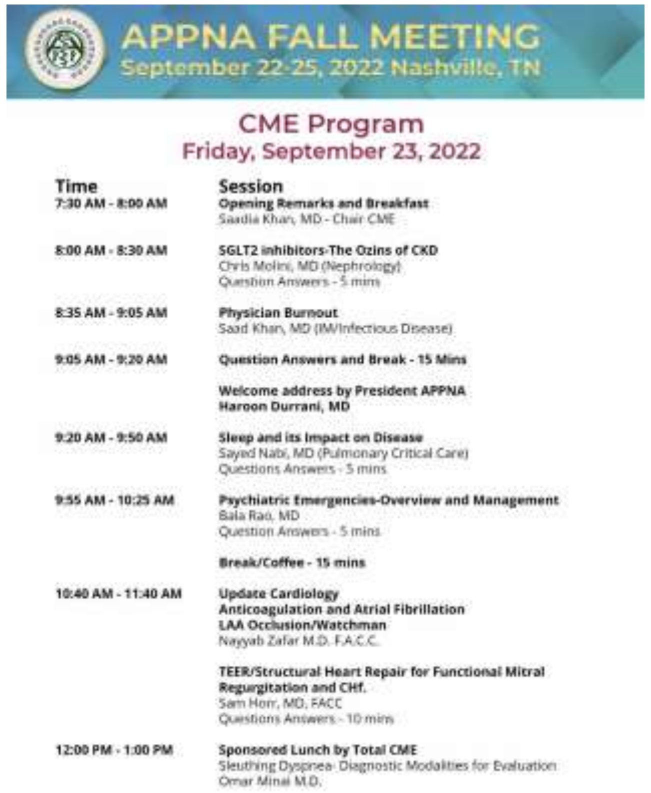	

APPNA FALL MEETING
September 22-25, 2022 Nashville, TN

CME Program
Friday, September 23, 2022

Time	Session
7:30 AM - 8:00 AM	**Opening Remarks and Breakfast**<br>Saadia Khan, MD - Chair CME
8:00 AM - 8:30 AM	**SGLT2 inhibitors-The Ozins of CKD**<br>Chris Molini, MD (Nephrology)<br>Question Answers - 5 mins
8:35 AM - 9:05 AM	**Physician Burnout**<br>Saad Khan, MD (IM/Infectious Disease)
9:05 AM - 9:20 AM	**Question Answers and Break - 15 Mins**
	**Welcome address by President APPNA**<br>**Haroon Durrani, MD**
9:20 AM - 9:50 AM	**Sleep and its Impact on Disease**<br>Sayed Nabi, MD (Pulmonary Critical Care)<br>Questions Answers - 5 mins
9:55 AM - 10:25 AM	**Psychiatric Emergencies-Overview and Management**<br>Bala Rao, MD<br>Question Answers - 5 mins
	**Break/Coffee - 15 mins**
10:40 AM - 11:40 AM	**Update Cardiology**<br>**Anticoagulation and Atrial Fibrillation**<br>**LAA Occlusion/Watchman**<br>Nayyab Zafar M.D. F.A.C.C.
	**TEER/Structural Heart Repair for Functional Mitral Regurgitation and CHf.**<br>Sam Horr, MD, FACC<br>Questions Answers - 10 mins
12:00 PM - 1:00 PM	**Sponsored Lunch by Total CME**<br>Sleuthing Dyspnea- Diagnostic Modalities for Evaluation<br>Omar Minai M.D.

				**CME Program**<br>**Saturday, September 24, 2022**<br><br>**Time** — **Session**<br>**7:30 AM - 8:00 AM** — **Opening Remarks and Breakfast**<br>Mohammad Farooq Ali, MD - Co-Chair, CME<br><br>**8:00 AM - 8:30 AM** — **Diabetes and South Asian Race**<br>Lubna Mirza, MD (Endocrinology)<br>Questions Answers - 5 mins<br><br>**8:35 AM - 8:55 AM** — **Covid Vaccines and Long Term Issues**<br>Humaira Qamar, MD (Pediatrics)<br><br>**Questions Answers and Coffee Break- 20 mins**<br><br>**9:15 AM - 9:45 AM** — **Update Cardiology**<br>**New Paradigm in Heart Failure /Role of SGLT2 Inhibitors**<br>Shoaib Akbar, MD, FACC (Cardiologist)<br>Questions Answers - 5 mins<br><br>**9:50 AM - 10:20 AM** — **Colon Cancer and Early Diagnosis**<br>Aimal Khan M.D.<br><br>**Questions Answers and Break - 20 mins**<br><br>**10:40 AM - 11:10 AM** — **Update on Hypothyroidism**<br>Nadia Yaqub, MD (Endocrinology)<br>Questions Answers - 5 mins<br><br>**11:15 AM - 11:45 AM** — **Shock in ICU**<br>Irfan Waheed, MD (Pulmonary and Critical Care)<br>Questions Answers - 5 mins<br><br>**12:00 PM - 1:00 PM** — **Sponsored Lunch by W. L. Gore & Associates, Inc.**<br>**TAVR**<br>Dr. Nayab Zafar	

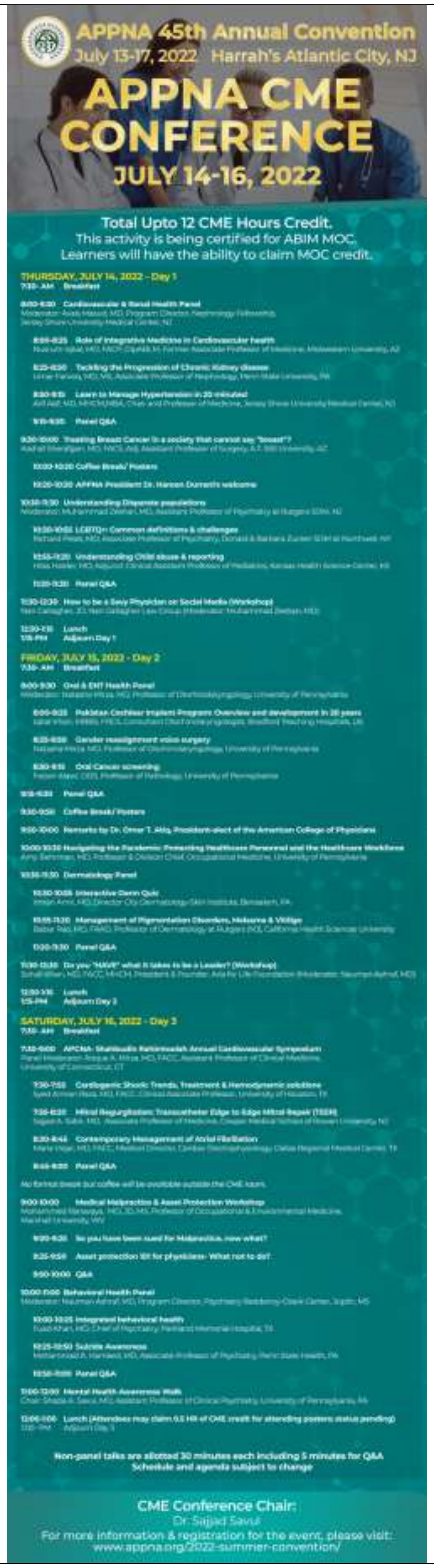
APPNA 45th Annual Convention
July 13-17, 2022 Harrah's Atlantic City, NJ
APPNA CME CONFERENCE
JULY 14-16, 2022
Total Upto 12 CME Hours Credit.
This activity is being certified for ABIM MOC.
Learners will have the ability to claim MOC credit.
CME Conference Chair:
Dr. Sajjad Savul
For more information & registration for the event, please visit:
www.appna.org/2022-summer-convention/

				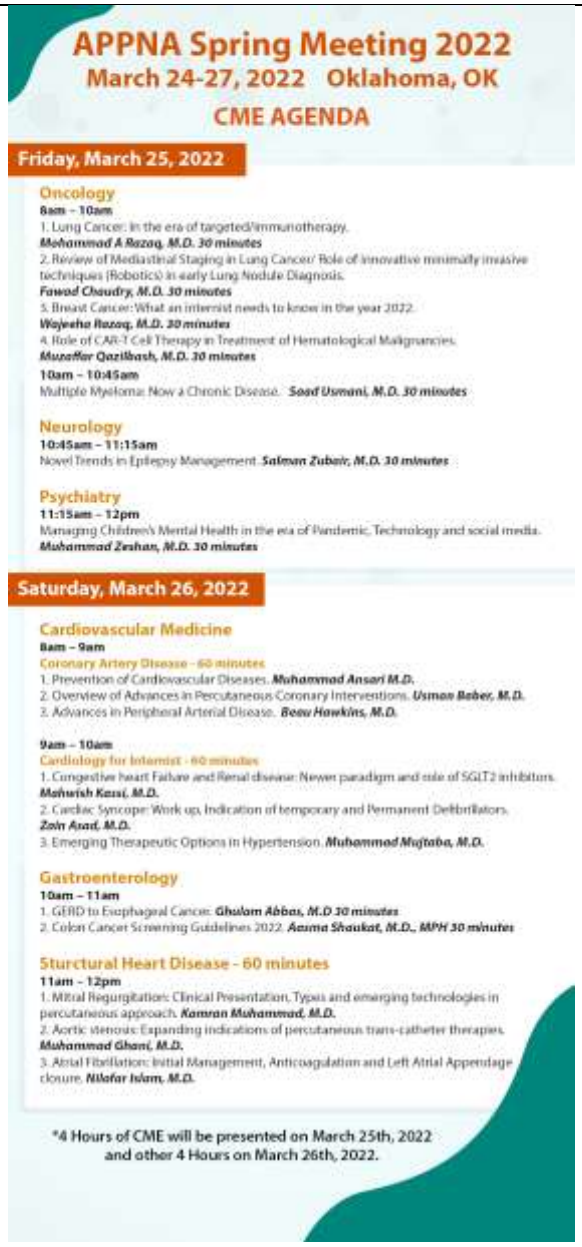	

APPNA Spring Meeting 2022
March 24-27, 2022 Oklahoma, OK
CME AGENDA

Friday, March 25, 2022

Oncology
8am – 10am
1. Lung Cancer: In the era of targeted/immunotherapy.
Mohammad A Razaq, M.D. 30 minutes
2. Review of Mediastinal Staging in Lung Cancer/ Role of innovative minimally invasive techniques (Robotics) in early Lung Nodule Diagnosis.
Fawad Chaudry, M.D. 30 minutes
3. Breast Cancer: What an internist needs to know in the year 2022.
Wajeeha Razaq, M.D. 30 minutes
4. Role of CAR-T Cell Therapy in Treatment of Hematological Malignancies.
Muzaffar Qazilbash, M.D. 30 minutes
10am – 10:45am
Multiple Myeloma: Now a Chronic Disease. *Saad Usmani, M.D. 30 minutes*

Neurology
10:45am – 11:15am
Novel Trends in Epilepsy Management *Salman Zubair, M.D. 30 minutes*

Psychiatry
11:15am – 12pm
Managing Children's Mental Health in the era of Pandemic, Technology and social media.
Muhammad Zeshan, M.D. 30 minutes

Saturday, March 26, 2022

Cardiovascular Medicine
8am – 9am
Coronary Artery Disease - 60 minutes
1. Prevention of Cardiovascular Diseases. *Muhammad Ansari M.D.*
2. Overview of Advances in Percutaneous Coronary Interventions. *Usman Baber, M.D.*
3. Advances in Peripheral Arterial Disease. *Beau Hawkins, M.D.*

9am – 10am
Cardiology for Internist - 60 minutes
1. Congestive heart Failure and Renal disease: Newer paradigm and role of SGLT2 inhibitors.
Mahwish Kazsi, M.D.
2. Cardiac Syncope: Work up, Indication of temporary and Permanent Defibrillators.
Zain Asad, M.D.
3. Emerging Therapeutic Options in Hypertension. *Muhammad Mujtaba, M.D.*

Gastroenterology
10am – 11am
1. GERD to Esophageal Cancer. *Ghulam Abbas, M.D 30 minutes*
2. Colon Cancer Screening Guidelines 2022. *Aasma Shaukat, M.D., MPH 30 minutes*

Sturctural Heart Disease - 60 minutes
11am – 12pm
1. Mitral Regurgitation: Clinical Presentation, Types and emerging technologies in percutaneous approach. *Kamran Muhammad, M.D.*
2. Aortic stenosis: Expanding indications of percutaneous trans-catheter therapies.
Muhammad Ghani, M.D.
3. Atrial Fibrillation: Initial Management, Anticoagulation and Left Atrial Appendage closure. *Nilofar Islam, M.D.*

*4 Hours of CME will be presented on March 25th, 2022
and other 4 Hours on March 26th, 2022.

<table>
<tr><td></td><td></td><td></td><td></td><td>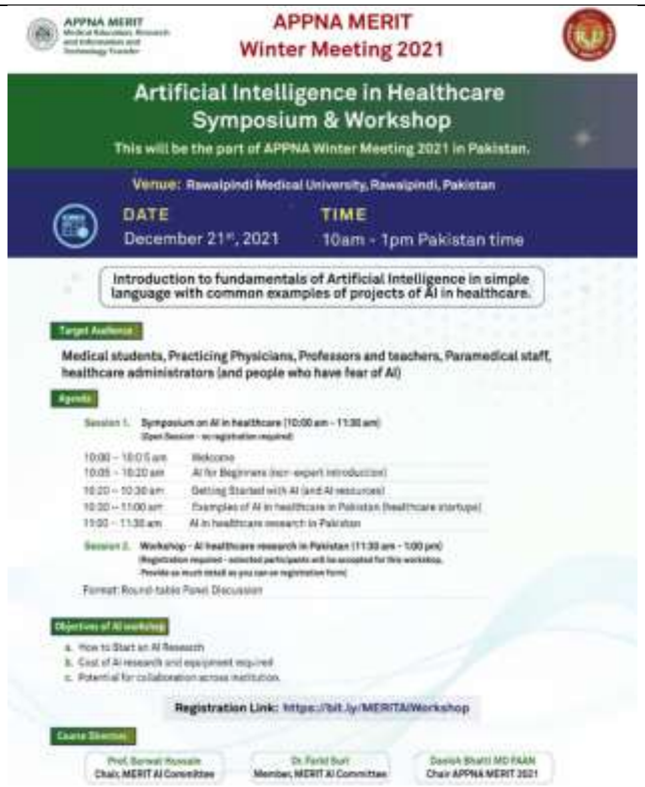

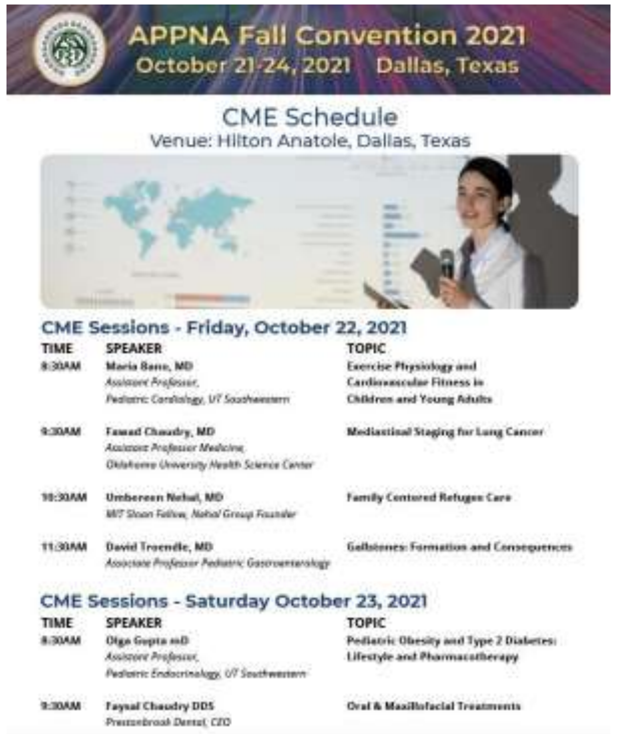
</td><td></td></tr>
</table>

				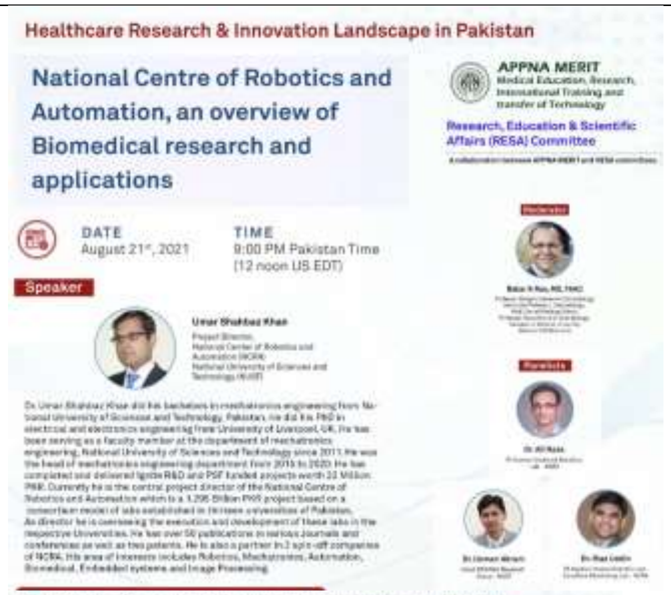Healthcare Research & Innovation Landscape in Pakistan<br>National Centre of Robotics and Automation, an overview of Biomedical research and applications<br>APPNA MERIT<br>Research, Education & Scientific Affairs (RESA) Committee<br>DATE<br>August 21st, 2021<br>TIME<br>9:00 PM Pakistan Time<br>(12 noon US EDT)<br>Speaker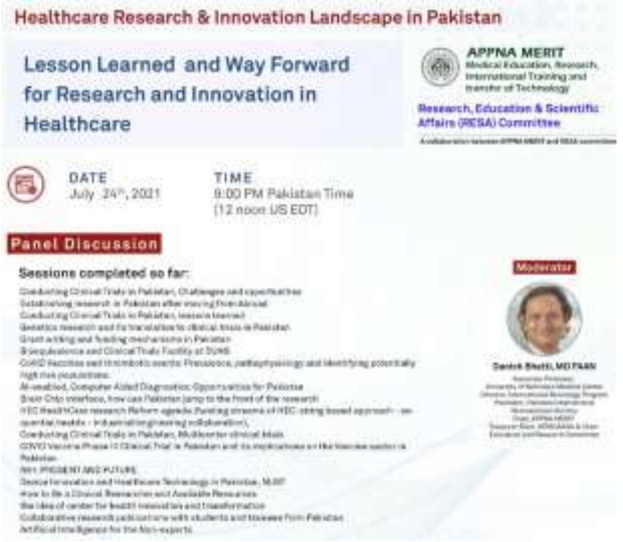Healthcare Research & Innovation Landscape in Pakistan<br>Lesson Learned and Way Forward for Research and Innovation in Healthcare<br>APPNA MERIT<br>Medical Education, Research, International Training and transfer of Technology<br>Research, Education & Scientific Affairs (RESA) Committee<br>DATE<br>July 24th, 2021<br>TIME<br>9:00 PM Pakistan Time<br>(12 noon US EDT)<br>Panel Discussion<br>Sessions completed so far:<br>Moderator	

				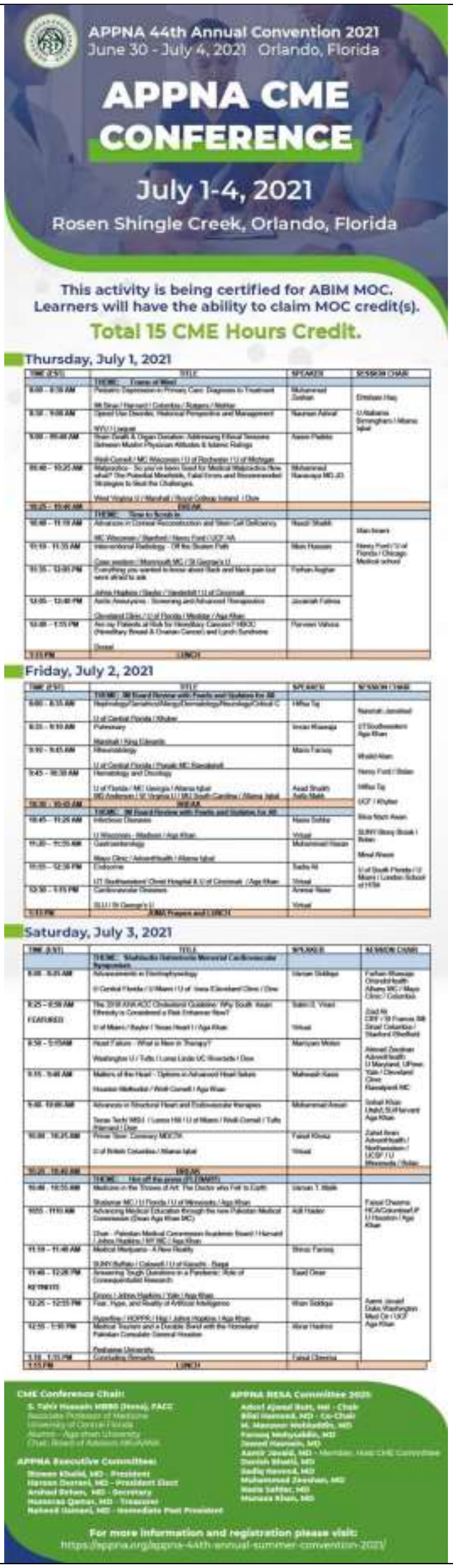<br>APPNA 44th Annual Convention 2021<br>June 30 - July 4, 2021 Orlando, Florida<br>APPNA CME CONFERENCE<br>July 1-4, 2021<br>Rosen Shingle Creek, Orlando, Florida<br>This activity is being certified for ABIM MOC.<br>Learners will have the ability to claim MOC credit(s).<br>Total 15 CME Hours Credit.<br>Thursday, July 1, 2021<br>Friday, July 2, 2021<br>Saturday, July 3, 2021		

				Healthcare Research & Innovation Landscape in Pakistan - 24th, 25th April 2021<br><br>MERIT Health Care Research in Pakistan – Webinar on April 17th 2021	
Australia Myanmar Medical Association	http://www.au-mma.org	To support newly (or) recently arrived medical professionals from Myanmar<br><br>To promote health and wellbeing of Myanmar Community both in Victoria, Australia and in Myanmar<br><br>To promote health and wellbeing of Victorian Community by collaborating with other health organisations and medical associations<br><br>To provide a support network for Myanmar Medical Professionals in Victoria, Australia	NA	28/07/23 - Approach to abnormal leukocyte count online session<br><br>29/06/23 how I approach a patient with a headache online session<br><br>30/03/23 - Management of minor orthopaedic injuries online session<br><br>23/02/23 - management of type 2 diabetes in general practise online session<br><br>24/11/22 - when to refer to a cardiologist online session<br><br>27/10/22 - pain management online session	25/04/23 - "RACGP Clinical Examination Q&A Session". Experienced RACGP online session - Experienced RACGP clinical examiners as well as fresh RACGP fellows will be facilitating the session as panel members. It is a great learning opportunity for the exam candidates.
Bangladesh Medical Association of North America	https://www.bmana.org/	BMANA is a nonprofit, nonpolitical, educational and charitable organization of medical professionals of Bangladeshi descent. BMANA was incorporated in Michigan in 1981. Since then 18 chapters of BMANA have been established across the United States.<br>In addition to educational, cultural and charitable events organized by the individual chapters, BMANA sponsors a national convention annually. 37 national conventions have been held in a variety of North American cities. The conventions feature a unique blend of educational, cultural, social and humanitarian activities.	NA	27/08/23 - How to approach and evaluate abnormal Liver Function webinar	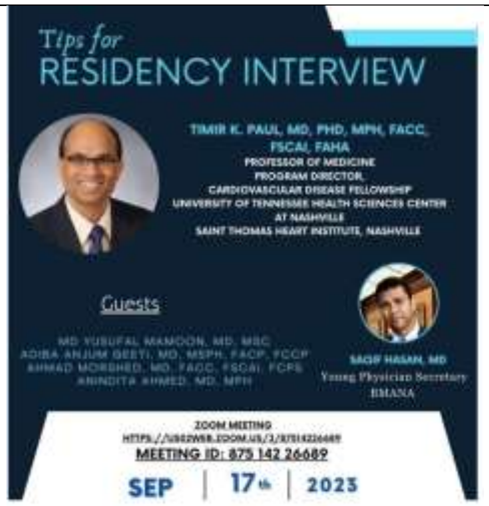<br><br>06/08/23 - ERAS Application: All you need to Know | Live Webinar
Bangladesh Medical Association UK	https://www.bdmauk.org/	To promote medical and allied sciences<br>To look after the interest of members of the association<br>To promote understanding among the medical organisations<br>To provide mentoring, counselling and career advice<br>To share with members both happy and unhappy occasions<br>To foster a close link with the parent association in Bangladesh	NA	5/09/21 - Mental health awareness symposium following COVID recovery	NA
Bolivian American Medical Society	http://www.bolivianamericanmedicalsociety.com	• To form an organization of Bolivian physicians in the United States to promote and facilitate professional relations between and among the members of the organization and all members of the medical profession.<br>• To provide for the continuing education of the members of the organization by means of lectures, panel discussions, seminars, publications, and the like.<br>• To support and assist Bolivian medical education, and to make grants and gifts in aid to Bolivian medical schools and Bolivian medical students.<br>• To further the education of Bolivian medical students by means of lectures, panel discussions, and seminars sponsored by the organization and by distributing various educational materials to Bolivian medical students.<br>• To support and assist medical education in general.	NA	NA	NA
British Association of Physicians of Indian Origin	https://www.bapio.co.uk	To realise the potential of our members in achieving leadership & professional excellence.<br>To effectively partner with statutory, professional and voluntary organisations to improve the delivery of healthcare to patients.<br>To support members in developing and improving their career prospects.	NA	18/03/23 – BAPIO Conference – Heart disease, diabetes management and obesity management<br><br>01/11/22 – Optimising GP practise resources webinar<br><br>4-6th Nov 2022 – National conference<br>- Understanding research: a walk through the stages	4-6th Nov 2022 – National conference<br>- Existing Indian International Migration activity

<table>
<tr>
<td></td>
<td></td>
<td>To encourage members to make use of their knowledge of culture and languages in promoting better health awareness amongst ethnic communities.
To monitor, highlight and address the difficulties faced by doctors and to ensure systems to support them.
To monitor, highlight and address the difficulties faced by nurses and to ensure systems to support them.
To promote activities of charitable nature that would help towards alleviating social deprivation and disadvantage.
To enable members to experience Indian heritage through social and cultural events.</td>
<td></td>
<td>- Transforming a research question into a study
- How to write a good paper and get it published
- Inequalities on mental health
- Overdiagnosis and Overtreatment are causing the Medicalisation of trivial illnesses
- Pathways to recovery from COVID-19
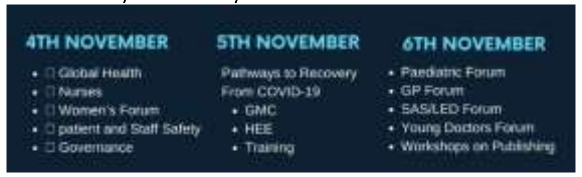

18th July 2022, 17/06/23 - "On-Call" Teaching Series 'Haematology' online

15/05/22 - On-Call series session on Clinical Orthopaedic Exams

28/04/22 - On-Call series session on 'Urological Emergencies'

22/04/22 - On-Call series session on Paediatric A-E Assessment

01/03/22 - "On-call teaching series" on "General Surgery Emergencies"

01/12/21 - BAPIO East of England Educational Online Development
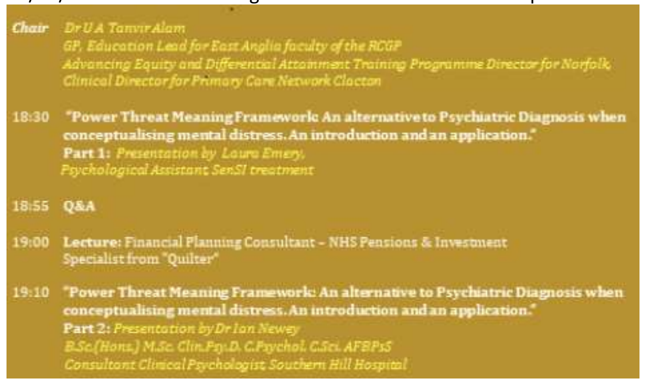

07/04/21 - BAPIO East of England Educational Online Development Sessions
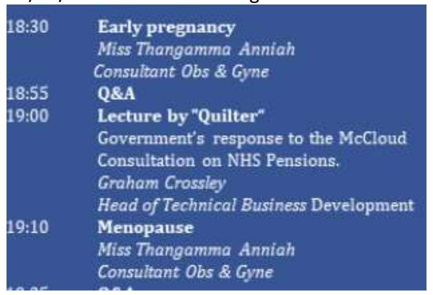
</td>
<td>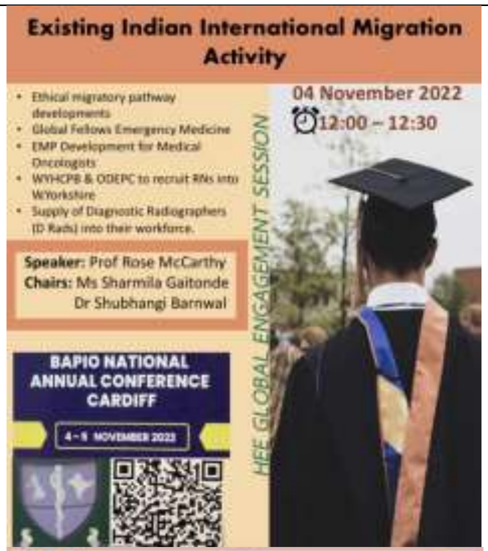

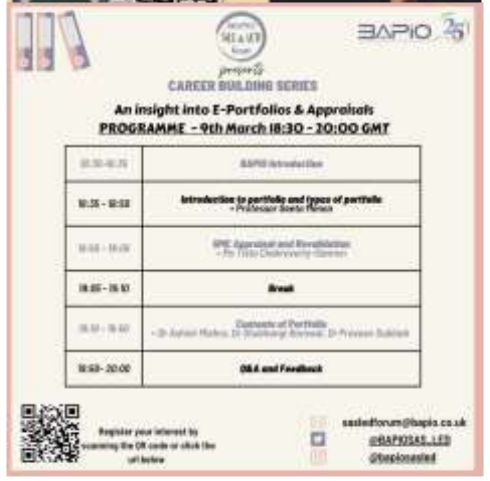

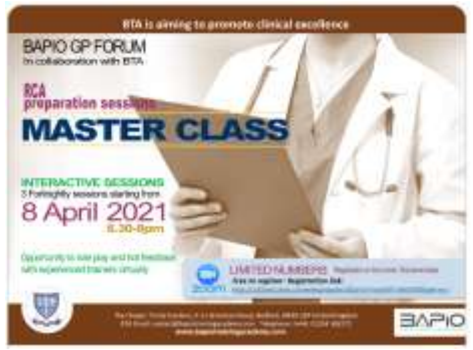
</td>
</tr>
</table>

				**BAPIO Wales Annual Conference Saturday 30th January 2021** – Cardiff<br>09.30 Meeting opens for panellists<br>10.00 Welcome – **Dr Hasmukh Shah** BEM , Secretary BAPIO (Wales)<br>10.05 Chairman's address – **Prof. Keshav Singhal** MBE, Chair BAPIO (Wales)<br>10.15 First Minister of Wales – **Rt Hon. Mark Drakeford AM**<br>10.30 Keynote lecture – **Prof Lord Ajay Kakkar,** Chair King's Fund<br>10.45 Signing of MOU – **BAPIO and Cardiff & Vale** University Health Board<br>11.00 Racial inequalities during Covid – **Jane Hutt AM,** Dy Minister, Welsh Government<br>11.15 Advancing Medical Professionalism during the Covid 19 Pandemic – **Prof. Iqbal Singh OBE** Chair CESOP<br>11.30 The Health Scenario in Wales Post Covid – **Dr Andrew Goodall CBE**- DGHS & Chief Executive NHS Wales<br>11.45 The All Wales Covid-19 Risk Assessment Tool – **Dr Heather Payne** Senior Medical Officer Wales<br>12.00 Launch of CMO Special Report; Protecting our Health during the First Phase of Covid-19 – **Dr Frank Atherton** Chief Medical Officer Wales<br>12.15 International recruitment– HEIW & BAPIO – **Prof Push Mangat Medical Director HEIW**<br>12.30 Covid & Fitness to practise – **Anthony Omo, General Counsel & Director-Fitness to practise General Medical Council**<br>12.40 Immigration and Global Medical Workforce – **Rt.Hon Alun Cairns MP**<br>12.55 High Commissioners' Address – **Her Excellency Gaitri Issar Kumar** High Commissioner of India to the UK<br>13.10 Tackling inequalities during and post Covid Role of BAPIO and its members – **Dr Ramesh Mehta OBE** President BAPIO<br>13.25 Break<br>13.45 Wales & India– Health Partnership – **Vaughan Gething AM** Health Minister of Wales<br>14.00 Covid in India– a success story – **Hon. Dr Harshvardhan MP,** Union Health Minister of India<br>14.15 Collaboration & Exchanges in Medical Education between India & UK – **Lt. Gen (Dr) MD Venkatesh VSM** Vice Chancellor Manipal Academy of Higher Education<br>14.30 The Oxford Vaccine – **Dr Adaar Poonawala CEO Serum Institute of India**<br>14.45 **Panel Discussion**<br>**1. Covid-19 Pandemic: What could we have done better**<br>**Moderator: Prof Parag Singhal, National Secretary BAPIO, Consultant Endocrinologist**<br>Prof Andrew Goddard, President Royal College of Physician (UK)<br>Dr David Bailey BMA Welsh Council Chair<br>Judge Ray Singh CBE<br>Dr Hasmukh Shah BEM Secretary BAPIO (Wales) Visiting Fellow University of South Wales<br>**2. Is there a silver lining behind the pandemic**<br>**Moderator: JS Bamrah CBE Chairman BAPIO UK**<br>Prof Martin Marshall CBE, Chair Royal College of General Practitioners, UK<br>Dr CDV Jones CBE, Chair HEIW<br>Dr Phil White, Chair General Practitioners Committee BMA Wales<br>Dr K Jagadeesan FRCS President International Medical Sciences Academy	
British Iranian Medical Association	https://www.facebook.com/BIMAUK	A multidisciplinary healthcare network catering for British-Iranians all over the UK	NA	17/05/23 – Inspiring Iranians in healthcare event – cardiothoracic surgery, diagnosis and treatment of oesophageal and gastric cancer, robotic surgery	NA
British Islamic Medical Association for Sudanese descent	THIS ORGANISATION CANNOT BE FOUND	NA	NA	NA	NA
Burmese American Medical Association	http://www.bamausa.org	At BAMA our number one goal is to welcome and support our new, up and coming cohort of medical doctors from Myanmar, to prepare them for residency application, and conduct a residency information session for the applicants.	NA	NA	17/08/23 – Residency mock interviews<br>**BAMA Residency Mock Interview 2023**<br>This is the time to welcome and support our new coming medical doctors from Myanmar, preparing them for residency application. We would like to conduct residency mock interview for the applicants according to the following schedule.<br>Date and time: August 19th, 2023 (Saturday), 1:00 PM – 4:00 PM PST (US time)<br>Location: Zoom meeting room<br>13/05/23 – Residency information evening

					**BAMA Residency Info 2023**<br>Date: **May 13th, 2023 (Saturday)**<br>Time: **1:00 PM – 4:00 PM PST (US& Canada)**<br>Place: **Zoom**<br>Host & Co-Host: Dr. Sanda Win, Dr. Kyaw Khaing Soe, Dr. Than Saung Lin<br>**Agenda**<br>1:00 – 1:20 PM: Opening speech by BAMA president Dr. Edna Taikwel<br>1:20 – 1:40 PM: How to prepare USMLE exam by Dr. Wai Wai Mon<br>1:40 – 2:00 PM: How to prepare OET exam by Dr. Nyan Lin Aung<br>2:00 – 2:20 PM: Steps to apply residency by Dr. Kay Zin Min Latt<br>2:20 – 2:40 PM: Ways to get ECFMG approval by Dr. Kyaw Khaing Soe<br>2:40 – 3:00 PM: How to get letter of recommendation by Dr. Hla Thwe<br>3:00 – 3:20 PM: How to get clinical observership and rotation by Dr. Hnin Ei Khine<br>3:20 – 4:00 PM: Q&A<br><br>27/08/22 - Residency mock interview<br>**BAMA Residency Mock Interview 2022**<br>This is the time to welcome and support our new coming medical doctors from Myanmar, preparing them for residency application. We would like to conduct residency mock interview for the applicants according to the following schedule.<br>Date and time: August 27th, 2022 (Saturday), 1:00 PM – 4:00 PM PST (US time)<br>Location: Zoom and Dr. Edna Taikwel's office at Garvey Healthy Family Medical clinic (701 E Garvey Ave, Monterey Park, CA 91755). We could meet at Dr. Taikwel's office at 12:30 pm on Aug 27th for those faculty and interviewers who would like to have in-person interview.<br>- **Future residents**: please RSVP to bamausaorg@gmail.com before August 19th, 2022 along with your curriculum vitae (CV) in ERAS format, personal statement, specify specialties you are applying and recommendation letters (if any), and please mention in-person or zoom interview.<br>- We have American-trained college graduates who are willing to proof-read the personal statements for you. If you want them to review your personal statement, please specify it and submit your personal statement no later than August 19th, 2022. First-come, first-serve. Please limit only one statement per candidate.<br><br>11/06/22 – Residency information session 2022<br>**BAMA Residency Information Session 2022**<br>Date: June 11th, 2022 (Saturday)<br>Time: 1:00 PM – 4:00 PM PST (US & Canada)<br>Virtual Meeting via Zoom<br>Host & co-host (Dr. Sanda Win, Dr. Kyaw Khaing Soe, Dr. Than Htaik Kyaw)<br>1. Opening speech by Dr. Htun Tin (President of BAMA)<br>2. USMLE exam preparation by Dr. Ei Tun<br>3. USMLE registration and ECFMG certification pathways by Dr. Kyaw Zaw Hein.<br>4. ERAS preparation by Dr. Shoon Lei Oo.<br>5. Interview preparation by Dr. Yi Mon Tun.<br>6. Experience of matching into pathology residency by Dr. Nyein Htun<br>7. Questions and Answers with panel discussion<br><br>29/08/21 – Residency mock interviews 2021<br>**BAMA Residency Mock Interview**<br>Date: Aug 29, 2021 (Sunday)<br>Time: 1 to 4 pm (Pacific time)<br>Location: Zoom<br>Host: BAMA Education Committee<br>• Please RSVP at bamausaorg@gmail.com **before Aug 15, 2021**, along with your **resume**.<br>• Please also send personal statements if they need to be edited.<br>• Please submit USMLE score reports (both step I and II) if you are applying BAMA academic achievement award.<br>• Eligibility criteria for the award:<br>a) For anyone who took USMLE both step I and II.<br>b) Please reapply if you have not received monetary award before.<br>c) Top 5 applicants with highest score who took exams within last 3 years.<br><br>22/05/21 – Residency information session 2021

					**BAMA Residency Information Session**<br><br>**Date: May 22, 2021 (Saturday)**<br>**Time: 3:00 pm – 5:00 pm Pacific Time (US and Canada)**<br><br>**Virtual Meeting via Zoom**<br>Host & Co-host (Dr. Wai Wai Soe, Dr. Sanda Win)<br>1. Opening Speech (Dr. Su Su Hline)<br>2. Residency Statistics (Dr. Kyaw Khaing Soe)<br>3. Panel Discussion by Residents Matched in 2021<br>Dr. Thaw Thaw Han<br>Dr. Su Wai Moe<br>Dr. Kyaw Moe Naing<br>4. Interview during Covid Crisis (Dr. Steven Lin)<br>5. Questions and Answers
Burmese Doctors and Dentists Association UK	https://www.facebook.com/BDDAUK/?ref=page_internal	This is an non-profit, non-religious and non-political organisation. Our aim is to provide professional and social support to doctors and dentists.	NA	NA	NA
Burmese Medical Association Australia	https://www.facebook.com/profile.php?id=100064518231260	To have a united group of Burmese medical, dental and all health professionals, not only based in NSW	NA	NA	NA
Burmese Medical Association of North America	http://bma-na.com	The Burmese Medical Association of North America is a non-profit organization founded in July 1991 with the purpose to promote the welfare of persons of Burmese heritage in the medical profession, to assist physicians and healthcare professionals as they arrive from Burma with professional examinations, job placement and to secure the benefits of personal acquaintance.	NA	NA	NA
Cambodian Health Professionals Association of America	https://www.chpaa.org	CHPAA was founded by a group of physicians, dentists, pharmacists, physician assistants, nurses, allied health professionals, and volunteer community members. The vision of CHPAA is to promote health and well-being of the underserved in Cambodia and the U.S., free of charge.	NA	NA	NA
Cameroon Doctors UK	https://www.camdocuk.org/home	A forum for Medical Doctors and Dentists of Cameroonian origin working in the UK to meet and share ideas about various aspects of healthcare provision in UK and Cameroon.<br><br>Providing professional expertise to issues relating to Healthcare Development and Promotion in Cameroon.<br><br>Providing an environment where mentorship and constructive debate can flourish.<br><br>Forging partnerships with stakeholders in Cameroon Healthcare such as Medical Schools, Hospitals, NGOs etc	NA	30/09/23 - Mens health, all you need to know webinar<br><br>24/06/23 - Fool proof strategy to live a healthy life webinar<br><br>19/11/23 - Autism all you need to know webinar<br><br>26/06/22 - Annual scientific conference - Vulnerability and Resilience: Optimising potential for active ageing - audits and research projects, childhood nutrition, adult wellbeing and national development in africa<br><br>28/05/22 - Mens health mind over matter webinar<br><br>19/02/22 - menopause what you need to know webinar<br><br>17/07/21 - physical activity and exercise in COVID 19 webinar<br><br>12/06/21 - COVID-19 and women's health fibroids webinar<br><br>24/04/21 - Mental health during covid-19 webinar<br><br>27/03/21 - COVID 10 vaccination webinar	NA
Canadian Association of Nigerian	http://canpad.org	CANPAD shares a common vision of "A Healthier Nigeria in a Healthier World". The strength in numbers of people with similar vision, mission, goals and foresight coupled with unparalleled	NA	04-05/08/23 - Annual conference<br>- Current trends in disability medicine<br>- Racism in medicine and the impact on mental health<br>- addiction impact across specialities	29/07/22 - Annual conference<br>- The Nigerian Trained Doctor & Regulatory Bodies (a 2-hour Symposium)

Physicians and Dentists		collective reasoning, altruism and love of our dear country is what keeps us together and drives us to do more.		- diabetes and CKD management<br>- AI in medicine<br>- mental health Q+A and update<br>- pitfalls in the referral consultation process	31/10/21 - IMG chronicles Q+A<br>- An interactive Q & A session with Dr. Shayee about all things related to the International Medical Graduate program.
Chinese American Medical Society	http://chineseamericanmedicalsociety.cloverpad.org	It is the mission of CAMS to improve the health status of Chinese Americans and to promote excellence in health care for all through the mobilization of health care professionals.<br><br>The purposes & objectives of the Society are as follows:<br><br>1. To eliminate health disparities of Chinese Americans and other underserved populations.<br>2. To advance medical knowledge through education, scientific research, scholarship, and philanthropy with emphasis on aspects unique to Chinese Americans.<br>3. To promote the association of and to advocate for medical professionals of Chinese descent and others devoted to like purposes.	12/10/21 - CAMS Medical Student Career Week Presents: Barriers to Care For Asian Patients	06/11/22 - 59th Annual Conference - Championing medicine and public health for our communities<br>- New Innovative Directions in Opthalmic Telemedicine, AI, & Remote Monitoring<br>- How to Create Your Culinary Medicine Rx<br>- Lung Cancer- Current Recommendations and Future Directors<br>- Food as Medicine: East Meets West<br>- Public Health: Asian American<br>- RESEARCH UPDATES ON POST-ACUTE SEQUELAE OF SARS-COV-2<br><br>4/10/22 - Journal club presentation - Charting a Path Towards Asian American Cancer Health Equity: A Way Forward<br><br>21/07/22 - Journal club presentation - Sarcoidosis among chinese-americans and the evolving landscape of sarcoidosis<br><br>22/06/22 - Long Life CME Lecture - Current Trends in Suicide Prevention: Science, Culture, Implementation<br><br>30/03/22 - Long Life CME Lecture - impact of microaggressions and racism on the AAPI community<br><br>12/04/22 - The CAMS Journal Club will be discussing "The importance of community and culture for the recruitment, engagement, and retention of Chinese American immigrants in health interventions,"<br><br>22/03/22 - CAMS Journal Club will be discussing "Heart to Heart Cards: A Novel, Culturally Tailored, Community-Based Advance Care Planning Tool for Chinese Americans,"<br><br>26/01/22 - CAMS Journal Club - "Health Related Social Needs Among Chinese American Primary Care Patients During the COVID-19 Pandemic: Implications for Cancer Screening and Primary Care,"<br><br>17/11/21 - Physician mental health: preventing suicide and building resilience among our own webinar<br><br>10/11/21 - Long Life CME webinar on Social Isolation & Its Impact<br><br>06-07/11/21 - Annual scientific conference<br>- preparing for the post-pandemic world<br>- epidemiology<br>- health care policy and ageing research<br>- population health sciences<br>- Immunizations<br><br>29/09/21 - Long Life CME program - Building Resilience Through Relationship-Centred Communication<br><br>08/06/21 - Rheumatic diseases and covid-19 research updates webinar<br><br>20/05/21 - Long Life CME program - direct care workers: optimising health for vulnerable communities	NA
Chinese American Physicians Society	http://www.caps-ca.org	The Chinese American Physicians' Society (CAPS) was formed by a group of concerned physicians of Chinese descent in 1977 in the San Francisco area. Our current membership consists of various ethnic groups and races. We are one of the corporate members of the Federation of Chinese American and Chinese Canadian Medical Societies (FCMS).<br><br>Our main objectives:	NA	NA	NA

		1: To eliminate, eradicate and discourage racial prejudice and discrimination in all facets of the medical profession.<br><br>2: To encourage, foster and stimulate a greater awareness of the social responsibility of the medical profession to the community.<br><br>3: To provide a forum for the exchange and discussion of information concerning problems, new advancements and improvement of skill relating to the field of medicine.<br><br>4: To advocate, promote and encourage the expansion and availability of low-cost medical services in socioeconomically disadvantaged communities			
Dominican Medical Association	http://www.dmanewyork.com	ALL CONTENT ON THE WEBSITE AND SOCIAL MEDIA WERE IN SPANISH	ALL CONTENT ON THE WEBSITE AND SOCIAL MEDIA WERE IN SPANISH	ALL CONTENT ON THE WEBSITE AND SOCIAL MEDIA WERE IN SPANISH	ALL CONTENT ON THE WEBSITE AND SOCIAL MEDIA WERE IN SPANISH
Egyptian American Medical Association	http://www.e-ama.org	The Egyptian Medical Association (EAMA) is a nonprofit, non political, educational and humanitarian organization of medical, dental, pharmaceutical and allied health professionals of Egyptian descent and other nationals who graduated from Egyptian Universities.	NA	NA	NA
Egyptian American Medical Society	http://www.egyptianamericanms.com	Advancement of medical science and to offer aid to the people of egypt<br>Advancement of medical science and exchange of medical ideas	NA	NA	NA
Egyptian Association for American Medical Training and Research	www.eamtar.com	EAMTAR is a non-profit organization dedicated to the advancement of Egyptian medical students and physicians in the U.S.	NA	NA	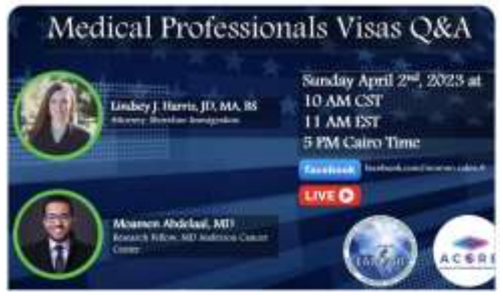<br>25/03/23 - Dr.. Khaled Elsayes will speak in a Live Video on his page about "The Path to Practicing Medicine in the United States" and will be followed by a group of sessions.<br>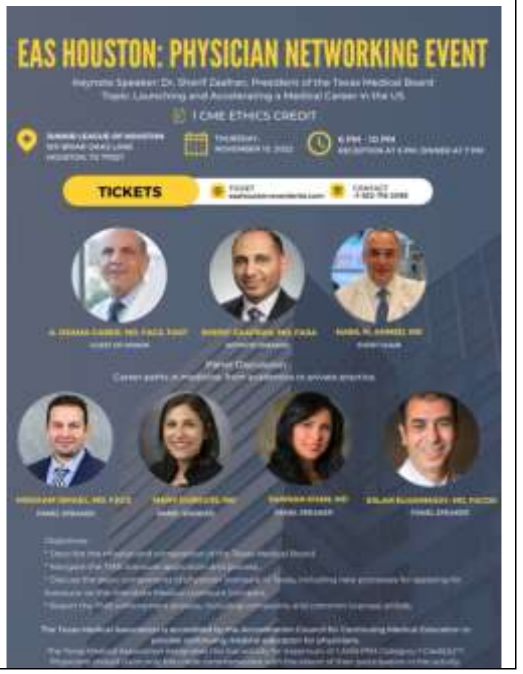

					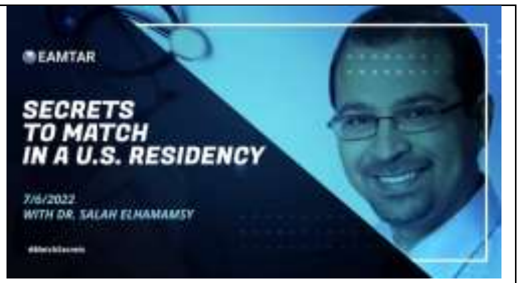<br><br>Topics to be discussed:<br>1. Secrets to matching in a U.S. Residency.<br>3. How Programs choose between their applicants.<br>3. How to find U.S. Clinical experience.<br>4. How to make yourself more competitive.<br>5. How to write your CV, EARS application and personal statement.<br>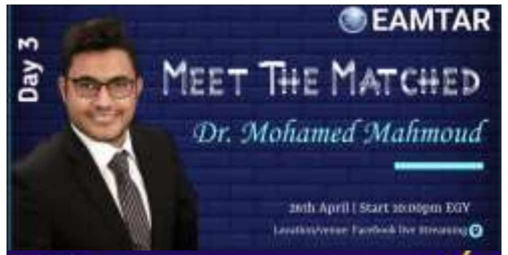<br><br>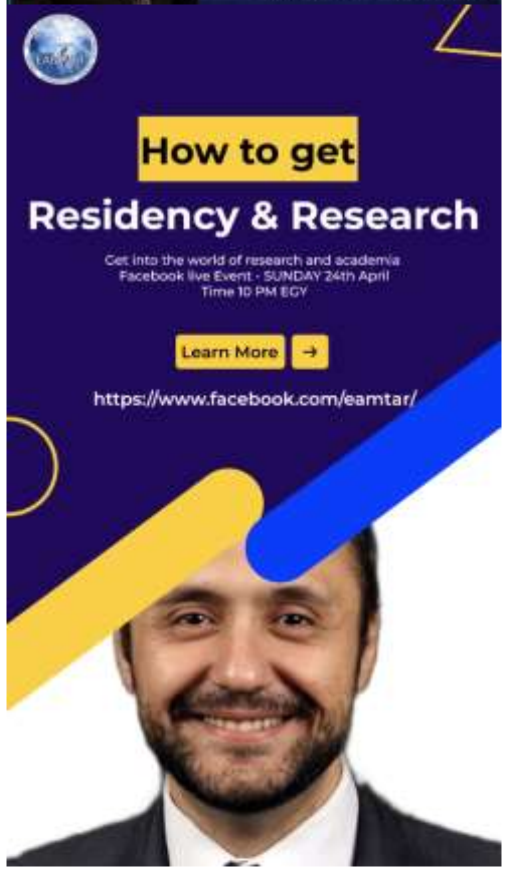<br><br>19/04/22 - live open discussion session at 12:00 AM (Cairo time) with Dr. Athar Eysa<br>-Internal Medicine incoming resident at Morristown Medical Center, 2022<br>-Menoufia University Graduate 2015<br>- Research scholar, Yale University.<br>17/04/22 - Meet The Matched WOMEN Physician 2022

					1- Dr. Athar Eysa:<br>Internal Medicine incoming resident at Morristown Medical Center, 2022.<br>2- Dr. Alaa Elzayat:<br>-pediatric resident at Texas Tech University, Amarillo, Texas<br>3-Dr. Aya Allam:<br>Family medicine resident, Creighton University, Nebraska 2022.<br>4- Dr. Bothaina Afifi, M.D:<br>Family medicine Resident at Reid Health, KCU-GME consortium, Richmond, Indiana.<br>5- Dr. Hadeer ELtahan:<br>-Family Medicine resident at University of North Dakota.<br>6-Dr. Monica Ibrahim:<br>Pediatric resident at University of Florida.<br>7- Dr. Shaymaa Elhadidy:<br>Child Neurology resident at Nationwide Children's Hospital.<br><br>18/04/22 - Meet The Matched WOMEN physician 2022 (Day 2).<br>Meet the speakers :<br>1-Dr. Alaa Elzayat<br>-pediatric resident at Texas Tech University, Amarillo, Texas<br>2- Dr. Aya Allam:<br>Family medicine resident, Creighton University, Nebraska 2022<br>3- Dr. Sarah Elsayed<br>Matched in Internal Medicine Program at North Alabama Medical Center.<br>4- Dr. Sara Abdelgawad<br>PGY1 Psychiatry resident at Texas Tech University Health science center (TTUHSC) Lubbock<br>5- Dr. Reem Kadry:<br>Pathology Resident at Indiana University 2022
Egyptian Medical Society UK	http://www.egyptianmedical.org.uk	Since its inception over 30 years ago our Society has been at the forefront of supporting the Egyptian Medical community in the UK and a forum for strenghtening expertise and community. As a leading medical Society, we work to unite doctors of Egyptian origin in the UK. Egyptian Medical Society UK is run by members for its members, offering a united, amplified voice for all who join our community.	NA	23/09/23 - Annual scientific day<br>- Differential attainment and neurodiversity: impact on career progression<br>- Diabetes: modern advances in prevention and current research<br>- Teamwork and non-technical skills for medical practitioners<br>- International collaboration: a realistic view on collaboration between Egypt and the UK NHS<br><br>17/09/22 - Annual scientific day<br>- TAVI talk<br>- Creating a culture of innovation in healthcare organisation<br>- The WHO checklist revised<br>- Advances in neurosurgery<br>- Human factors	NA

				- AI and medicine<br>- Junior doctor presentations<br><br>25/07/22 - Scientific day - Patient Safety and Innovation in Modern Medical Practice	
Ethiopian North American Health Professionals Association	http://enahpa.org	Its mission is facilitated by these key areas:<br><br>Engaging in direct medical outreach via medical/surgical missions, orphan support and established preventative care programming<br><br>Transferring of knowledge by means of distance learning, medical training and sponsoring international medical fellowships for Ethiopian health providers<br><br>Cultivating and grooming the Young Adult Council to sustain a lasting legacy<br><br>Help mitigate the profound health care needs of Ethiopia and Africa through the transfer of knowledge, skills, state of-the-art technology, research and development	NA	NA	NA
Ghana Physicians and Surgeons Foundation	https://www.ghanaphysicians.org	The Ghana Physicians and Surgeons Foundation of North America (GPSF) was established to support the mission of the Ghana College of Physicians and Surgeons (GCPS) which was established by an Act of Parliament in 2002 to provide quality postgraduate training in Medicine in Ghana. It also serves as an advocacy group to influence health policy and educate the general public about numerous health care issues in Ghana and Africa. The foundation supports all non-profit health initiatives in Ghana by Ghanaian and other health professionals in North America.<br>This is also a United State registered 501(c) entity Diaspora.	5/09/2023<br>Join us on Tuesday, September 5 2023 for session about ERAS application.<br>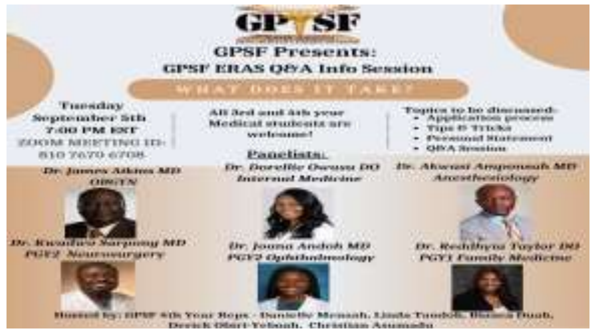<br><br>19/03/2023<br>USMLE Study Tips<br>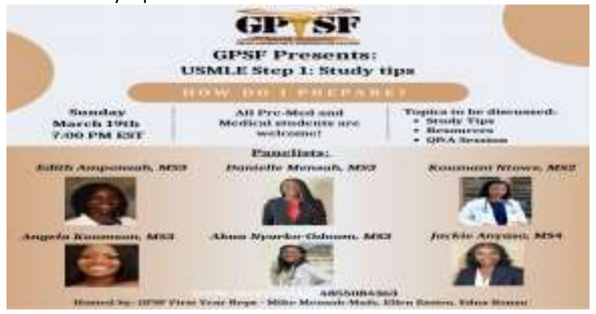<br><br>27/01/2023-<br>You are invited to a Zoom webinar.<br>When: Jan 27, 2023 07:00 PM Eastern Time (US and Canada)<br>Topic: Education Series - Interventional Procedures for the Aging Population	21-23rd April 2023- 20th Annual Conference<br>Theme: Reducing Childhood And Maternal Morbidity And Mortality By 2035<br>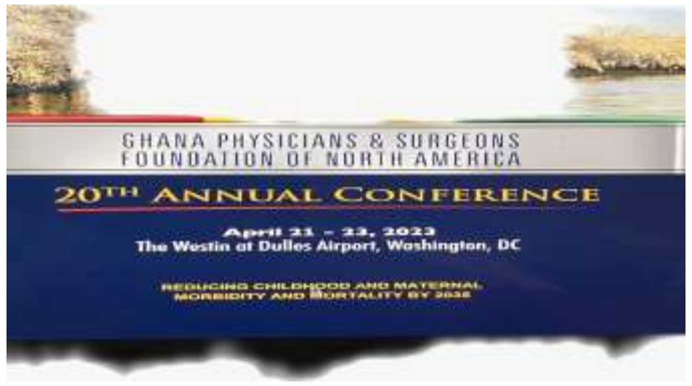<br><br>29th-30th Sept. 2022<br><br>This is the flagship conference organized by the Ghana College of Physicians and Surgeons in Collaboration with the Ghana Physicians and Surgeons Foundation of North America (GPSF).	22nd March 2023<br>You are invited to a Zoom webinar.<br>When: Mar 22, 2023 08:00 PM Eastern Time (US and Canada)<br>Topic: Career Journey Series - Dr. Asare Christian<br>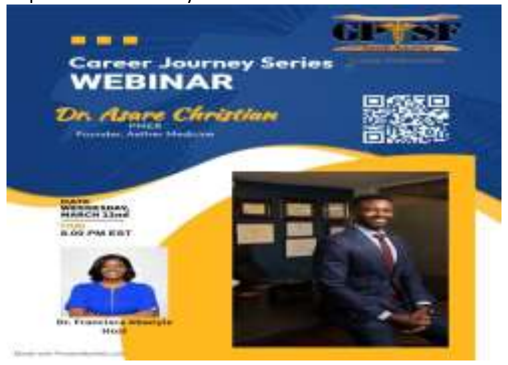<br><br>Jan 25, 2023-<br>You are invited to a Zoom webinar.<br>Topic: Career Journey Series - Dr. Paa-Kofi Obeng<br>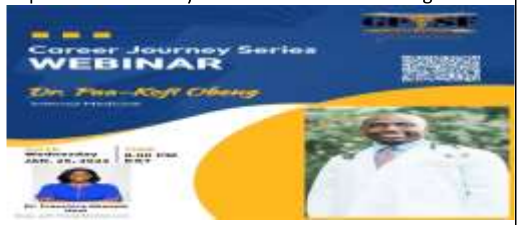<br><br>Jul 27, 2022- 8:00 PM Eastern Time (US and Canada)<br>Topic: Career Journey Series<br>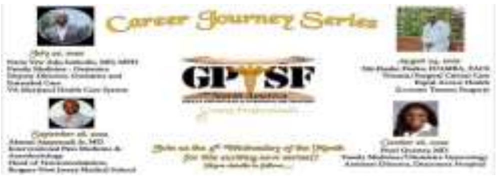<br><br>28th Sept. 2022<br>Career Journey Series happening NOW. Tell a friend to join in When: Wednesday September 28, 2022 8:00 PM

			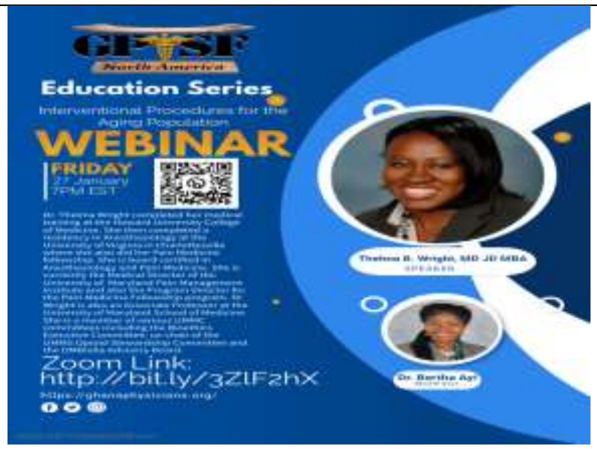<br>25th Aug 2022<br>Are you preparing for Step 1?<br>Join us this Sunday August 28th at 5pm EST for a crash course on this exam! Get your questions and concerns about this exam answered by members who have already taken and passed it!<br>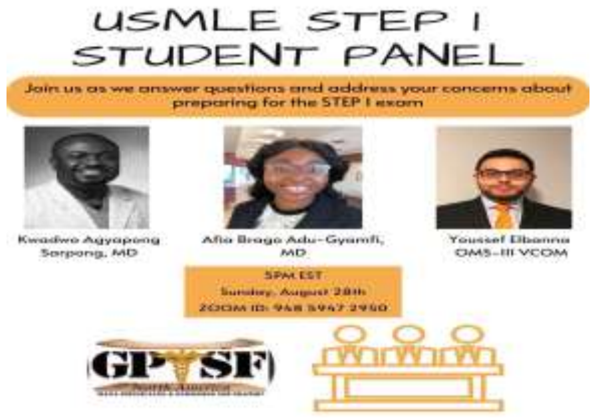<br>24th August 2022-<br>Wednesday August 24, 2022 8:00 PM Eastern Time (US and Canada) Topic: Career Journey Series Please click the link below to join the webinar:<br>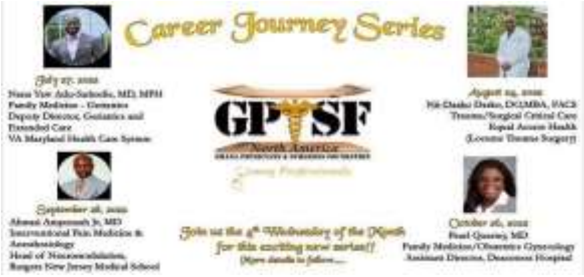<br>29th July 2022<br>Join us tomorrow at 3pm for tips on how to crush your secondary applications and get those interview invites!	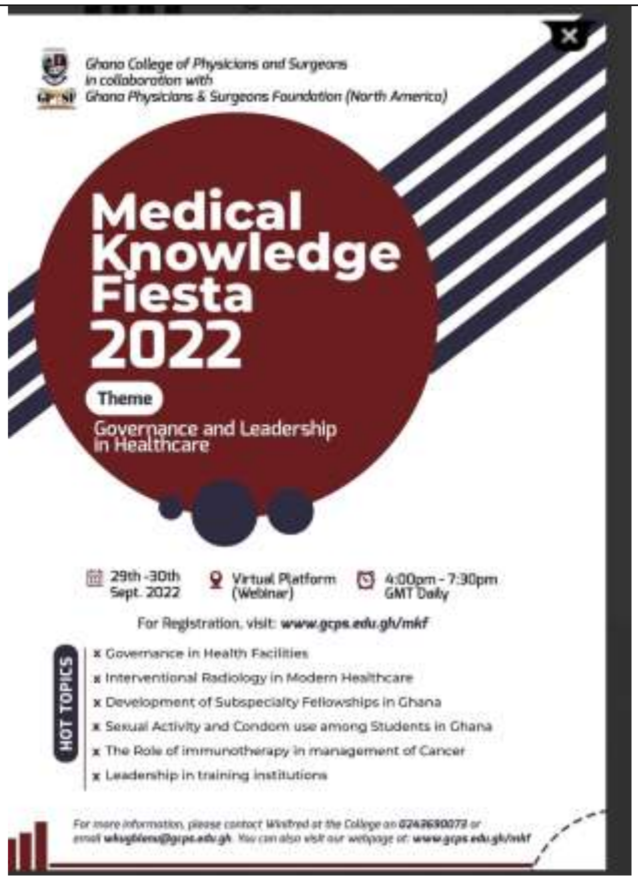<br>25th June 2022<br>Are you a little nervous about interviewing patients? Well fear not! Join us TOMORROW, June 25th join us as we give you tips and tricks on how to effectively interview, interact, and present patient cases!<br>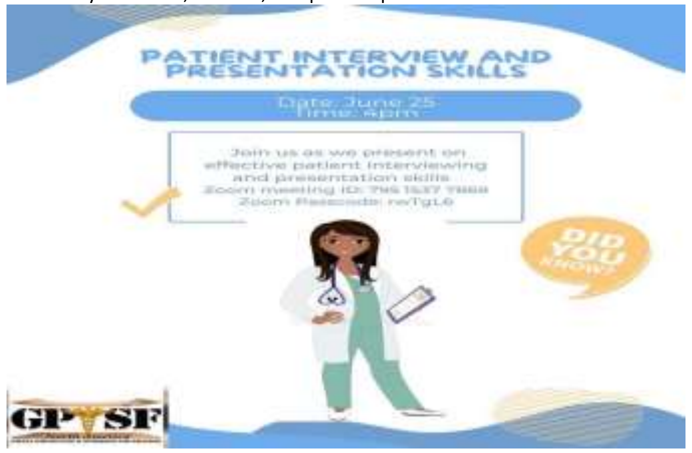<br>25th March 2022,<br>You are invited to a Zoom webinar.<br>Topic: GPSF Educational Webinar Series - Chronic Pain	Eastern Time (US and Canada) Topic: Career Journey Series<br>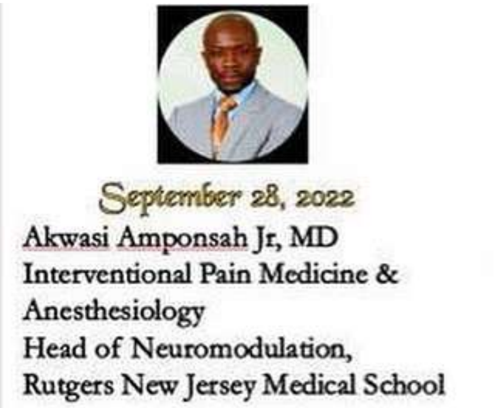<br>1st Feb 2021- Residency Rank Listing Meeting Webinar (Explaining how ranking works and how to prepare for it)<br><br>7th Sept 2021-Some tips for medical students and IMGs applying for residency this year from Dr. Maxie Afari and Dr. Yvonne Berko. Zoom conference originally hosted on 9/5/2021<br>https://youtu.be/7dg2FqOG0og<br><br>Some tips for medical students and IMGs applying for residency this year from Dr. Maxie Afari and Dr. Yvonne Berko. Zoom conference originally hosted on 9/5/2021<br>Some tips for medical students and IMGs applying for residency this year from Dr. Maxie Afari and Dr. Yvonne Berko. Zoom conference originally hosted on 9/5/2021st<br>nnn

			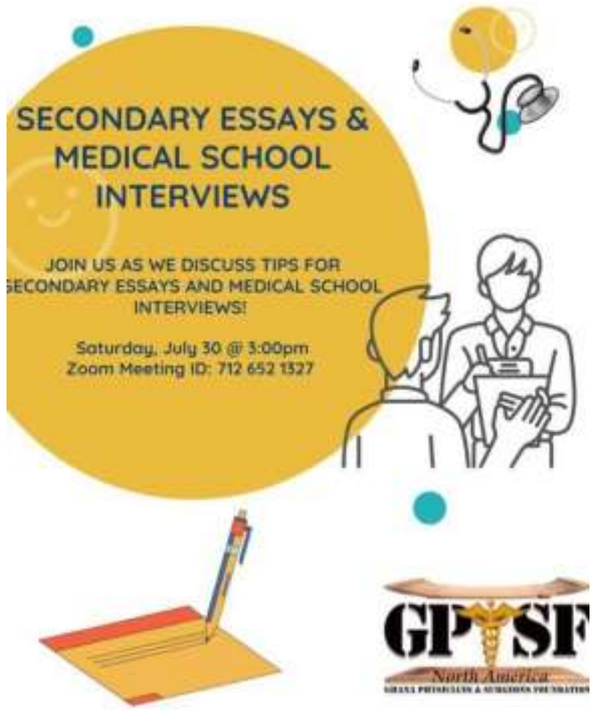<br><br>7th July 2022<br>ERAS season is quickly approaching! Join us this Thursday, July 7th at 8PM ET, to get all the TEA on how to make your application stand out! Get all your questions answered by our newest residents who successfully matched this year!<br>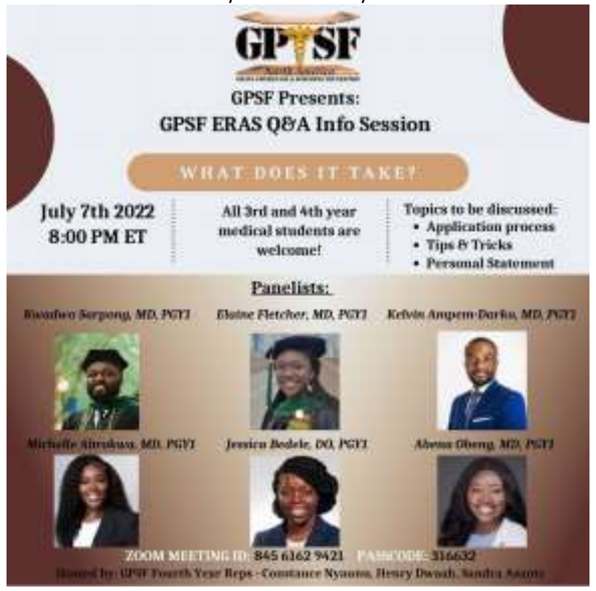<br><br>248h June 2022<br>THE 1st YEAR SURVIVAL GUIDEPlease join GPSF on June 28th at 7 pm EST as we discuss how to survive the first year of medical school with our extraordinary panelists! 💊🩺 We will take the time to answer any questions you may have about how to navigate study techniques, research opportunities, extracurriculars, and other related subjects.📚	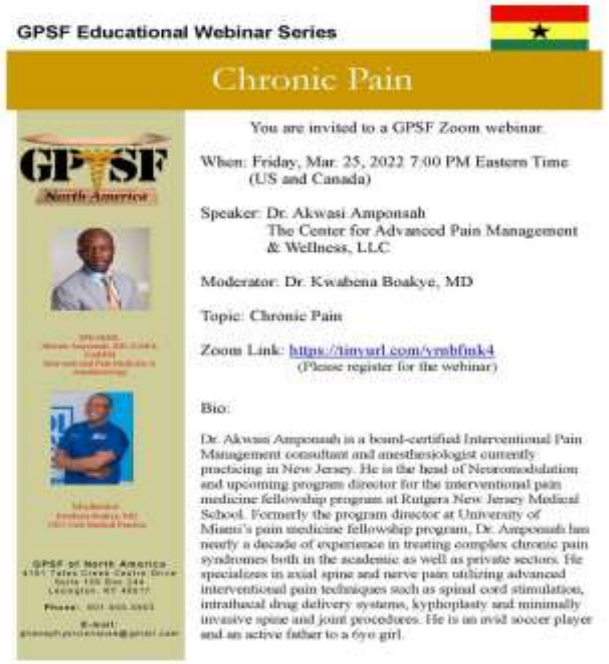<br><br>22nd-24th April 2022<br>19th Annual Conference<br>Theme:Optimizing Healthcare in Post Covid Pandemic Era<br>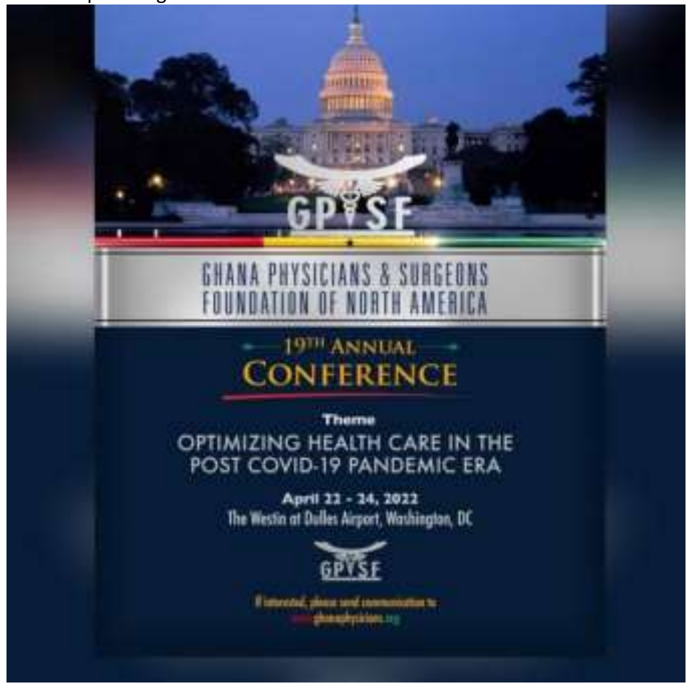<br><br>22/10/2021-<br>Development of Maternal-Fetal Medicine in Low-Middle Income Countries: the case of Ghana by Prof. Timothy RB Johnson, MD, FACOG FWACS FICS FRCOF FGCS, Zoom Webinar	

			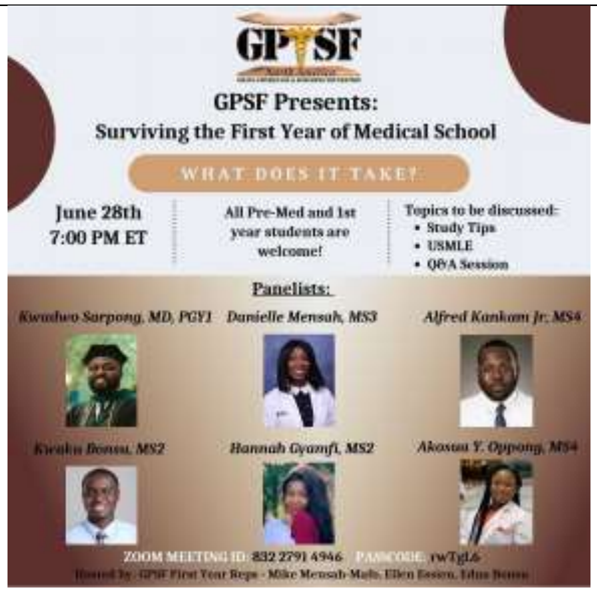		
Ghanaian Doctors & Dentists Association	https://www.gddauk.org	The Ghana Physicians and Surgeons Foundation of North America (GPSF) was established to support the mission of the Ghana College of Physicians and Surgeons (GCPS) which was established by an Act of Parliament in 2002 to provide quality postgraduate training in Medicine in Ghana. It also serves as an advocacy group to influence health policy and educate the general public about numerous health care issues in Ghana and Africa. The foundation supports all non-profit health initiatives in Ghana by Ghanaian and other health professionals in North America.<br>One of their core activities is the planning and undertaking of missions to Ghana to deliver medical supplies and medical care to underserved communities.	NA	9th Oct. 2021<br>Our Virtual Zoom GDDA-UK Annual Health Conference and AGM is finally due next week Saturday 9th October 2021 from 10am - 1pm.<br>Time: 10am – 1pm<br>Moderators (Zoom): Dr Tony Annan, Dr Sefa Aggrey, Dr Conrad Buckle<br>Theme: Covid-19 Pandemic –<br>➢ The Ghanaian Experience<br>➢ UK Innovative ways of working & Learning<br>10:00am Welcome: Dr William Kedjanyi, President GDDA-UK<br>10:10am Covid-19 Pandemic – The Ghanaian experience of the virus and vaccines. Prof William AMPOFO<br>10:55am Surveillance strategy and management protocol of Covid-19 patients in Ghana. The National Vaccination Programme and Ghanaian Response to it. – Prof Ernest KENU<br>11:40am New & Innovative ways of Working & Learning in the light of Covid-19 Pandemic. Dr Conrad Buckle<br>12:30pm Q&A and Discussion<br>12:45 pm Closing Remarks<br>1:00 pm AGM	NA
Iranian American Medical Association	https://www.iama.org	The IAMA organization is established for charitable and educational purposes, for assisting students in the field of medicine financially and for providing healthcare assistance to needy Iranians.IAMA strives to establish Continuous Medical Education each year during the annual meeting and publishes the associated activities in the IAMA Bulletin to fulfill its' educational obligation. IAMA strives to establish Continuous Medical Education each year during the annual meeting and publishes the associated activities in the IAMA Bulletin to fulfill its' educational obligation.	NA	NA	NA
Iranian Medical Society	http://www.iranianmedicalsociety.org/Index.cfm	Founded in 1986 by a group of medical doctors for professional medical advancement and to enhance the Iranian Culture.<br>Their Goals are to gather Iranian American Physicians and facilitate each other with knowledge through each own individual specialties	NA	16th Nov 2022:- Educational Meeting - Surgical Treatment of Obesity	NA

		for the purpose of better treatment and patient's care, enhance the Iranian culture. An ancient culture based on loving and kindness and learning encourage-ment from birth to death.To break a linguistic barrier for the elderly Iranian patients and introduce a Farsi speaking physician to help and support their needs.<br>To organize a scientific meeting annually for CME for the purpose of:<br>a. To update their knowledge and skills for better patient's care.<br>b. To fulfill the CME requirement for the validation of medical license.<br>To find different specialties among our fellow members for a better, fast and convenient service to the patients.<br>To get CME accreditation for our society for the purpose of organizing a monthly meeting to update physician's skill and knowledge and treat patients with the latest medical surgical development.		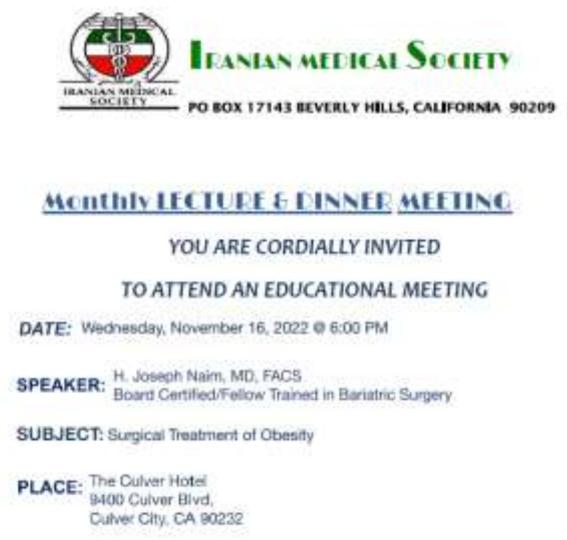<br><br>14th July 2022:-Educational Meeting<br>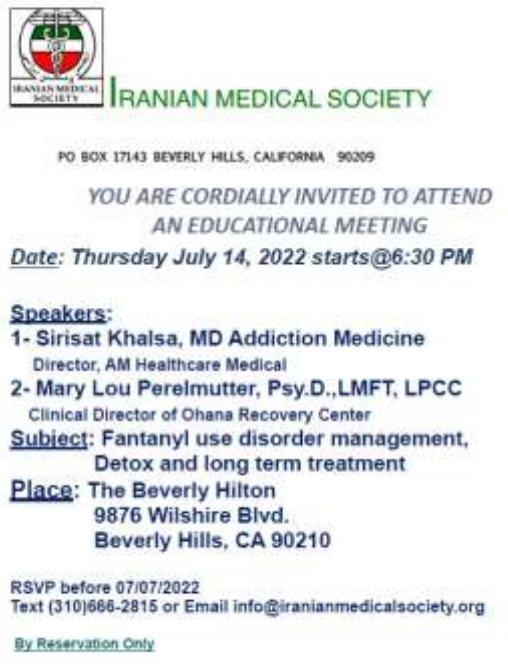<br><br>5th May 2022:-Educational Meeting	

<table>
<tr><td></td><td></td><td></td><td></td><td>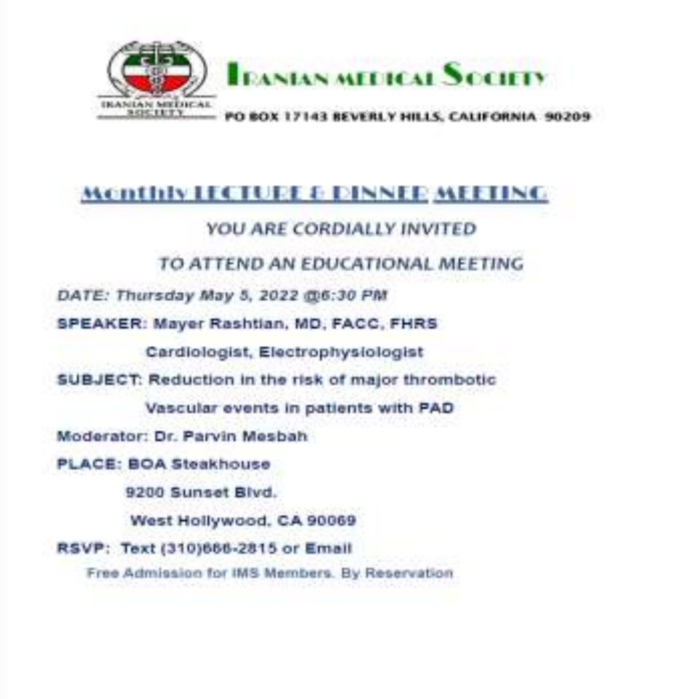
</td><td></td></tr>
<tr><td>Iranian Medical Society UK</td><td>http://www.iranianmedicalsociety.org.uk</td><td>The Iranian Medical Society (IMS) is the only registered charity in the UK with the aim of supporting the Persian speaking medical community in the UK.The society provides opportunities to network at professional, social and cultural levels and help its members to develop both personally and professionally and achieve the highest professional and moral standards. In addition, the society serves to provide overseas aid to provide relief, and also offer advice and support to the public, with particular reference to Iranian and Persian speaking community in the UK.</td><td>NA</td><td>15th May 2021
Virtual Lecture Series
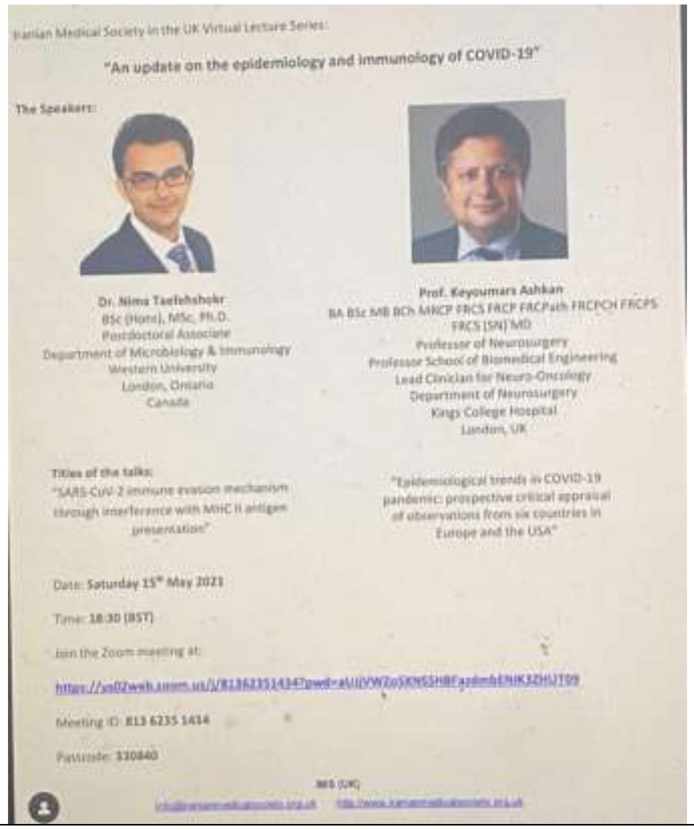
</td><td>NA</td></tr>
</table>

Iranian-American Medical Society of Greater Washington (Iranian Medical & Dental Society of the Greater Wash-Balt Metro Area)	http://www.iamsgw.org	NA<br><br>(Found but no recent information within inclusion criteria time frame)	NA	NA	NA

Iraqi Medical Association UK	https://www.imauk.com	NA<br>(not found/functioning)	NA	NA	NA
Iraqi Medical Sciences Association USA	https://www.imsausa.org	Its broad mission is to develop and promote professional, educational, cultural, and humanitarian charitable efforts for the community nationally and for the international community especially our country of origin, Iraq.<br><br>IMSA has evolved into a vibrant and dynamic community of health professionals whose driving vision has been the promotion of harmony and unity in the context of scientific and cultural enrichment while strictly adhering to a policy of non-discrimination, whether ethnic, national, or religious.	NA	26th-29th May 2023<br>USA Convention<br>This convention is designed to provide physicians with up-to-date, evidence-based information on commonly encountered issues in different specialties, while suggesting pragmatic approaches to clinical management. The discussion-based format of the conference will encourage audience participation through dynamic lectures and case-based studies. Keynote speaker will talk about anthropology of Medicine. The successful completion of this interactive program diagnosing, treating, and prescribing the most effective courses of treatment, with the goal of improving patient outcomes.<br>Target Audience<br>All Physicians and other Healthcare Professionals seeking clinical information about issues in different clinical setting.<br>Speakers<br>Our presenting national faculty speakers make it their professional goals to enhance the training of clinicians.<br><br>28th May 2022<br>Anual Convention<br>CME Activity designed to examine current and emerging treatments in different diseases across different disciplines<br>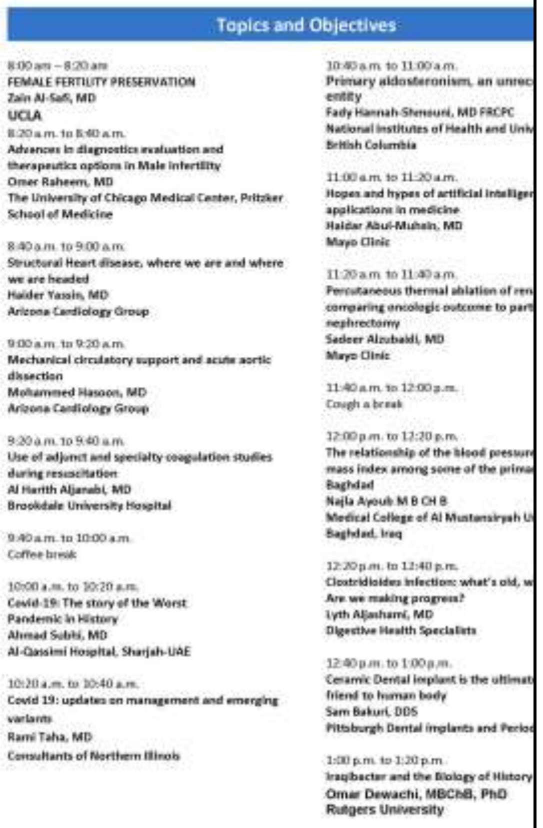<br>Topics and Objectives<br>8:00 am – 8:20 am<br>FEMALE FERTILITY PRESERVATION<br>Zain Al-Safi, MD<br>UCLA<br>8:20 a.m. to 8:40 a.m.<br>Advances in diagnostics evaluation and therapeutics options in Male infertility<br>Omer Raheem, MD<br>The University of Chicago Medical Center, Pritzker School of Medicine<br>8:40 a.m. to 9:00 a.m.<br>Structural Heart disease, where we are and where we are headed<br>Haider Yassin, MD<br>Arizona Cardiology Group<br>9:00 a.m. to 9:20 a.m.<br>Mechanical circulatory support and acute aortic dissection<br>Mohammed Hasoon, MD<br>Arizona Cardiology Group<br>9:20 a.m. to 9:40 a.m.<br>Use of adjunct and specialty coagulation studies during resuscitation<br>Al Harith Aljanabi, MD<br>Brookdale University Hospital<br>9:40 a.m. to 10:00 a.m.<br>Coffee break<br>10:00 a.m. to 10:20 a.m.<br>Covid-19: The story of the Worst Pandemic in History<br>Ahmad Subhi, MD<br>Al-Qassimi Hospital, Sharjah-UAE<br>10:20 a.m. to 10:40 a.m.<br>Covid 19: updates on management and emerging variants<br>Rami Taha, MD<br>Consultants of Northern Illinois<br>10:40 a.m. to 11:00 a.m.<br>Primary aldosteronism, an unrec<br>entity<br>Fady Hannah-Shmouni, MD FRCPC<br>National Institutes of Health and Univ<br>British Columbia<br>11:00 a.m. to 11:20 a.m.<br>Hopes and hypes of artificial intelliger<br>applications in medicine<br>Haidar Abul-Muhsin, MD<br>Mayo Clinic<br>11:20 a.m. to 11:40 a.m.<br>Percutaneous thermal ablation of ren<br>comparing oncologic outcome to part<br>nephrectomy<br>Sadeer Alzubaidi, MD<br>Mayo Clinic<br>11:40 a.m. to 12:00 p.m.<br>Cough a break<br>12:00 p.m. to 12:20 p.m.<br>The relationship of the blood pressure<br>mass index among some of the prima<br>Baghdad<br>Najla Ayoub M B CH B<br>Medical College of Al Mustansiryah U<br>Baghdad, Iraq<br>12:20 p.m. to 12:40 p.m.<br>Clostridioides infection: what's old, w<br>Are we making progress?<br>Lyth Aljashami, MD<br>Digestive Health Specialists<br>12:40 p.m. to 1:00 p.m.<br>Ceramic Dental implant is the ultimat<br>friend to human body<br>Sam Bakuri, DDS<br>Pittsburgh Dental implants and Period<br>1:00 p.m. to 1:20 p.m.<br>Iraqibacter and the Biology of History<br>Omar Dewachi, MBChB, PhD<br>Rutgers University	NA
Medical Associations of Nigerians Across Great Britain	http://www.mansag.org	This association was formed in 1997 following a long period of cross fertilisation of ideas amongst Nigerian doctors resident in the United Kingdom. It is one of the main bodies representing Nigerian Medical doctors and allied	19th Nov. 2022<br>Curious about what different specialties do day to day?<br>Find out on Sat 19th Nov 0900 at our careers fair for medical student and non-specialty doctors.	10th June 2023<br>This would be a virtual meet Leadership Academy for doctors.	6th May 2023<br>Stay uptodate with an overview of Fitness to Practice, Brain Drain in Africa, the UK-IMG experience and top tips on excelling in medical job interviews.

<table>
<tr>
<td></td>
<td></td>
<td>professionals in the British Isles.

It aims to:
To promote the professional wellbeing and social integration of doctors and allied health professionals of Nigerian heritage living in the United Kingdom;

To support successful integration of new entrants into the nation;

To provide opportunities for academic advancement for members;

To facilitate recreational and social activities;

To carry out charitable activities aimed at supporting important causes within the UK and Nigeria.
Mission</td>
<td>
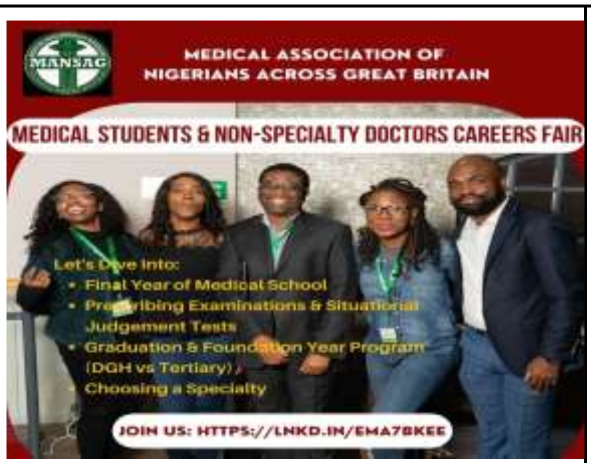

7th May 2022
. Join MANSAG Manchester Region for the Spring Educational Meeting.
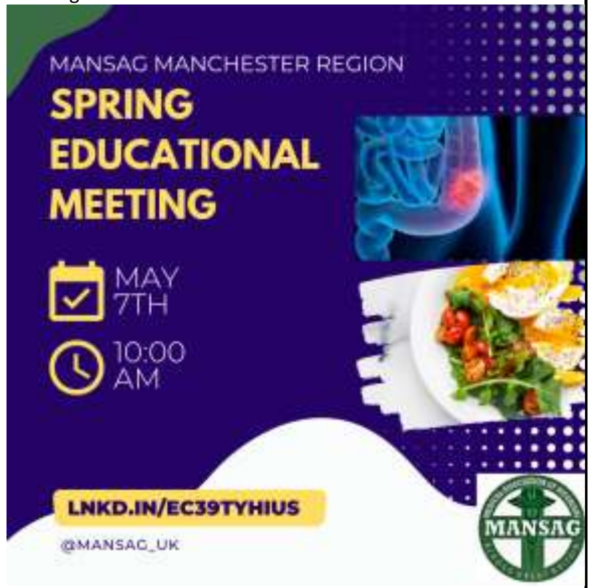

27th Nov. 2021
We are pleased to announce our partnership with American University of Antigua College of Medicine @auamed for a #scholarship scheme. This opportunity is open to Nigerian #students aspiring to study Medicine.
</td>
<td>
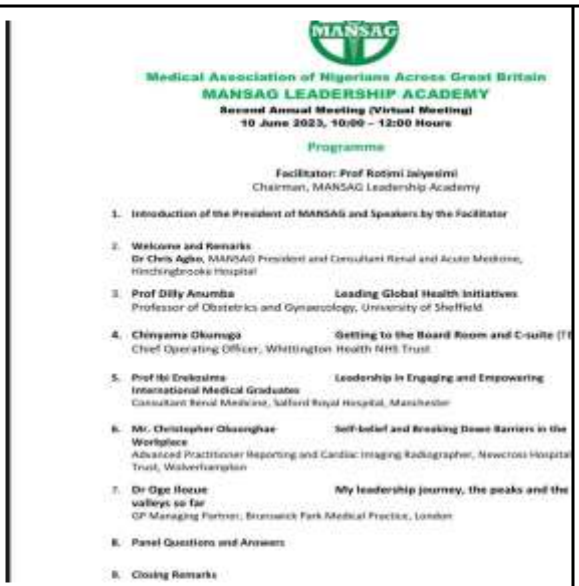
MANSAG
Medical Association of Nigerians Across Great Britain
MANSAG LEADERSHIP ACADEMY
Second Annual Meeting (Virtual Meeting)
10 June 2023, 10:00 – 12:00 Hours
Programme
Facilitator: Prof Rotimi Jaiyesimi
Chairman, MANSAG Leadership Academy
1. Introduction of the President of MANSAG and Speakers by the Facilitator
2. Welcome and Remarks
Dr Chris Agbo, MANSAG President and Consultant Renal and Acute Medicine, Hinchingbrooke Hospital
3. Prof Dilly Anumba — Leading Global Health Initiatives
Professor of Obstetrics and Gynaecology, University of Sheffield
4. Chinyama Okunuga — Getting to the Board Room and C-suite [illegible]
Chief Operating Officer, Whittington Health NHS Trust
5. Prof Ibi Erekosima — Leadership in Engaging and Empowering International Medical Graduates
Consultant Renal Medicine, Salford Royal Hospital, Manchester
6. Mr. Christopher Olusanghae — Self-belief and Breaking Down Barriers in the Workplace
Advanced Practitioner Reporting and Cardiac Imaging Radiographer, Newcross Hospital Trust, Wolverhampton
7. Dr Oge Ilozue — My leadership journey, the peaks and the valleys so far
GP Managing Partner, Brunswick Park Medical Practice, London
8. Panel Questions and Answers
9. Closing Remarks
14th May 2023- MANSAG invites you to her annual scientific & educational symposium. Venue: Lincolnshire
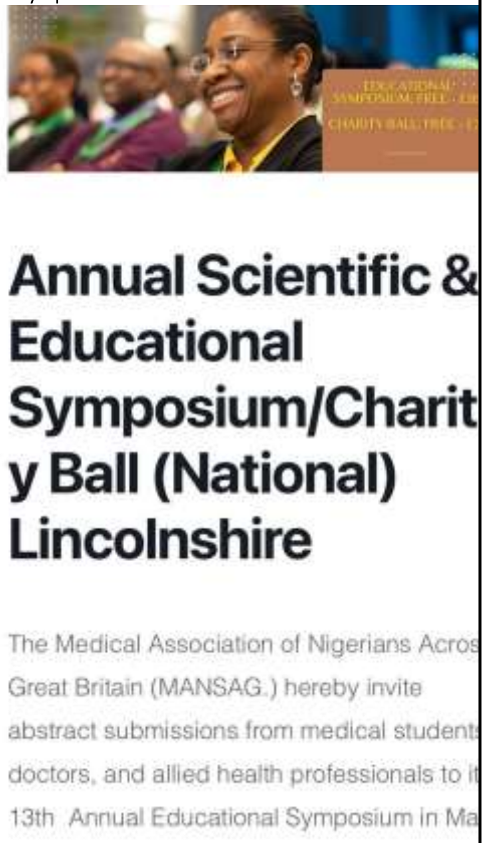

19th March 2022
Join us 19th March for MANSAG Leadership Academy
</td>
<td>
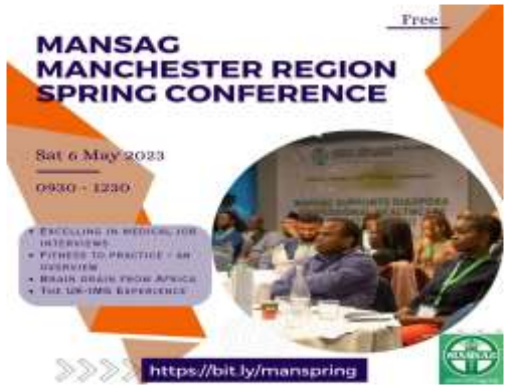

29th January 2023
Join us tonight for our New Doctors to the UK and the NHS induction.

Tips on professional conduct, communication challenges, and live Q&A to address concerns or areas of difficulty.
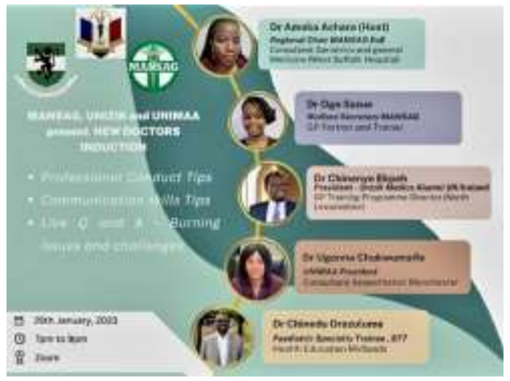

18th March 2022
Exploring the opportunity for Nigerian Medical Students in Ukraine to continue their studies in Antigua. Join in tomorrow at 5pm.
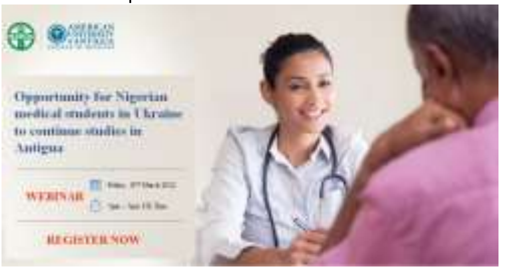

</td>
</tr>
</table>

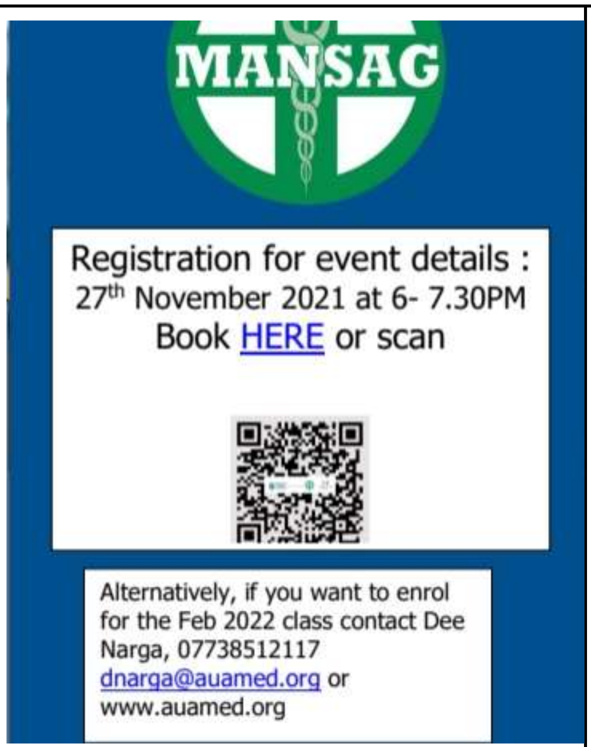

20th Nov. 2021
Career's day loading... Want to know more about different medical specialties? Join us for "How I Got Into" conversations covering Paediatrics and Neonatology, Dermatology, Plastic Surgery, Orthopaedics, Urology, Anaesthesiology, Stroke, Geriatrics, Research, General Practice, Gastroenterology and Obstetrics & Gynaecology

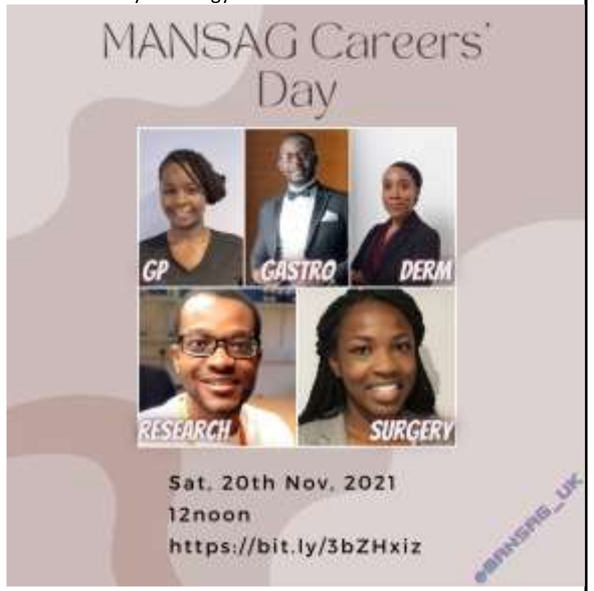

May 15th 2021
Annual Education Symposium!"A healthy transition from comfort to growth"

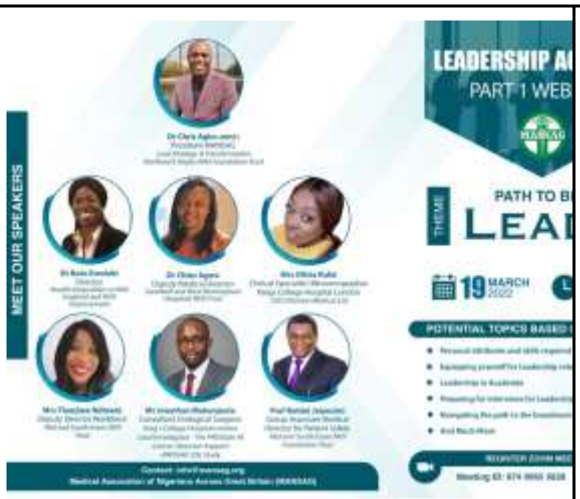

February 2022
MANSAG Travel Fellowship Award is here.

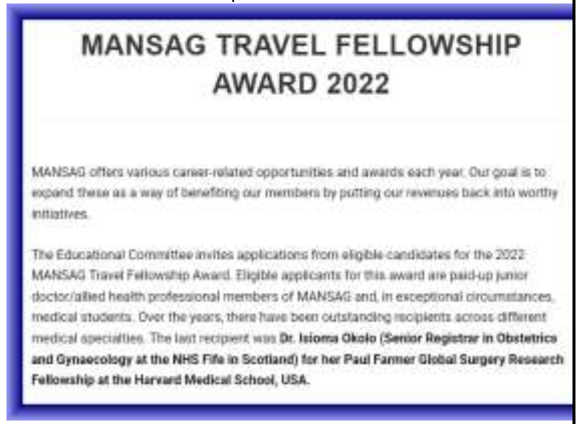

MANSAG TRAVEL FELLOWSHIP AWARD 2022

MANSAG offers various career-related opportunities and awards each year. Our goal is to expand these as a way of benefiting our members by putting our revenues back into worthy initiatives.

The Educational Committee invites applications from eligible candidates for the 2022 MANSAG Travel Fellowship Award. Eligible applicants for this award are paid-up junior doctor/allied health professional members of MANSAG and, in exceptional circumstances, medical students. Over the years, there have been outstanding recipients across different medical specialties. The last recipient was **Dr. Isioma Okolo (Senior Registrar in Obstetrics and Gynaecology at the NHS Fife in Scotland) for her Paul Farmer Global Surgery Research Fellowship at the Harvard Medical School, USA.**

9th Oct.2021
2021 MANSAG Scientific and Annual General Meeting.

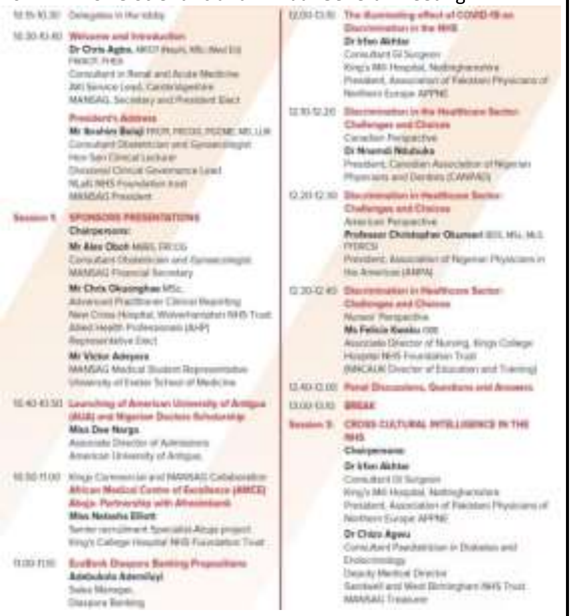

22nd May 2021
Together Webinar Series.

Theme: Professional Adaptation and Acculturation for Healthcare Professionals in the UK.

Medical Association of Nigerians Across Great Britain

11th MANSAG *(Virtual)* Annual Educational Symposium

Date: May 15, 2021

Theme: A healthy transition - From Comfort to Growth

1230 – 1235	Welcome Address by LOC chair
	**Dr. Olamide Oyende**
1235 - 1240	House keeping
	Dr. Kingsley Enohumah, Chairman Education Committee
1240 – 1245	MANSAG President's address
	Mr Ibrahim Bolaji, FRCOG
1245-1300	MANSAG Medical student Forum: Medical student mentorship programme
	Speaker: Mr Victor Adeyeru
SESSION 1:	**Developing Clinical Leaders in Postgraduate Medical Training**
	Moderator: Mr Ibrahim Bolaji
1300 – 1320	Postgraduate medical training: optimising the opportunities
	Speaker: Dr Georgette Oni
1320 – 1340	Medical legal pitfalls: How to navigate the terrain
	Speaker: Dr Victor Ameh
1340 – 1400	Our Health: Climb the Ladder
	Speaker: Dr. Sandra Isibor
1400 - 1415	Q & A
1415-1420	Break

1st May 2021-Medical Research and Grants Breakout session now live with Kelechi Njoku and Christopher Osuafor

joint hybrid (live and zoom) meeting

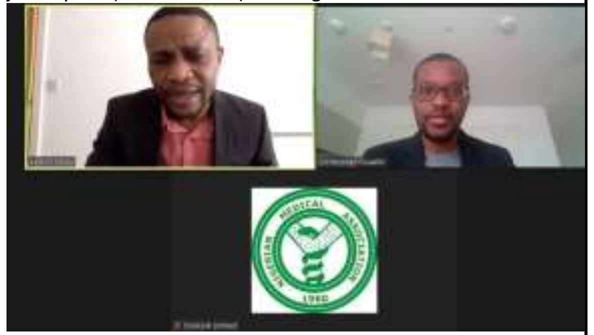

27th Feb. 2021
2021 mentorship inauguration meeting

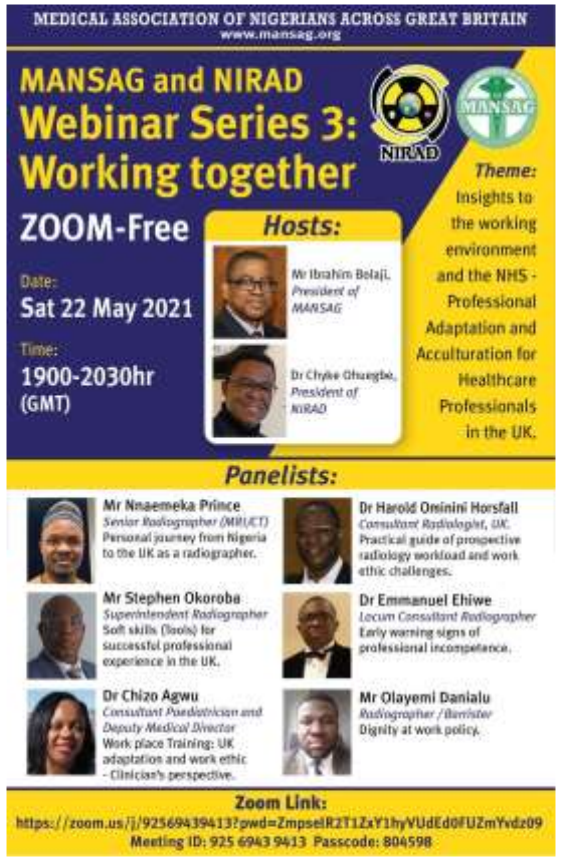

28th March 2021
Meeting the challenges of differential trainee attainment in postgraduate medical training? Save the date for an E-portfolio focused workshop by the University of Nigeria Medical Alumni UNIMA UK.

			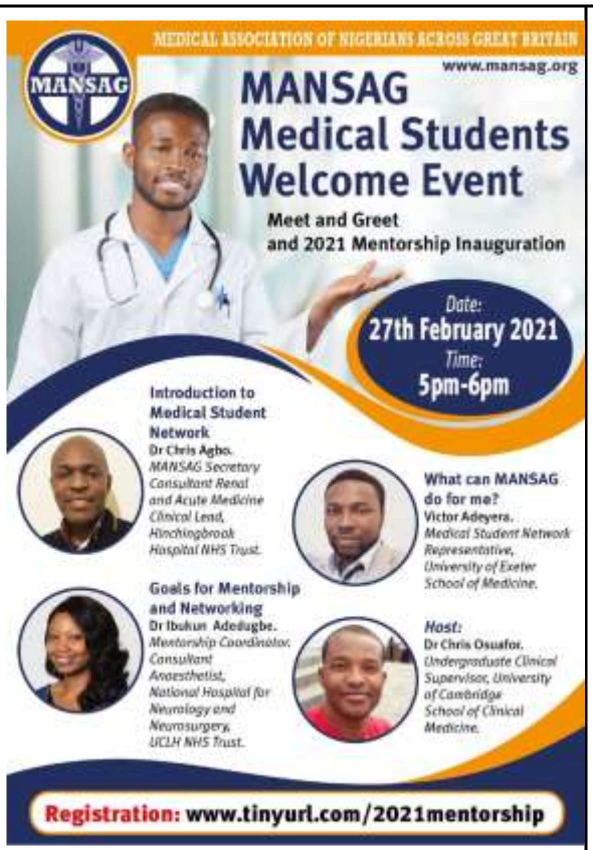	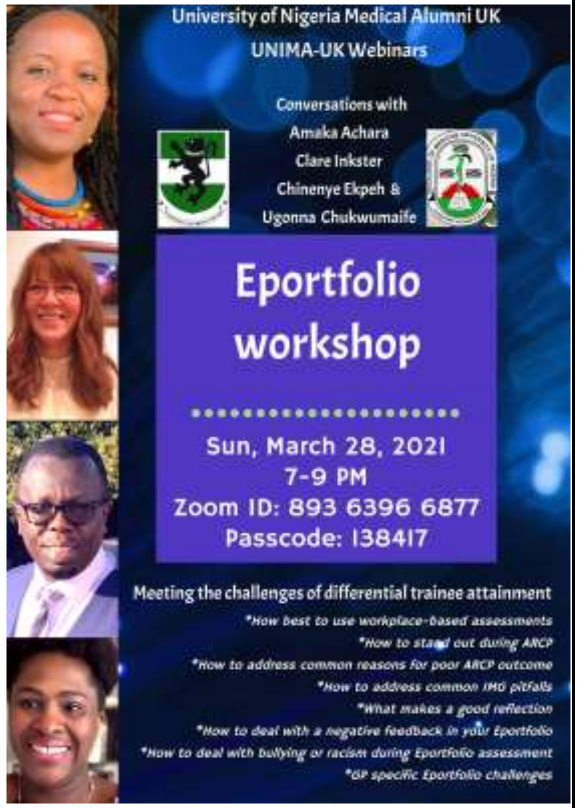<br><br>16th Jan 2021<br>Covid 19 Vaccine Panel Discussion<br><br>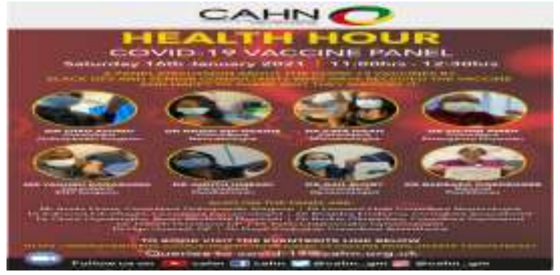	
Myanmar American Medical Education Society	http://mamesociety.org	● To benefit humanity in Myanmar Community<br><br>● To promote the culture and the exchange of scientific information in Myanmar Medical Community<br><br>● To provide the information and support for the Medical Education of Myanmar Physicians	NA	July 23, 2023, Sunday<br><br>7 AM - 5 PM<br><br>17th Annual Scientific Meeting<br>Theme: OVERVIEW OF COMMOM MEDICAL PROBLEMS and CURRENT MANAGEMENT<br><br>Program Agenda<br><br>7 - 7:40 AM Registration and Breakfast<br>7:40 - 7:50 Welcome, Dr. Alfred Leong, President, MAMES<br>7:50- 8 AM Overview, Dr. Yin Phyu Lwin, Education Committee Chair, MAMES<br><br>Morning Session - Moderator - Dr. Thida Aye<br>8:00 - 8:30<br>Update in COVID-19 infection and management<br>Dr. Zeyar Thet, MD, FIDSA	September 17, 2023<br><br>Topic: Residency Mock Interview<br><br>August 20, 2023<br><br>Time: 9 AM to 11 AM<br><br>Zoom Online<br><br>14, May 2023, Sunday<br><br>8AM to 1 PM<br><br>Research and Career Guidance Seminars

				Program Director/ Vice Chair, Department of Medicine, Wyckoff Height Medical Center<br>8:30 - 8:40 Q & A<br><br>8:40 - 9:10<br>Chronic Liver Diseases and Indications for liver transplant<br>Dr. Alvin Htut<br>Gastroenterologist & Transplant Hepatologist Assistant Professor<br>Donald and Barbara Zucker School of Medicine at Hofstra/Northwell<br>9:10 - 9:20 Q & A<br><br>9:20 - 9:50<br>Immunotherapy in Cancer Treatment<br>Dr. Morana Vojnic, MD, MBA<br>Director, Medical Oncology of Brain & Spine metastasis program Lenox Hill Hospital<br>Northwell Health Cancer Institute at MEETH<br>Assistant Professor of Medicine, Donald and Barbara Zucker School of Medicine at Hofstra/Northwell<br>9:50 -10:00 Q & A<br><br>10:00 -10:10 Break<br><br>10:10-10:40<br>Common Thyroid Disorders in Primary Care setting<br>Dr. Kyaw Kyaw Soe<br>Associate Professor at UT Southwestern Medical Center, Endocrinologist, Dallas VA Medical Center, Dallas Texas 10:40-10:50 Q & A<br><br>10:50 -11:20<br>Medical & Surgical Management of Valvular Heart diseases<br>Dr. Nino Mihatov Interventional and structural cardiologist, NYP Brooklyn Methodist Hospital<br>Assistant Professor of Medicine at Weill Cornell Medical College<br>11:20 -11:30 Q & A<br><br>11:30 - 1:00 Lunch, Exhibit and Posters<br><br>Afternoon Session - Moderator – Dr. Yin Phyu Lwin<br><br>1:00 – 1:30<br>Approach to Inflammatory Arthritis<br>Dr. Kyawt Shwin Assistant Professor, Department of Medicine, University of Texas Southwestern<br>1:30 – 1:40 Q & A<br><br>1:40 - 2:10<br>Obesity induced Cardiovascular Dysfunction<br>Dr. Stephen Peterson, MD, MACP<br>Chair, Department of Medicine, NYP-BMH, Professor of Medicine, Weill Cornell Medical College<br>2:10 - 2:20 Q & A<br><br>2:20 - 2:50<br>Evaluation and Management of Pulmonary Nodule<br>Dr. Ivan Wong Assistant Professor of Clinical Medicine, NYP	8:00 - 8:30 am - Registration and Breakfast (Ball rooms, Phoenix East)<br><br>8:30 - 8:40 am -Introduction/President speech<br><br>Research seminar<br><br>8:40 - 9:20 am<br><br>Topic: Successful Research in Residency, A goal within reach<br><br>Speaker: Evan Siau, MD<br><br>Assistant Professor, Icahn School of Medicine at Mount Sinai<br><br>9:20 -10:00 am<br><br>Topic: Introduction to Clinical Research<br><br>Speaker: Jessie Van Daele, PhD.<br><br>Director of Clinical Research at The Brooklyn Hospital Center. New York.<br><br>Q & A<br><br>10:00 - 10:15 am – Coffee Break (Ball rooms, Phoenix East)<br><br>Career Planning & Guidance Seminar<br><br>10:15 -10:25 am<br><br>Topic: What are an IPA and an ACO? Introducing CAIPA & CAIPA Care<br><br>Speaker- George Liu, MD, PhD, Chair, President & CEO of the Coalition of Asian-American IPA (CAIPA)<br><br>10:25 -10:45 am<br><br>Topic: The Financial Impact to an Independent Practitioner who Participates in a Successful IPA/ACO<br><br>Speaker-Jing Shui, Chief Strategy Officer, CAIPA MSO<br><br>10:45 -11:05 am<br><br>Topic: Population Health Management in a Value-Based Contracts Environment<br><br>Speaker- Eliza Ng, Chief Medical Officer, CAIPA MSO<br><br>Q & A<br><br>11:10 -11:30 am<br><br>Topic: Hospitalist position<br><br>Speaker-Thida Aye, MD, MPH<br><br>Assistant Professor, Donald and Barbara School of Medicine at Hofstra/Northwell.

				Brooklyn Methodist Hospital<br>2:50 - 3:00 Q & A<br><br>3:00 - 3:10 Break<br><br>3:10 - 3:40<br>Clinical Approach to Patients with Dizziness<br>Dr. Kenneth Y. Chao<br>Assistant Professor, NYU Long Island School of Medicine, Neurologist at NYU Langone Health, Long Island<br>3:40 - 3:50 Q & A<br><br>3:50 - 4:20<br>5Ms of Geriatric care<br>Dr. Padam Neopane Vice Chair, Gift of Life New Jersey<br>Assistant Professor at Rutgers New Jersey Medical School, Department of Family Medicine<br>4:20 - 4:30 Q & A<br><br>4:30 - 4:40 Closing Speech, Vice President, Dr. Thant Zin	Co-Director, Asian Patient Initiative.<br><br>11:30 -11:50 am<br><br>Topic: Faculty position<br><br>Speaker-Alfred Ba Tun Leong, MD,FACP, CNSC.<br><br>Assistant Professor of Clinical Medicine, Weill Cornell medicine.<br><br>Co-Chief, Ambulatory Clinic, NYP Brooklyn Methodist Hospital.<br><br>Q & A<br><br>Welcome and Graduation<br><br>12:00-12:15 pm<br><br>Residency successful Tips<br><br>12:15- 12:50 PM<br><br>Advice from Residents, Fellows and Faculty members<br><br>12:50- 1:00 PM - Closing speech<br><br>9th October 2022<br><br>Time: 9:30 AM (EST)<br>Topics: Resources for IMGs, How to Ace an Interview forResidency Register IMG Seminar<br><br>August 27, 2022 (8:30 AM to 10 AM)<br>IMG Seminar 2022<br>Via Zoom<br><br>15th Aug 2021,<br>Guidance for Residency Online Seminar
Nepalese Doctors Association	http://ndauk.org.uk/index.php/home	The Nepalese Doctors Association (UK) is an organisation comprised of medical doctors who are dedicated to supporting medical education and health improvement projects for the Nepalese community in the UK and Nepal.	23rd October 2021<br>Zoom webinar on how to get to medical school and what the life is as junior doctor or specialist.	10th June 2023<br>We are pleased to announce our 2nd NDAUK Surgical Skills Course 2023 in collaboration with @hullhospitals The Suture Centre and The Herrick Society. This course has been approved for 6 CPD points by @rcsed.	3rd September 2022<br>We are pleased to announce our new International Medical Teaching Series Webinar launching this Saturday.<br>Topic: Insights for a Successful NHS Job Interview<br>Registration: scan the QR code in the flyer

			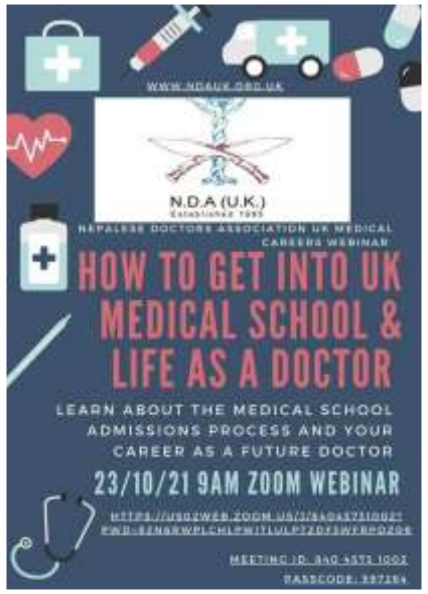<br>BBB	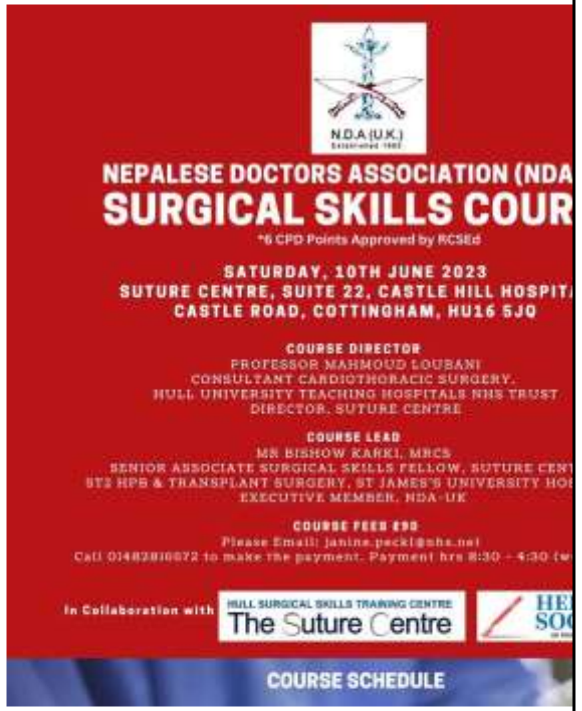<br>25th March 2023<br>NDAUK Junior Doctors Conference 2023-London:<br>We are pleased to announce that registration is open for the 5th NDAUK Junior Doctors Conference 2023 to be held in central London.<br>Date: 25th March, 2023<br>Venue: University College London (Torrington Place)<br>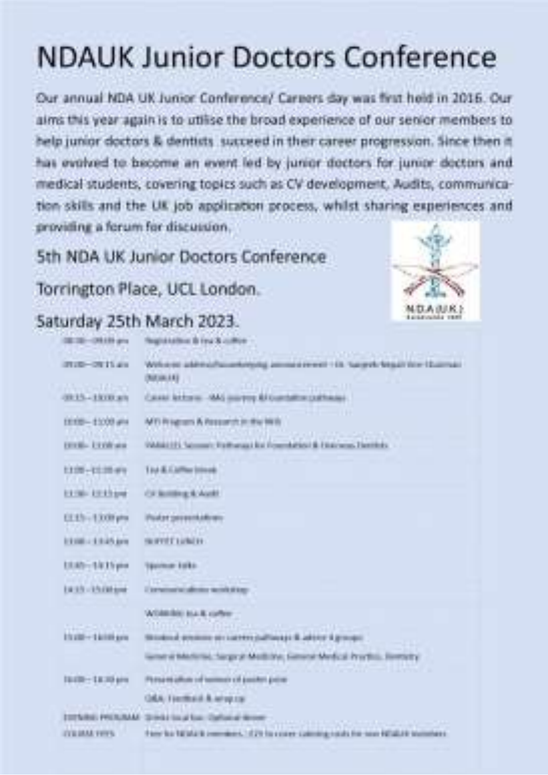<br>NDAUK Junior Doctors Conference<br>Our annual NDA UK Junior Conference/ Careers day was first held in 2016. Our aims this year again is to utilise the broad experience of our senior members to help junior doctors & dentists succeed in their career progression. Since then it has evolved to become an event led by junior doctors for junior doctors and medical students, covering topics such as CV development, Audits, communication skills and the UK job application process, whilst sharing experiences and providing a forum for discussion.<br>5th NDA UK Junior Doctors Conference<br>Torrington Place, UCL London.<br>Saturday 25th March 2023.<br><br>24th September 2022<br>Excellent opportunity for junior doctors (Locally Employed Doctors) to share experience and problem facing during career progression	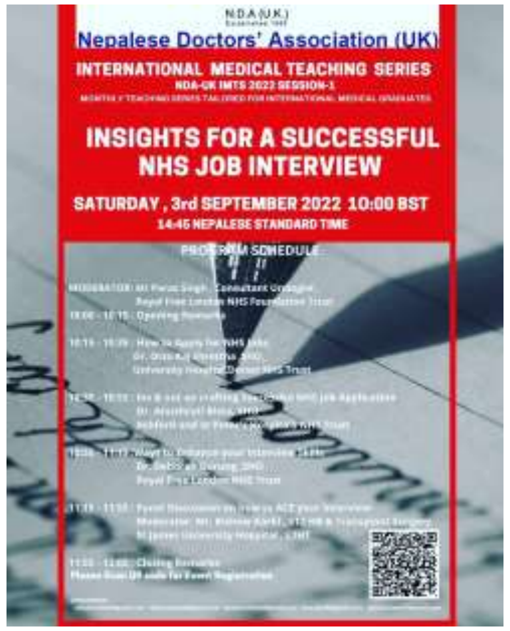<br>5th February 2022<br>NDAUK career webinar :<br>Time 10 AM BST (15:45 PM NST)<br>GP Training and Applying for Specialist Registration through Non-training grade<br>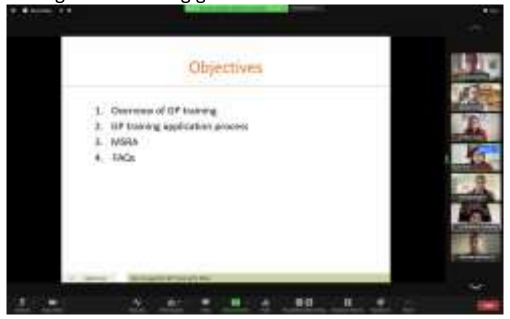

				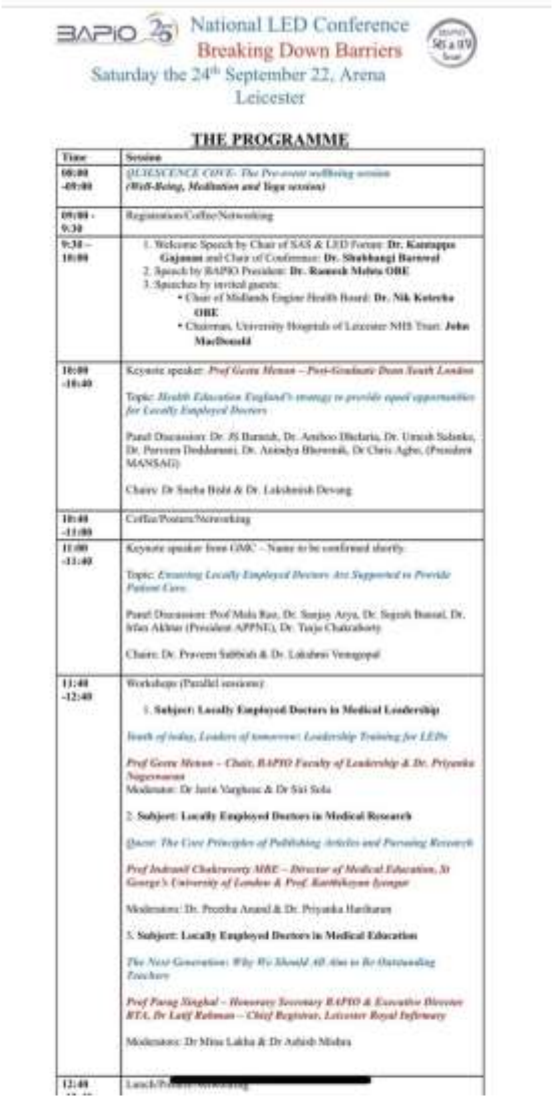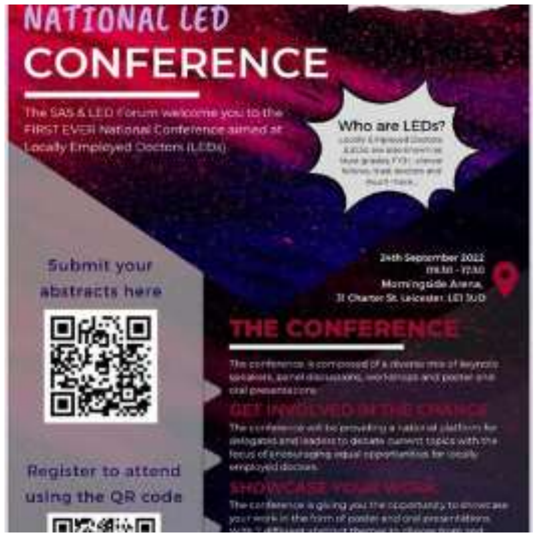<br>23rd July 2022<br>Theme:Long COVID and post COVID mental issues	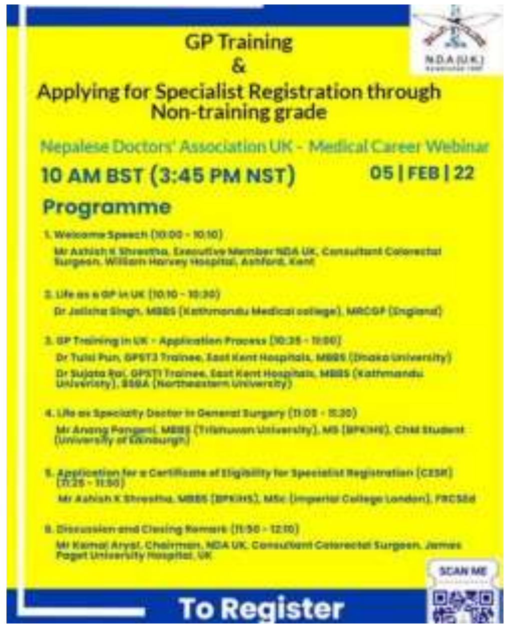<br>11th December 2021<br>PLAB and Beyond, how to get a job in the NHS<br>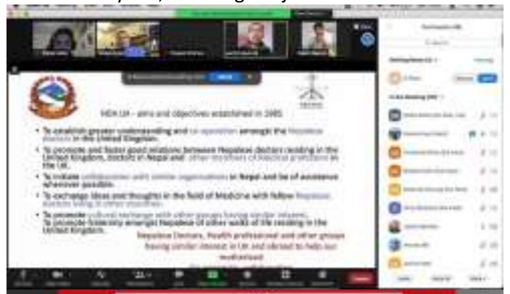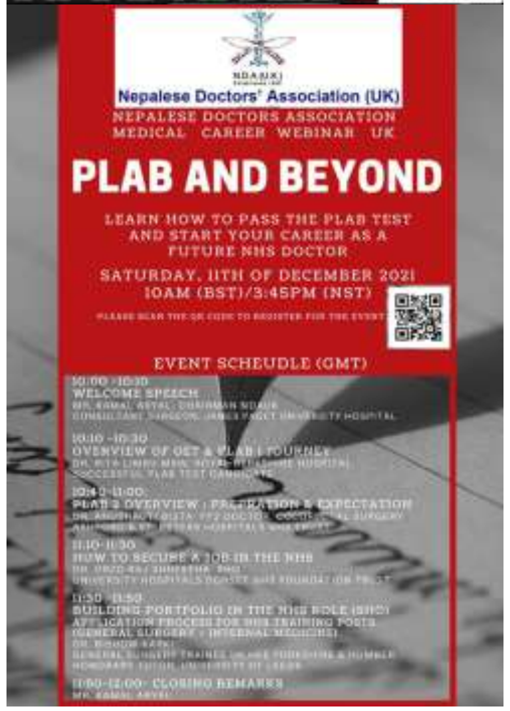<br>vvv

				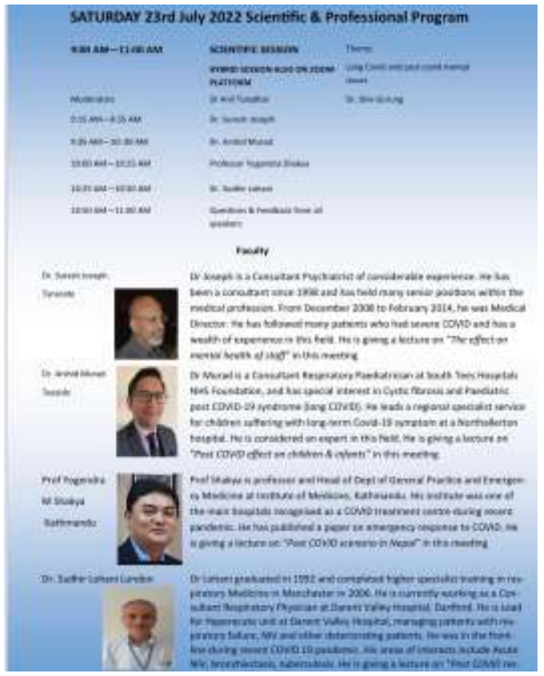<br>31st July 2021<br>36th Annual Conference<br>Scientific Session<br>Theme : Gastroenterology<br>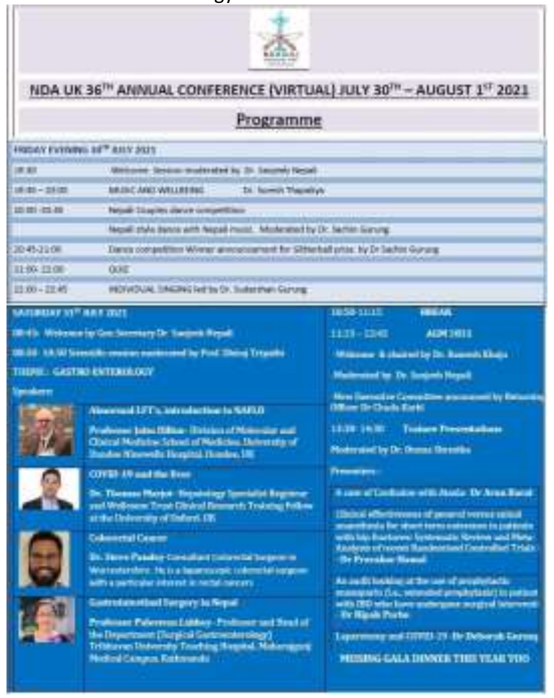	

				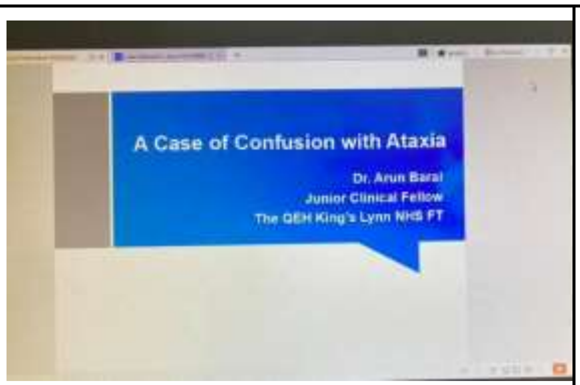	
Nicaraguan American Medical Association	http://www.nicamed.com	NA	NA	NA	NA
Nigeria American Medical Foundation International	https://namfi.org	NA	NA	NA	NA
North American Taiwanese Medical Association	https://www.natma.org	NA	NA	23rd January 2022<br>Join the NATMA Southern California Chapter's Zoom meeting this coming Sunday,<br>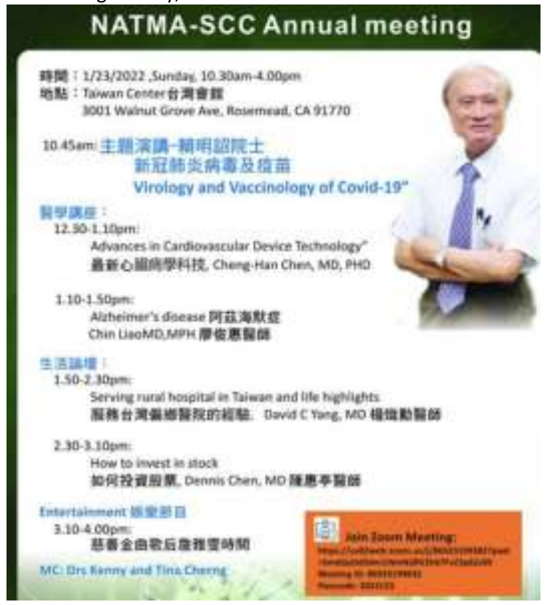	NA
Northern Indian Medical & Dental Association of Canada	http://www.nimdac.com	It is a non profit organization which is run by the office bearers who are elected by the members annually. Its purposes are to update its members with the current medical literature, with the scientific and educational programs, which are held regularly throughout the year. We organize educational, charitable, social and cultural events regularly throughout the year.	NA	NIMDAC 28th Annual CME/CDE Conference<br>Saturday Oct 15th 2022<br>• Check-in time 3pm<br>• Check-out time 12pm<br>11:00 Arrival/Registration<br>11:30 - 1:30 Lunch<br>12:00 - 12:15 CME/CDE Welcome<br>12:15 - 1:00 Quality Improvement in Practice<br>Dr. Mary Manno, CPSO<br>1:00- 1:45 The Post-pandemic 'new normal': What to know on the medico-legal front<br>Dr. Katherine Lariviere, CMPA<br>1:45-2:30 On Second Thought: Managing different populations of people with<br>Cardiometabolic Disease (Accredited Program)<br>Dr. Anil Gupta<br>2:30 - 2:45 Nutrition Break<br>2:45 - 3:30 Combining and Augmenting therapy in Anxiety and	NA

				Depression - Clinical<br>Challenges and Psychopharmacology<br>Dr. Gaurav Mehta<br>3:30 - 4:15 The Mouth-Body Connection<br>Dr. Gagan Bhalla<br>4:15 - 4:30 Mindfulness/Meditation Session<br>Mrs. Shilpa Mehta<br>4:30 - 4:45 Wrap Up<br>5:00 - 6:00 NIMDAC General Meeting<br>7:00 - 12:00am Evening Gala/Dinner	
Organization of Sierra Leonean Healthcare Professionals Abroad	http://www.toshpa.org.uk/index.htm	This is a professional and non-political think tank healthcare organization of all Sierra Leonean professionals abroad, committed to providing healthcare relief services and creating ideas and policy debates that will shape the future of healthcare policy, practice, and regulation in the country for a healthier Sierra Leone.	NA	NA	NA
Pakistan Medical Association UK	https://www.facebook.com/pages/category/Medical-Company/Pakistan-Medical-Association-UK-346946281999626	NA<br>site not available	NA	NA	NA
People To People (Ethiopia)	https://p2pbridge.org/ ….<br><br>https://p2pbridge.wordpress.com (site no longer working)	People to People (P2P) is a non-governmental, non-profit organization dedicated to improving health care and reducing the spread of diseases, particularly in Ethiopia and in diaspora communities.P2P's projects are focused on strengthening health systems through partnerships with local hospitals and universities, in which we engage the global Ethiopian diaspora in an attempt to bridge the knowledge gap and address the country's severe shortage of health and medical professionals.	NA	NA	NA
Peruvian American Medical Society	http://www.pams.org/	The Peruvian American Medical Society (PAMS) is a non-profit organization dedicated to providing quality healthcare to the underserved men, women and children of Perú with compassion, integrity, charity and respect. By working collaboratively with local providers and medical schools, PAMS is able to offer healthcare and education to thousands of patients throughout the country each year, and enhance the education of medical students and doctors in Perú.	NA<br>(Medical education related events hosted by organisation itself are mostly not advertised in English)	NA	NA
Philippine Medical Association in America	Although this representative organization has no website, there are a number of its chapters in each state.	NA	NA	NA	NA
Rajasthan Medical Alumni Association	http://www.rajmaai.com	Rajasthan Medical Alumni Association (RAJMAAI), established in 1987, is a body of medical professionals all over the country who have graduated from any of the medical institutions in Rajasthan	NA	NA	NA

		(or of Rajasthani Heritage) and have an interest in its culture and activities. RAJMAAI establishes fraternities and exchanges social and cultural activities locally, nationally, and internationally. Our members meet regularly every year for continuing medical education and also bring along their families to socialize and enjoy each others company.			
Romanian Medical Association of America	https://www.facebook.com/Romanian-Medical-Association-of-America-250257948368293	Preserve and promote Romanian medical practices, beliefs and ideals	NA	NA	NA
Romanian Medical Society UK	https://www.facebook.com/romanianmedicalsociety	The Romanian Medical Society in the UK is a network of Romanian medical professionals in the UK.	NA	NA	NA
Russian American Medical Association	http://www.russiandoctors.org/en	The Russian American Medical Association (RAMA) is a non-profit organization of Russian American physicians founded in 2002 with a mission to facilitate and enable Russian American physicians and health care professionals to excel in patient care, teaching and research, and to pursue their aspirations in professional, humanitarian and community affairs.	NA	NA	NA
Serbian American Medical and Dental Society	http://www.samds.org<br><br>Added:<br>https://samds.us/	Aim: To bring together all physicians, dentists and medical personnel of Serbian heritage currently living/working in the United States<br>To provide free screenings, educational programs and basic medical knowledge to our people living in the Unites States<br>To support and help medical students and residents of Serbian heritage interested in pursuing education/residency careers in the United States. To continue with fundraising opportunities and support medical missions and hospitals overseas.<br>To establish a yearly academic meeting for members and expand learning opportunities	NA	NA	18TH September 2022<br>Join us on a Zoom meeting<br>Topic: Residency program in the USA<br>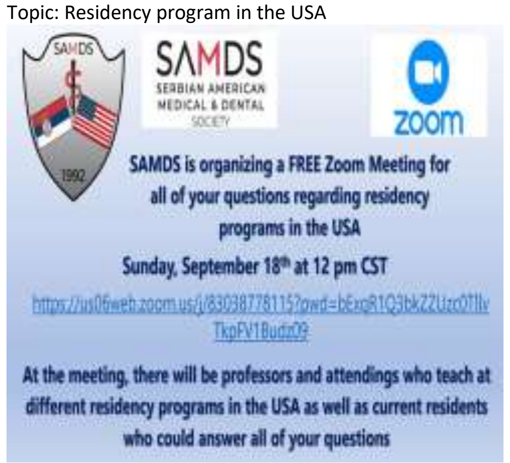
Serbian American Medical Association	http://www.serbianama.org	Aim:<br>.To Unite physicians of Serbian heritage in America and Serbia.<br><br>.Foster medical education of medical students and physicians of Serbian heritage.	31st August 2023<br>The Student Group Mentorship panel event is an opportunity to chat with medical students and residents and to learn more about the application process at each step of your medical career.	10th November 2022<br>SAMA journal club	NA

<table>
<tr>
<td></td>
<td></td>
<td>Improve healthcare conditions in Serbia and the Balkans.</td>
<td>
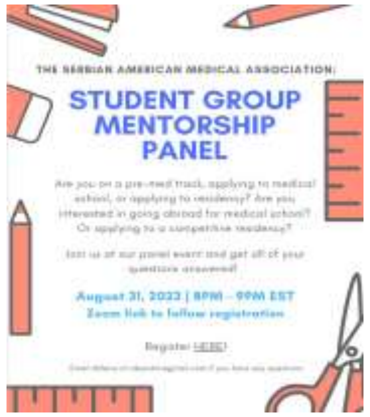

4th April 2023
We are inviting students, medical doctors or any one else interested to learn more about this pressing issue in health care to join us on a Zoom presentation by Dr. Suzana Radulovich on Tuesday April 4, 2023 starting at 8PM EST.
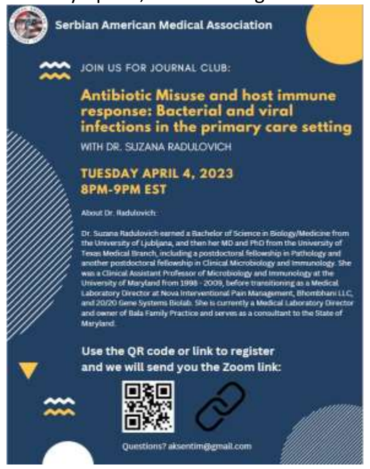

24th January 2023
Student group Journal Club
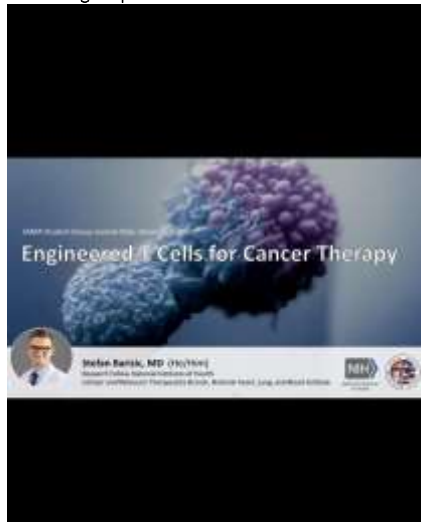

</td>
<td>
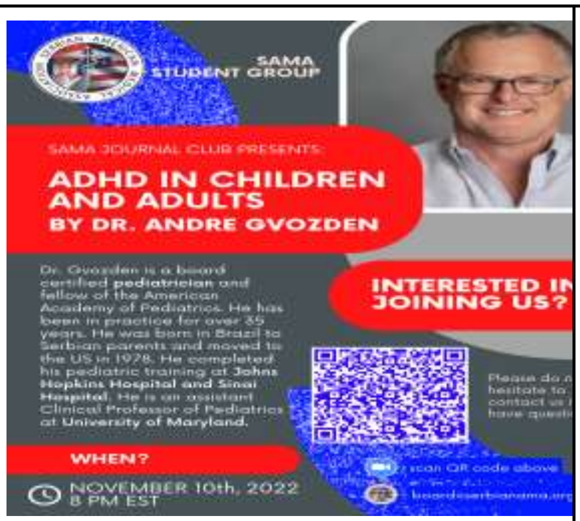

4th April 2023
We are inviting students, medical doctors or any one else interested to learn more about this pressing issue in health care to join us on a Zoom presentation by Dr. Suzana Radulovich on Tuesday April 4, 2023 starting at 8PM EST.
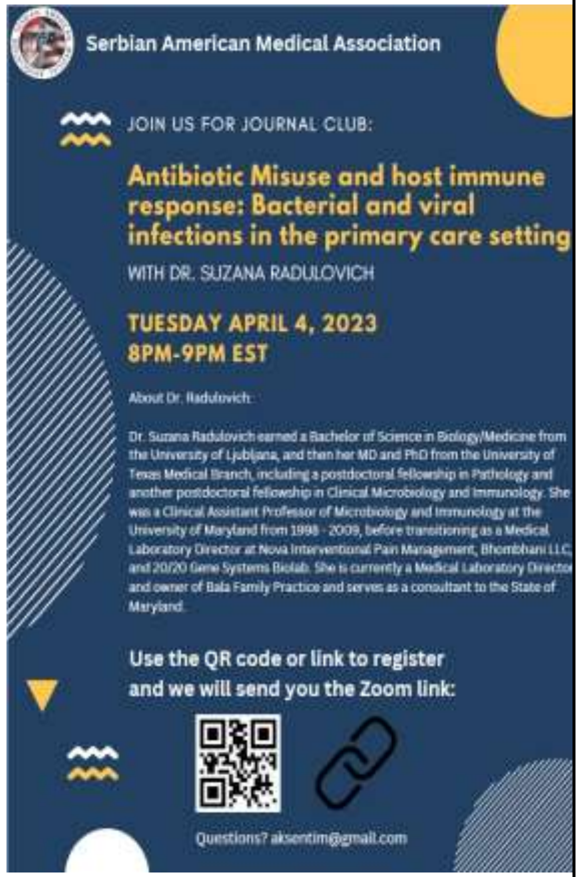

2nd March 2023
</td>
<td></td>
</tr>
</table>

				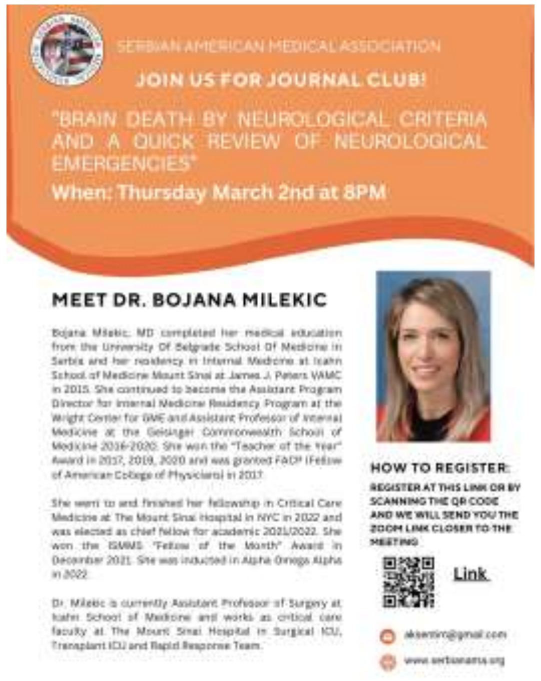	
Society of Philippine Surgeons in America	http://www.spsatoday.com	The mission and goals of the society includes, aside from it's CME program, postgraduate scholarship in the United States for graduating surgical residents in the Philippines, and humanitarian services to the underserved communities in the Philippines. All of the Presidents of the Society, in collaboration with the Board of Governors and membership, continue to maintain the prestige of this highly respected ethnic surgical organization in this country. New programs have been organized and existing programs have been strengthened.	NA	NA	NA
. South Australian Sri Lankan Doctors Association	https://sasda.org.au	This group is joined by around 25 Doctors each year who come to South Australia as postgraduate trainees from Sri Lanka or as direct migrants.	NA	NA	NA
Sri Lanka Medical Association of North America*	Although this representative organization has no website, there are a number of its chapters in each state.	NA	NA	NA	NA
Sri Lankan Medical and Dental Association of the UK	https://www.srilankan-mda.org.uk	The main aim of the SLMDA is to assist undergraduate medical & dental education in Sri Lanka. However the constitution has recently been extended to support	NA	NA	NA

		humanitarian causes of exceptional significance to Sri Lanka. (E.g. 2004 Tsunami)			
Sudanese American Medical Association	https://www.sama-sd.org	The Sudanese American Medical Association "SAMA" is a non-profit, non-political, educational and humanitarian organization. Our mission is to improve the health and wellbeing of our communities through services, capacity building, and education/research<br>Our vision is to advance medical knowledge, develop and maintain the highest professional and ethical standards of medical practice and health care through scientific, educational, and charitable initiatives.	NA	30th September 2023<br>Saturday, September 30th, 4:00 PM Sudan Time, 9:00 AM Central time<br>For registration<br>https://iecho.org/pro.../PRGM1686958637398PDPRKE5LLH/details<br>Topic: Burn and Skin Coverage<br>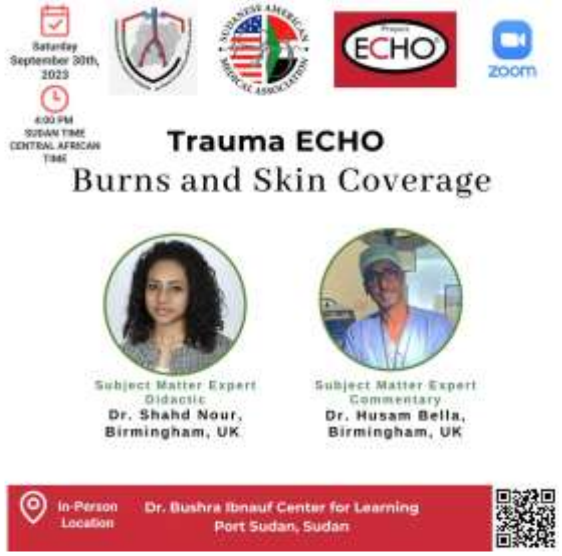<br>September 16th 2023<br>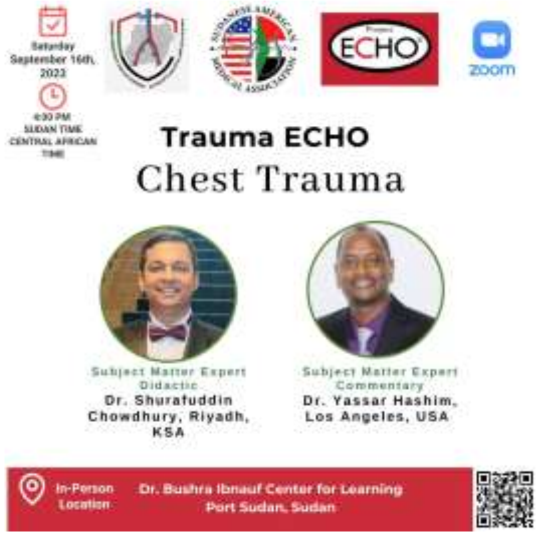<br>September 2nd 2023	26th February 2023<br>MRCP2 Review Course<br>5PM Sudan Time<br>Dr. Ihab Fathi Suliman will be answering MRCP 2 Questions.<br>All those who attend the session will have the opportunity for FREE 30 day access to Moodle Qbank.<br>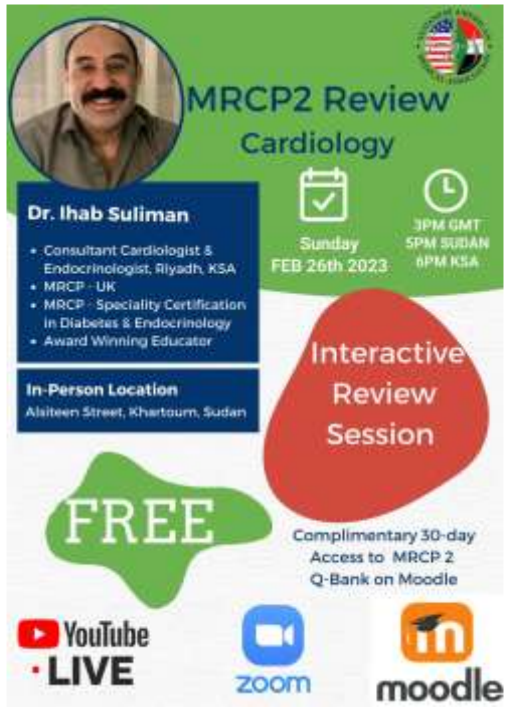<br>18th of February, 2023 4 pm, Sudan time. Are you preparing for USMLE Step 2 CK and #Match2024 ?Join us for the next meeting about exam preparation.<br>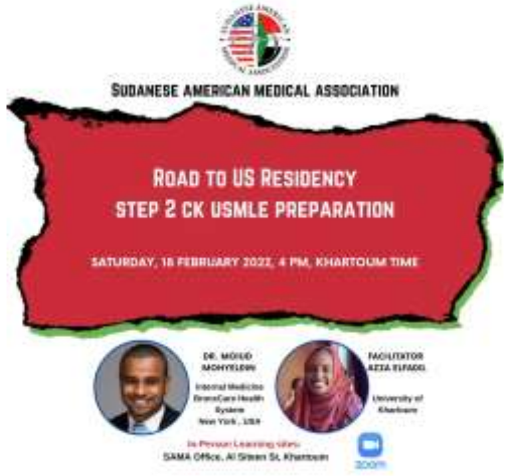<br>January 22nd 2023<br>MRCP2 Review Course

<table>
<tr><td></td><td></td><td></td><td></td><td>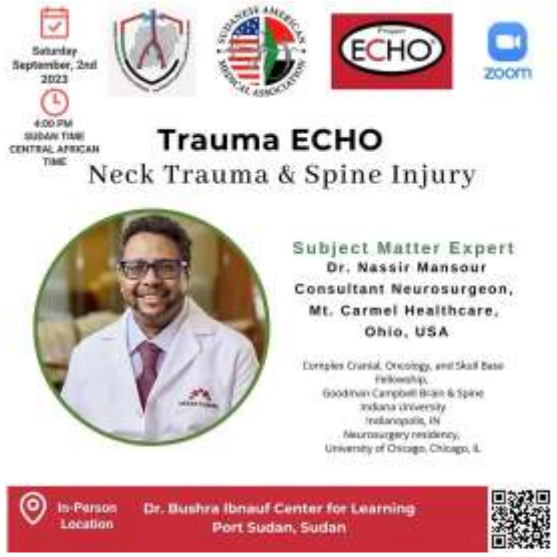

Saturday, August 19th, 4:00pm Sudan Time, 9:00 am Central Time.
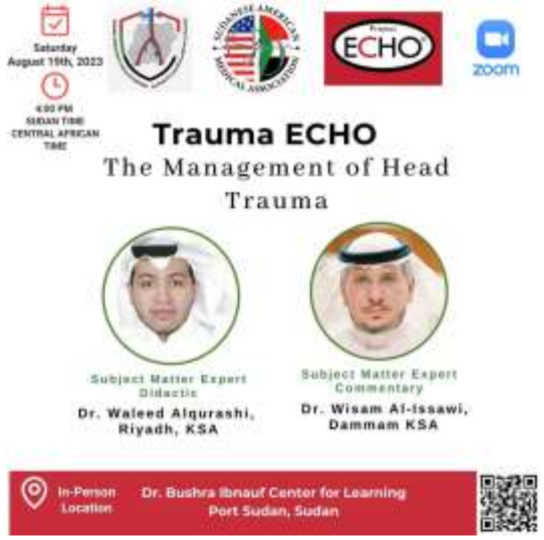

Saturday, 5th August, 4:00pm Sudan time, 9:00 am Central Time.</td><td>November 27th 2022
MRCP2 Review Course
November 6th 2022
MRCP2 Review Course
5PM Sudan Time

5th November 2022
USMLE STEP 1 PREPARATION
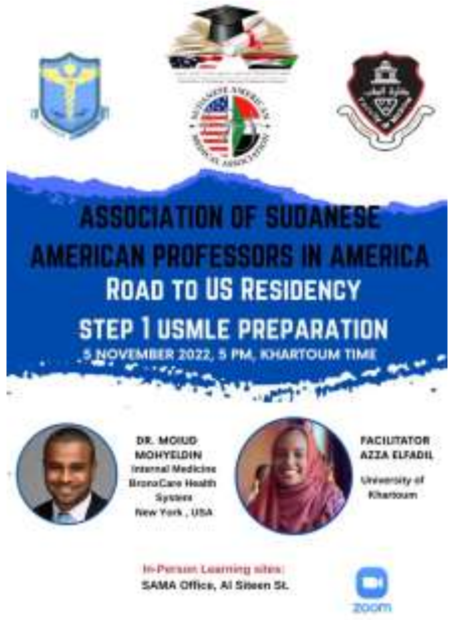

October 16th 2022
MRCP2 Review Course

15th of October, 6 pm, Sudan time.
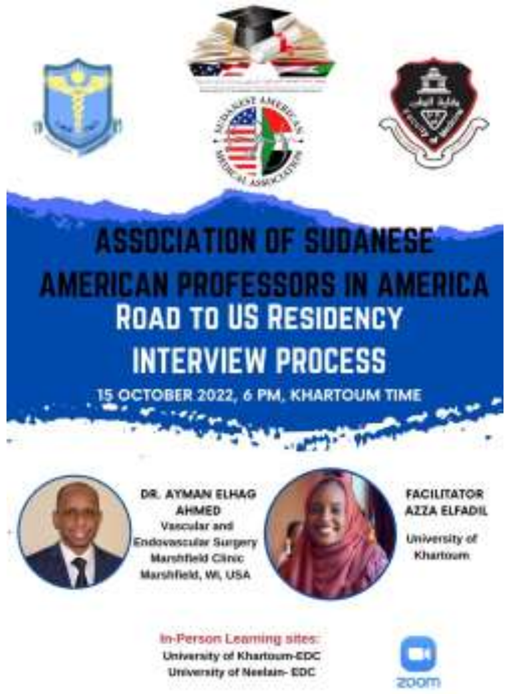
</td></tr>
</table>

<table>
<tr><td></td><td></td><td></td><td></td><td>
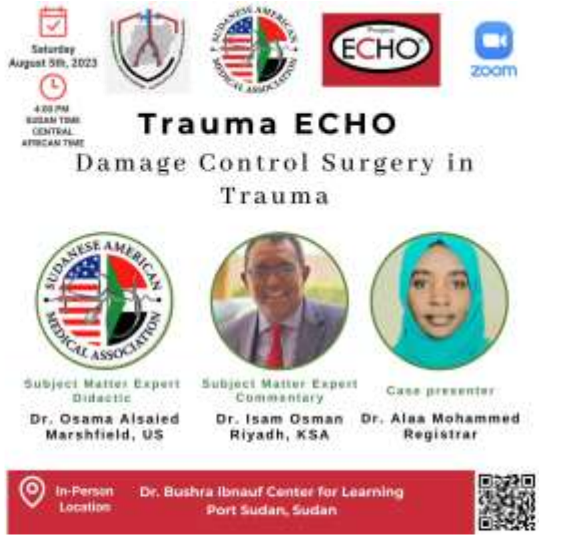

July 22nd 2023
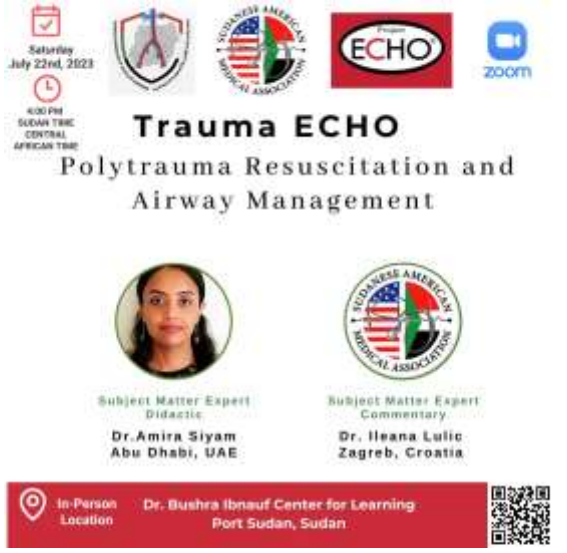

July 8th 2023
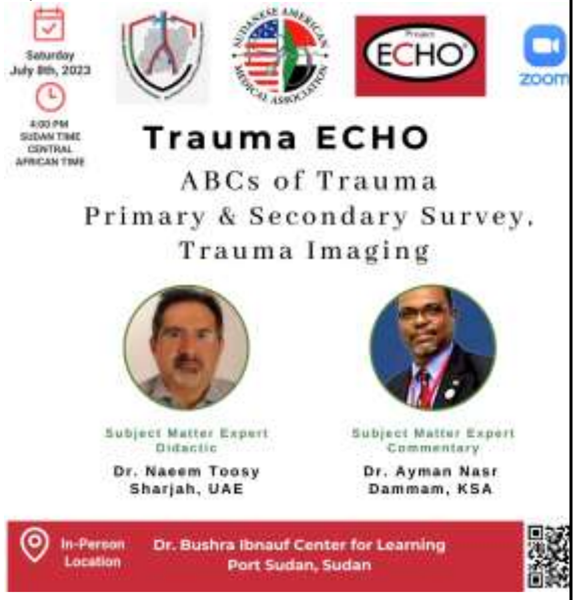

</td><td></td></tr>
</table>

				July 2nd 2023<br>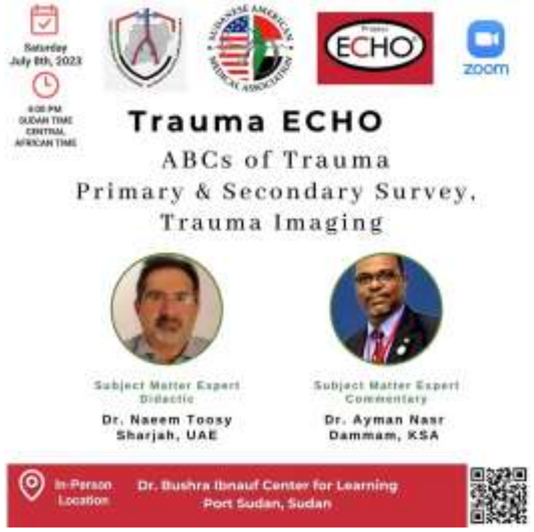<br><br>27th June 2022<br>Wound Closure & Knot Tying Techniques Training<br>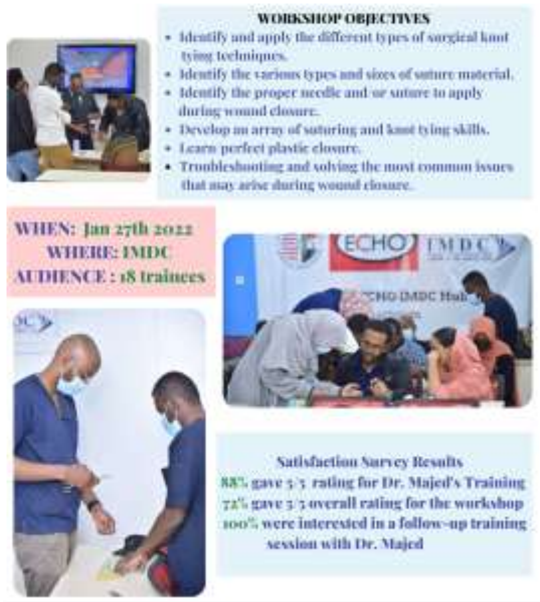<br><br>18th March 2023	

				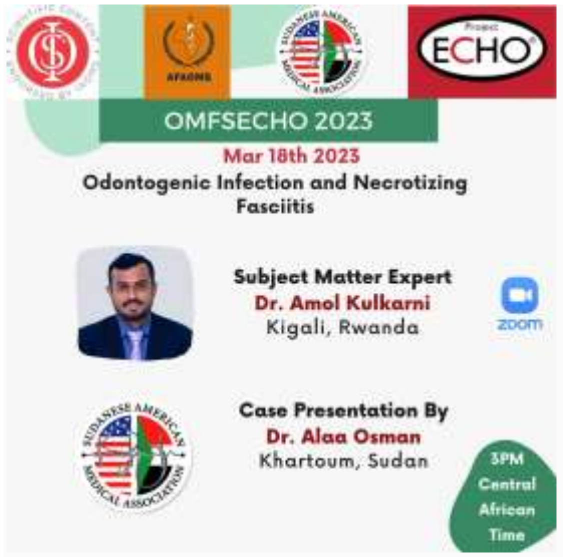<br><br>March 4th 2023<br>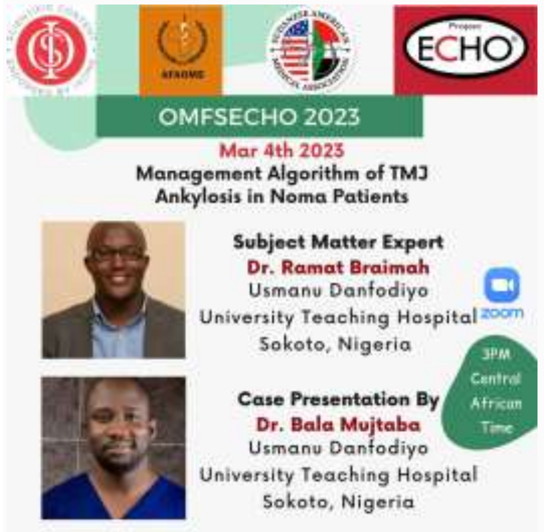<br><br>Feb 4th 2023<br>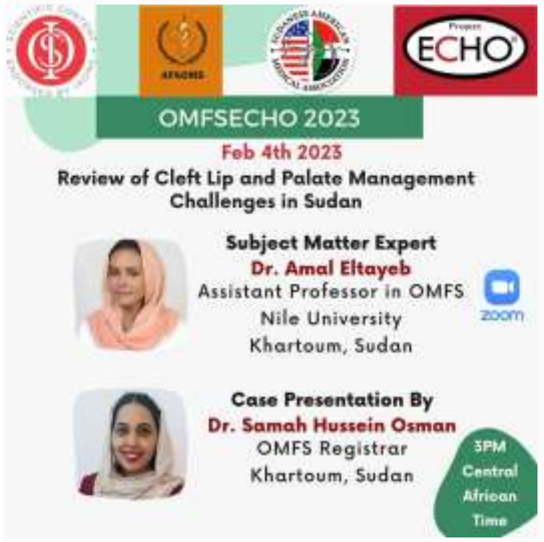<br><br>January 7th 2023	

				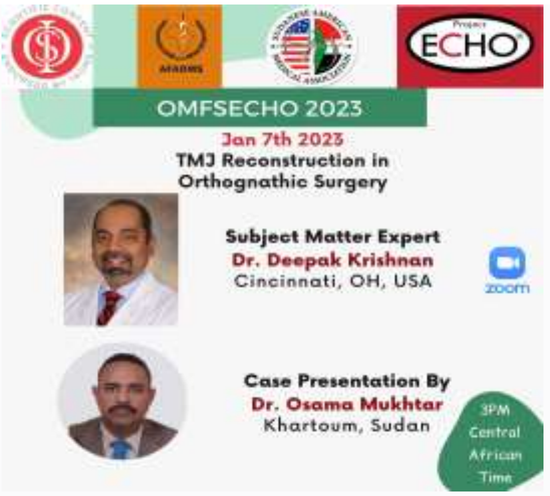<br><br>10th December 2022<br>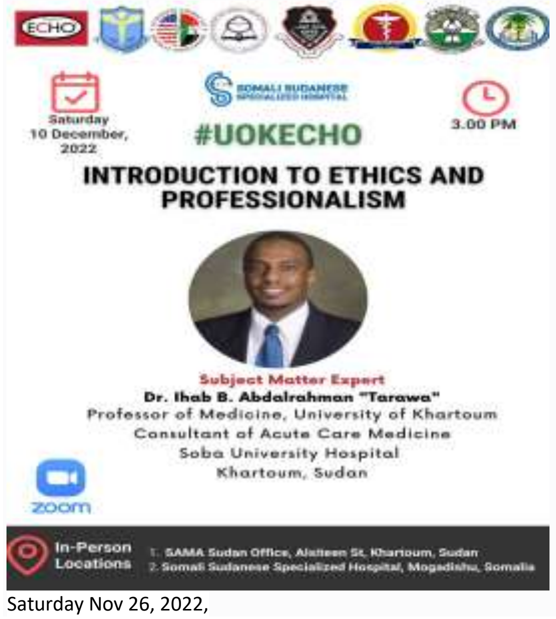<br><br>Saturday Nov 26, 2022,<br>Join us this Saturday Nov 26, 2022, in a new session of #SurgeryECHO with the topic of Neurological Emergencies<br><br>November 12th 2022 3pm Sudan Time<br>Cardiology session about STEMI, Saturday at 3 pm Khartoum time.<br><br>November 12th 2022 10:00 am Sudan time<br>the topic of "Compartment Syndrome", with the partnership of SOSA (Sudanese Orthopaedic Surgeons Association). Dr.Ali Shakir, Orthopaedics and Trauma specialist in Khartoum, Sudan, gave a didactic presentation that addressed the topic concisely. He was joined by Dr. Abbas Hassan, Orthopedics and Hand Consultant Surgeon in Omdurman teaching hospital, who aided in the discussion and shared his input and knowledge. A case was also presented by Dr.Fadwa Abdelmoniem, a medical officer in Khartoum,Sudan.<br><br>12th November 3-5pm	

				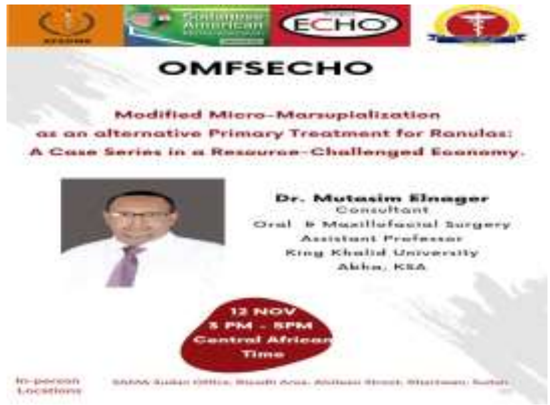<br><br>5th November 2022<br>Topic: polycistic ovary disease<br><br>29th October 2022, 10am<br>Our topic is about "Intestinal Obstruction".<br><br>29th October 2022, 3pm<br>First medical ethics session about Introduction to Communication<br><br><br>22th October 2022, 3pm<br>Gastroenterology is back with an interesting topic of Difficult ERCP!<br><br>22th October 2022, 3pm<br>Cardiovascular Complications of Immune Checkpoint Inhibitors Cardiology<br><br>15th October 2022,<br>our next session will be about appendicitis<br>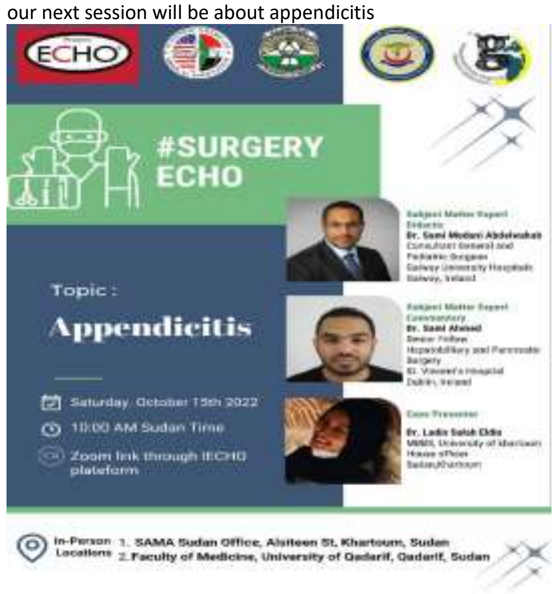<br><br>15th October 2022,<br>Mitral stenosis in pregancy	

<table>
<tr>
<td></td>
<td></td>
<td></td>
<td></td>
<td>
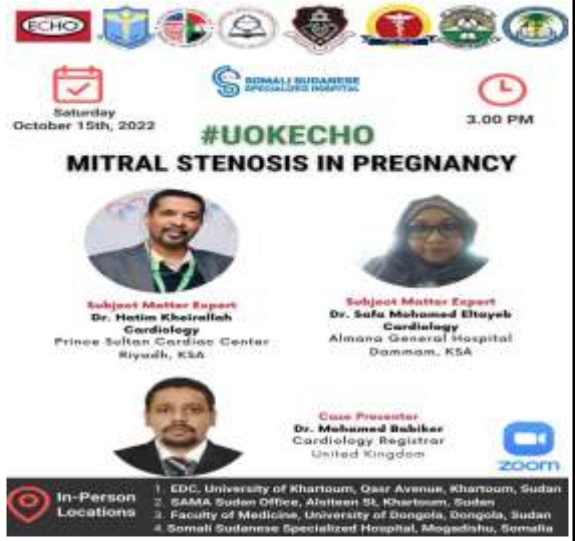

October 1st 2022, 3pm
Session on Adrenal Crisis

September 17th 2022 Lung Cancer
This session discussed an emergent health burden in Sudan, which is Lung Cancer. The didactics were presented by Dr. Omer Elhag, Consultant Pulmonologist and Dean of Medicine, University of Elneelain. Another didactic was presented by Dr. Mazin Almubasher, Consultant Pulmonologist and Associate professor at Omdurman Islamic University, which discussed the procedural thoracoscopic diagnosis in detail.

Learners attended from the Educational Development Center (EDC) in the Faculty of Medicine, University of Khartoum, Sudan, and the Somali Sudanese Specialized Hospital, Somalia, in addition to those logging in from their smartphones, laptops & other devices.

September 17th 2022 at 10 A.M Sudan time, to learn more about "Acute Abdomen".

September 10th 2022
topic:Diabetes

September 10th 2022
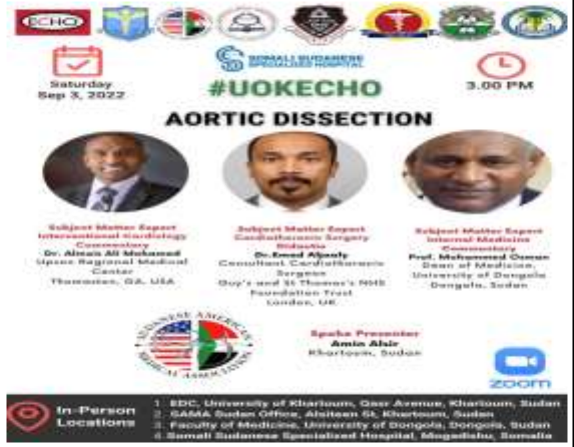

</td>
<td></td>
</tr>
</table>

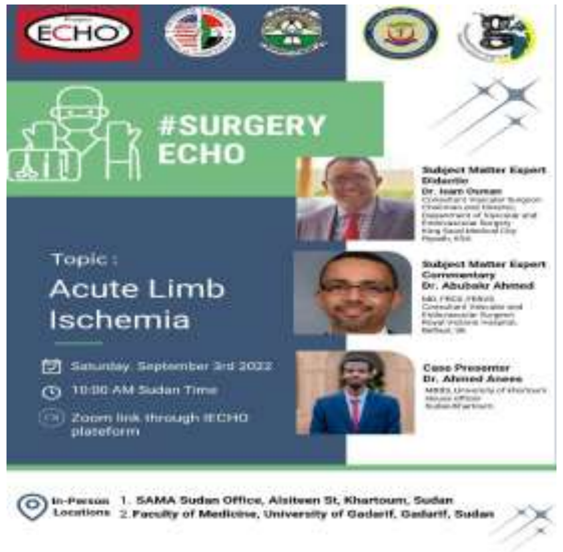

August 27th 2022- Dec 10 2022

We are thrilled to announce that the Sudanese Orthopedic Surgeons Association - SOSA will be partnering with SAMA in delivering #surgeryecho

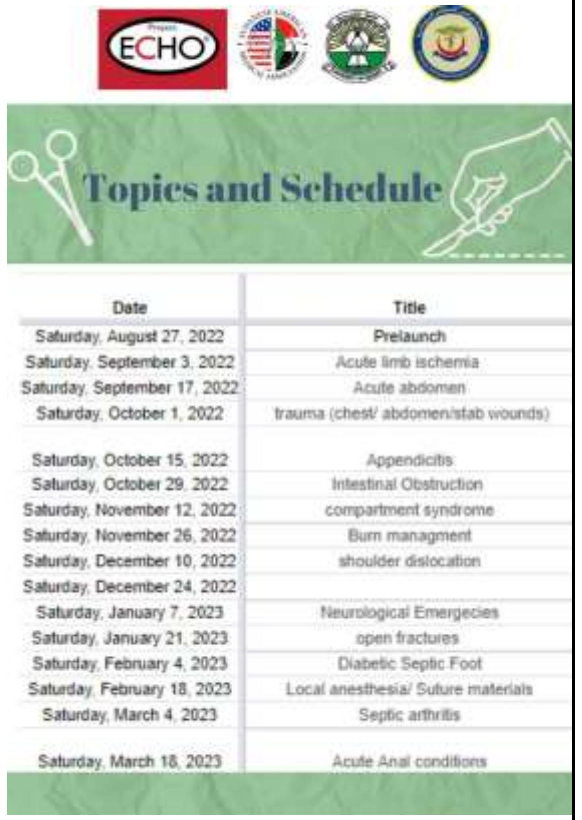

Date	Title
Saturday, August 27, 2022	Prelaunch
Saturday, September 3, 2022	Acute limb ischemia
Saturday, September 17, 2022	Acute abdomen
Saturday, October 1, 2022	trauma (chest/ abdomen/stab wounds)
Saturday, October 15, 2022	Appendicitis
Saturday, October 29, 2022	Intestinal Obstruction
Saturday, November 12, 2022	compartment syndrome
Saturday, November 26, 2022	Burn managment
Saturday, December 10, 2022	shoulder dislocation
Saturday, December 24, 2022	
Saturday, January 7, 2023	Neurological Emergecies
Saturday, January 21, 2023	open fractures
Saturday, February 4, 2023	Diabetic Septic Foot
Saturday, February 18, 2023	Local anesthesia/ Suture materials
Saturday, March 4, 2023	Septic arthritis
Saturday, March 18, 2023	Acute Anal conditions

20th August 2022

				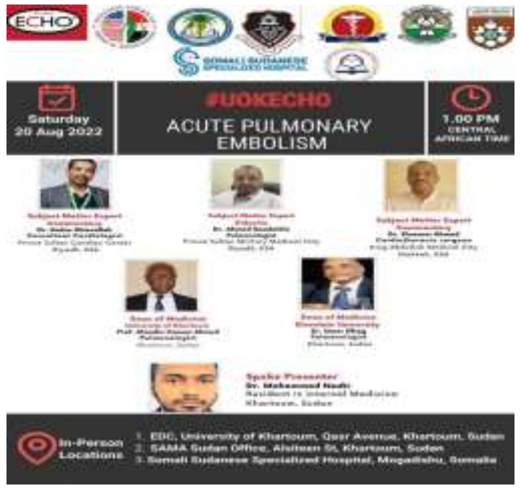<br><br>August 13th 2022<br>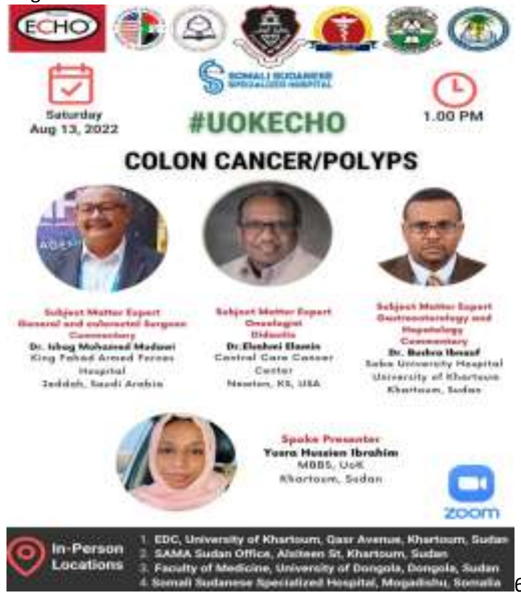<br><br>6th<br>August 2022<br>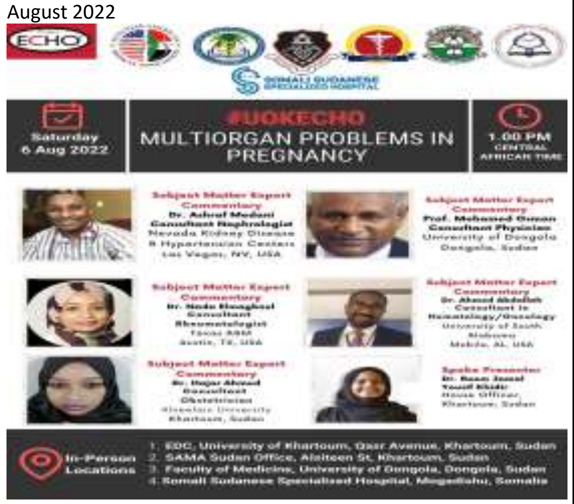<br><br>6th, 13th and 20th August 2022<br>Certificates from SAMA will be issued to those who attend the sessions and pass the post-test	

<table>
<tr>
<td></td>
<td></td>
<td></td>
<td></td>
<td>
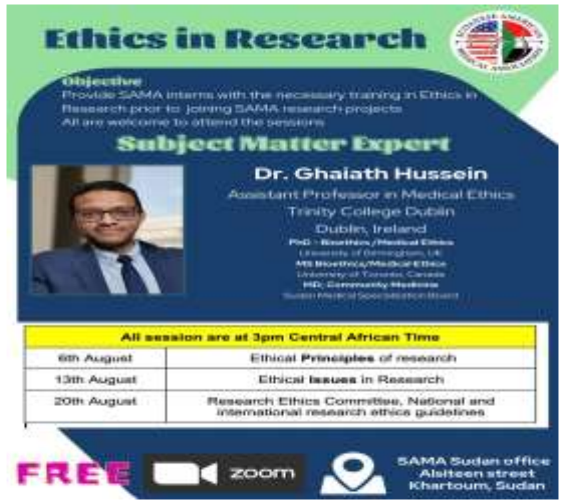

30th July 2022
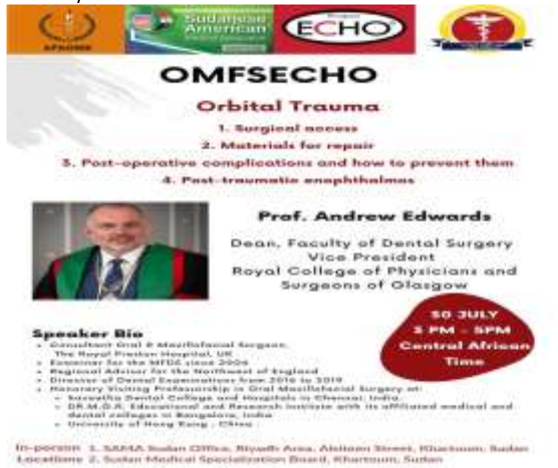

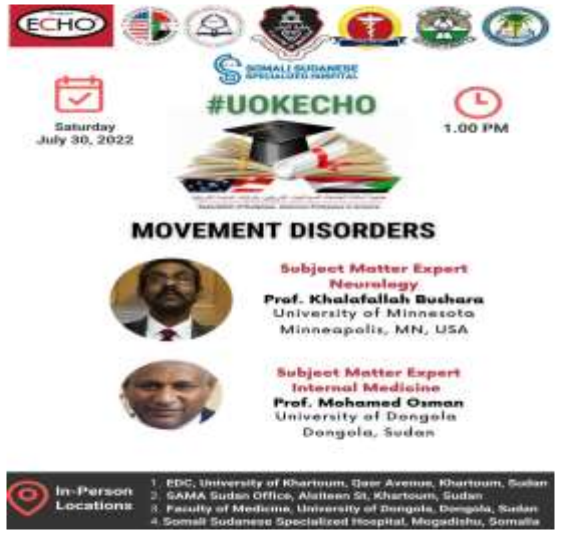

July 23rd 2022
</td>
<td></td>
</tr>
</table>

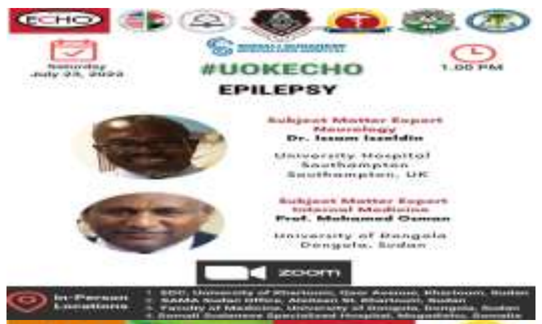

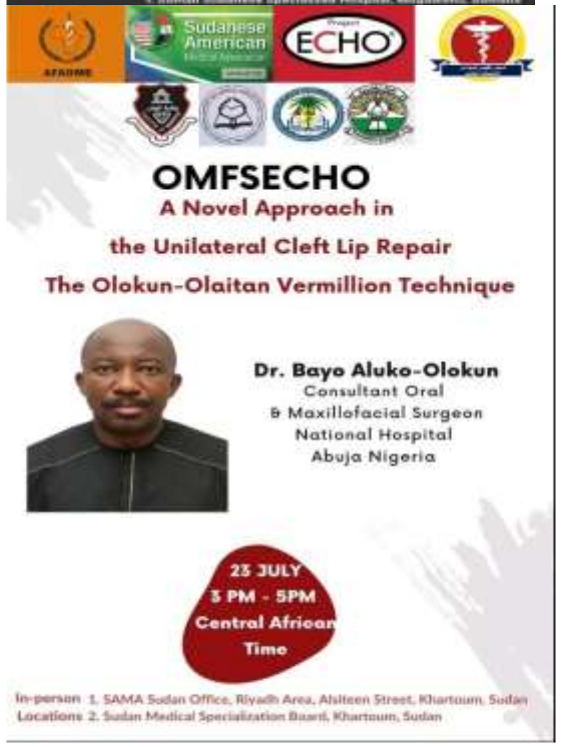

July 18th-22nd 2022
Virtual Reality in Medicine and Surgery

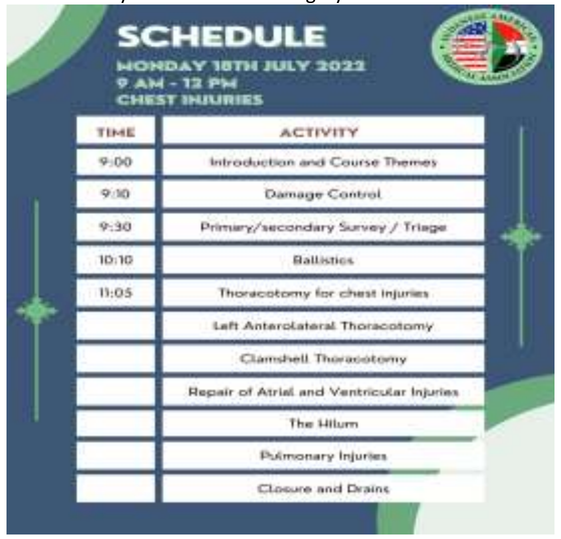

TUESDAY 19TH JULY 2022
9 AM - 2 PM
VISCERAL INJURIES

TIME	ACTIVITY
9:00	Appendicectomy
	Hernia Repair
	Trauma Laparotomy: Supracoeliac compression
	Pelvic Trauma: Internal iliac ligation
	Stomach Injuries
	Splenectomy
	Management of Liver Injuries
12:15	R to L Visceral Medial Rotation/IVC Repair
	Renal Repair and Nephrectomy
	Small Bowel Resection and Anastomosis
	Right Hemicolectomy and Anastomsis
	Left Hemicolectomy and Hartmanns procedure
	Rectal Injuries
	Stomas, Ileostomy and Colostomy

THURSDAY 21ST JULY 2022
9:30 AM - 4 PM
ONCOGYNAECOLOGY

TIME	ACTIVITY
9:30	Lectures:
	Entry techniques to abdomen
	Pelvic surgical anatomy
11:00	Cadaveric Dissection:
	HASSON open entry technique
	Retroperitoneal space access and dissection & pelvic spaces
	Step by step hysterectomy & pelvic node dissection
2:30	Lecture:
	Midline : open and closure tips & ttricks
3:00	Cadaver Dissection:
	Midline opening & Closure
	Omentectomy

FRIDAY 22ND JULY 2022
9:30 AM - 12 PM
TRANSPLANT SURGERY

TIME	ACTIVITY
9:30	Lectures:
	Donor nephrectomy
	Pancreas retrieval
	Pancreas and kidney transplantation
11:00	Cadaveric Dissection:
	Hand assist lap donor nephrectomy... including trouble shooting staple misfire
	open Pancreas transplantation
	Open kidney transplantation

16th July 2022

				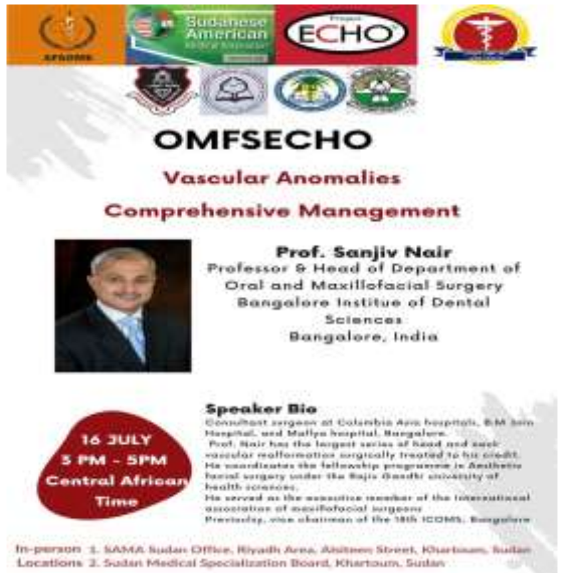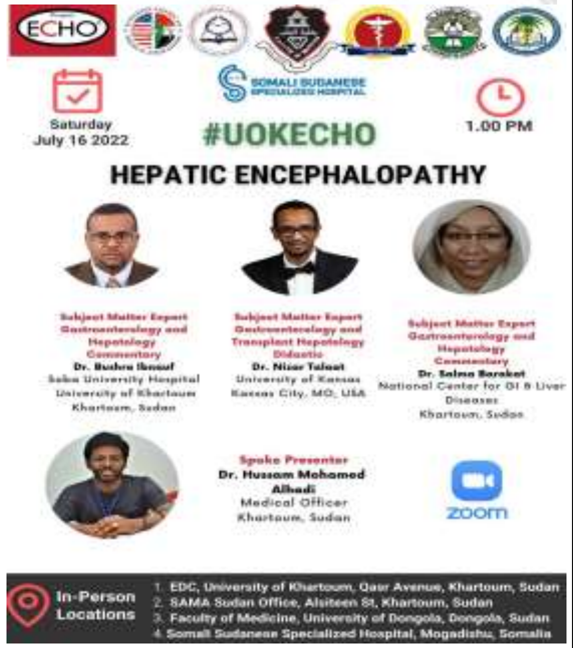2nd July 2022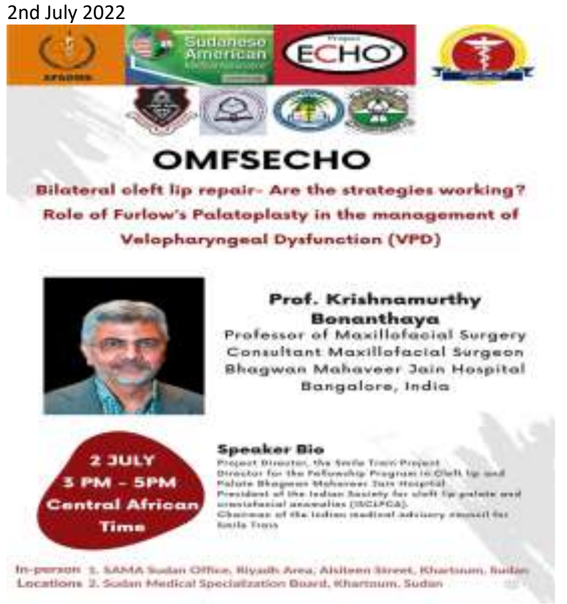	

				June 25th 2022<br>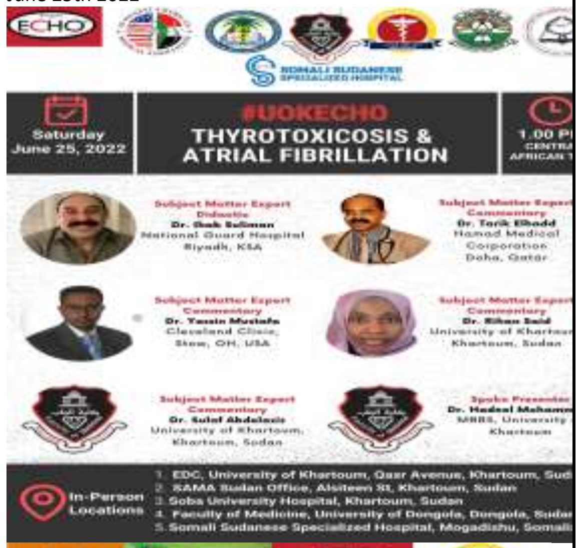<br><br>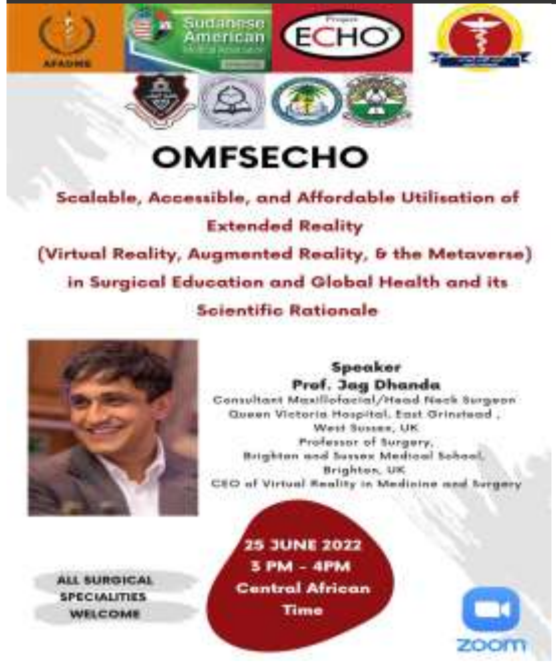<br><br>June 18th 2022<br>Topic: Contemporary Management of the Oral Cancer Patient & Mandible Reconstruction	

				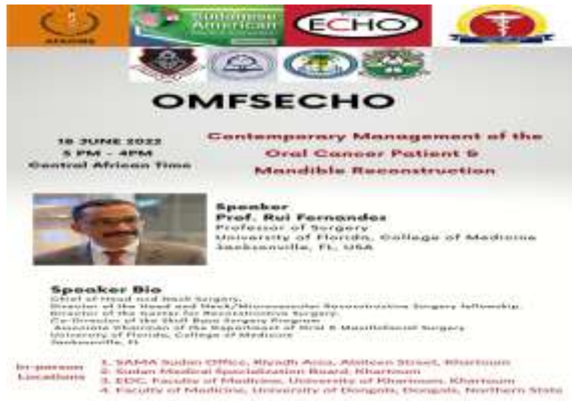<br><br>Topic:Stable Coronary artery disease<br>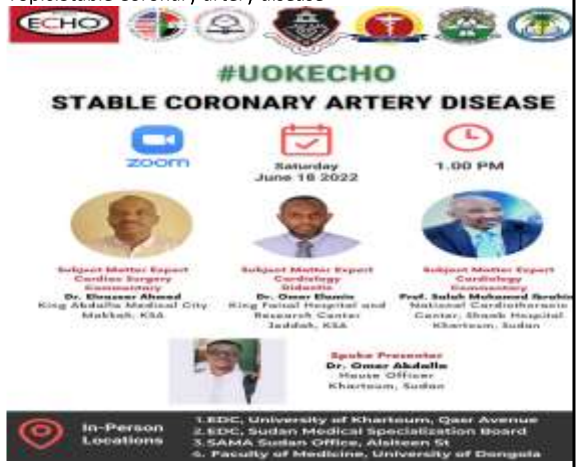<br><br>June 11th 2022	

<table>
<tr><td></td><td></td><td></td><td></td><td>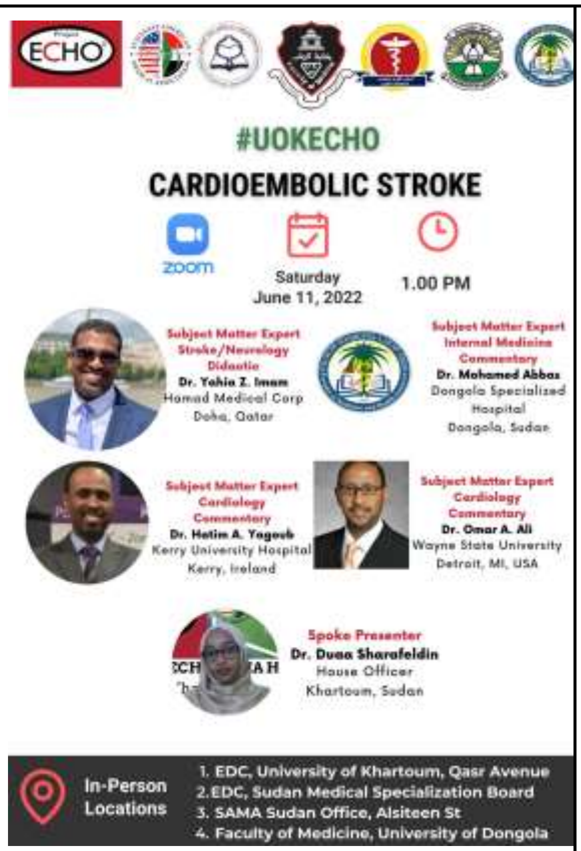

June 1st 2022
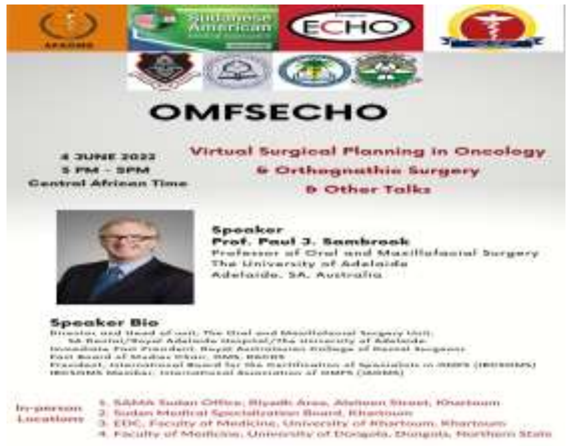

June 4th 2022
topic on cushing syndrome</td><td></td></tr>
</table>

<table>
<tr><td></td><td></td><td></td><td></td><td>
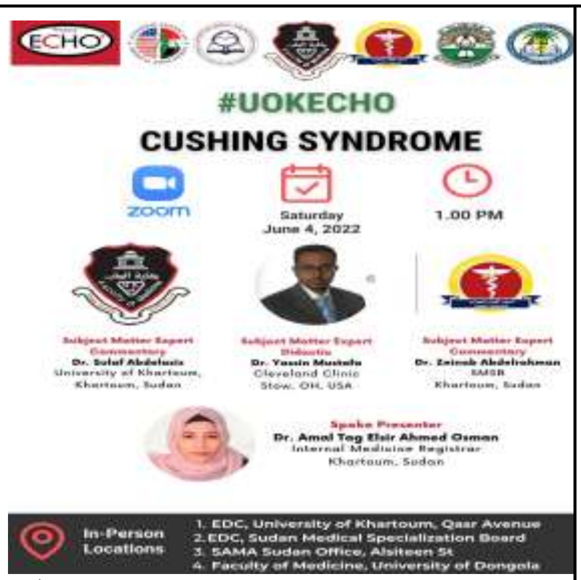

28th May 2022
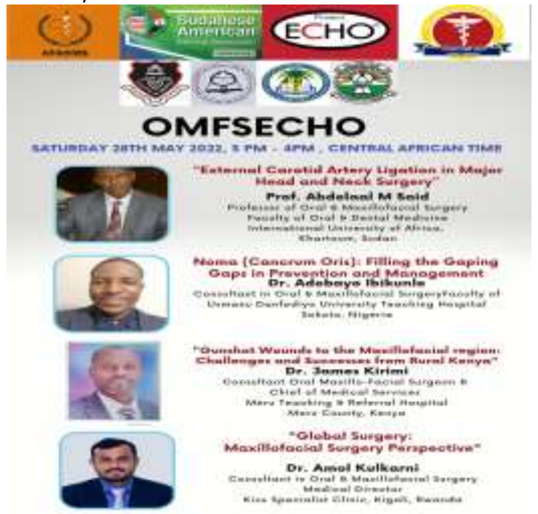

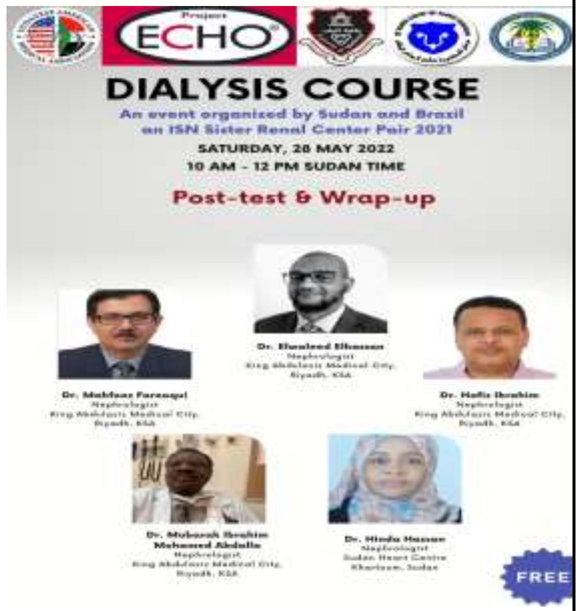

10th May 2022
Dialysis Course: Chronic Kidney diseae associated anemmia

10th May 2022
</td><td></td></tr>
</table>

<table>
<tr>
<td></td>
<td></td>
<td></td>
<td></td>
<td>Lecture on microvascular flaps
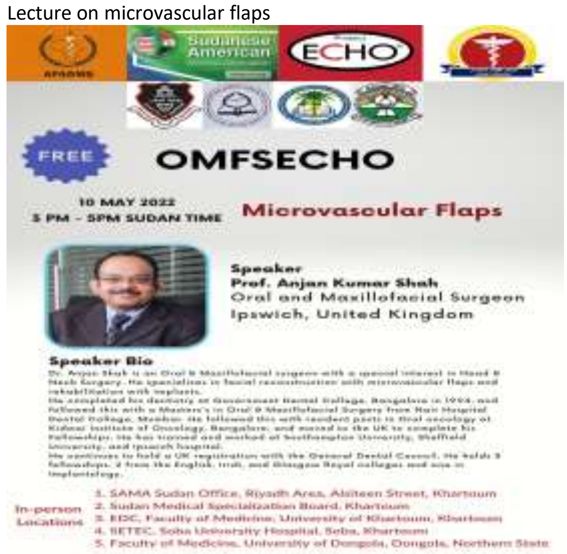

23rd April 2022
Dialysis course:Peritoneal dialisyis, for who and how?

16th April 2022
Dialysis course: Managing Hypertension in dialysis patient

12th March 2022
Dialysis course:Prevention and Treatment of Hemodialysis Catheter Infections
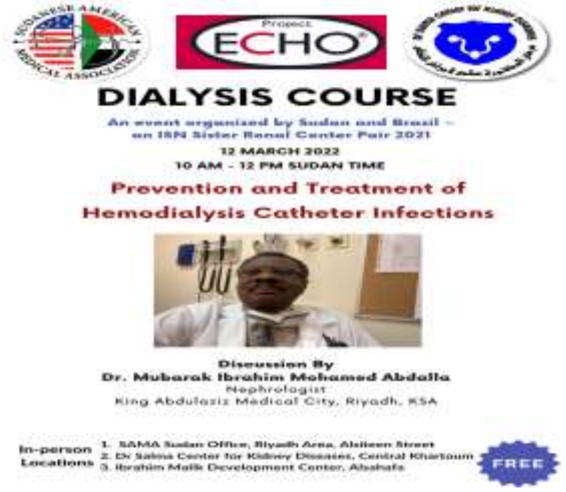

5th March 2022
Dialysis course</td>
<td></td>
</tr>
</table>

				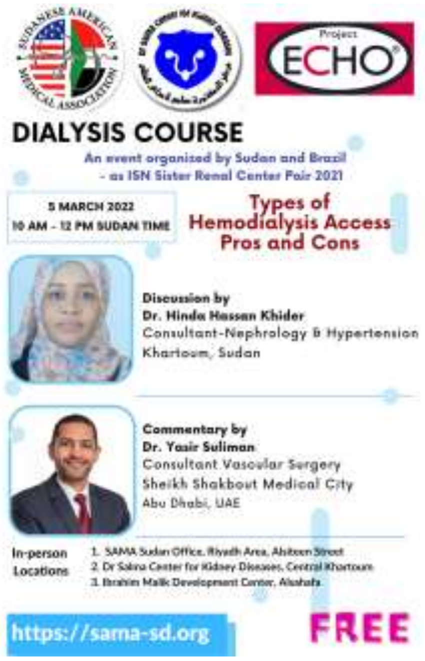<br>February 26th 2022<br>Dialysis course<br>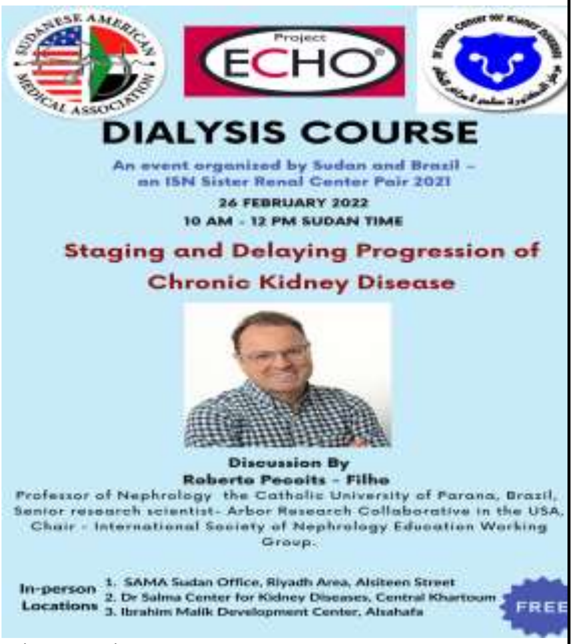<br>February 19th 2022<br>Dialysis course	

				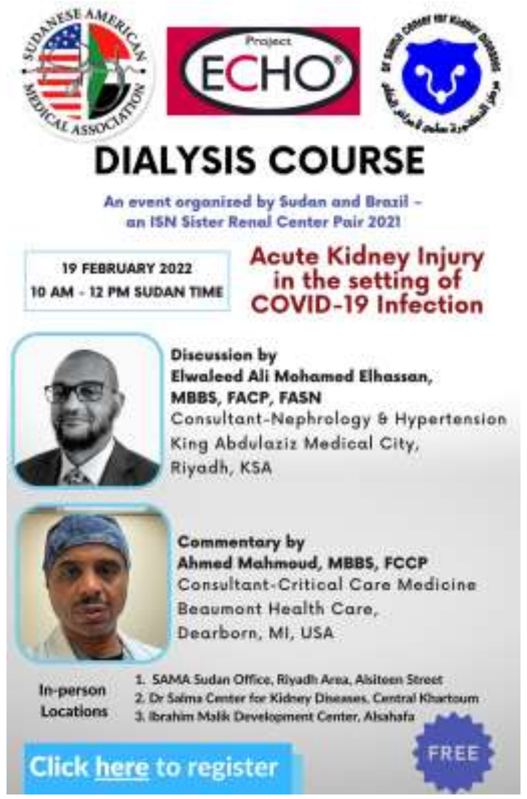<br><br>February 12th 2022<br>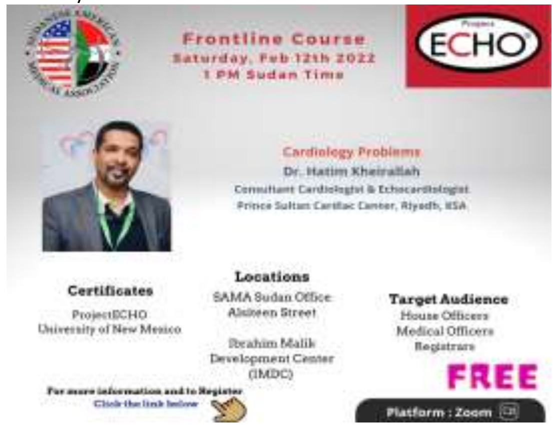<br><br>February 5th 2022<br>Lecture by Dr. Yagoub Marzoog Elshaikh Musa , Consultant Interventional Cardiologist, Heart Failure Lead, KSMC, Riyadh, KSA	

				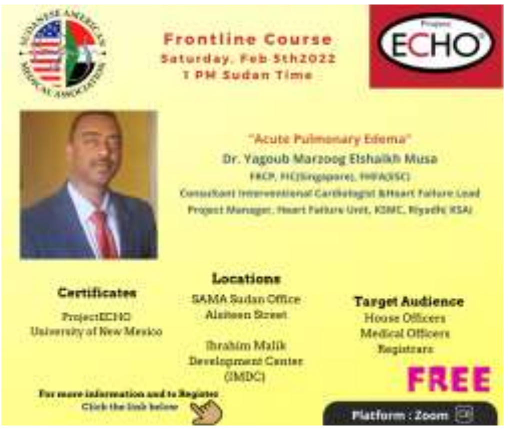<br><br>January 29th 2022<br>Topic was Mechanical Complications of Acute Myocardial Infarction presented by Dr. Elsadig Askar, Senior Interventional Cardiologist, Shaab Teaching Hospital, Khartoum, Sudan. Audience attended from SAMA Sudan Office, and Ibrahim Malik Teaching Hospital (in addition to personal devices)<br>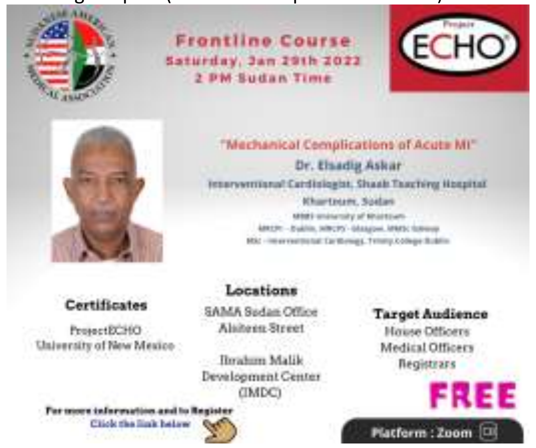<br><br>January 22nd 2022<br>Cardiac tamponade session<br>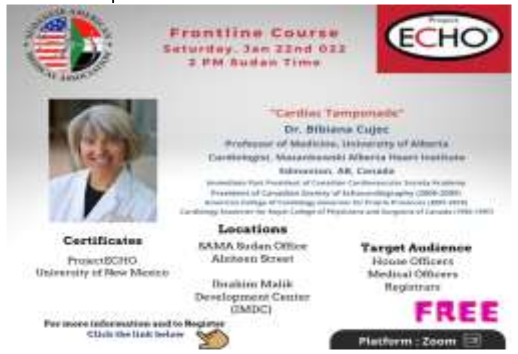<br><br>2nd October-23rd October 2021<br>Courses on Arrythmias	

				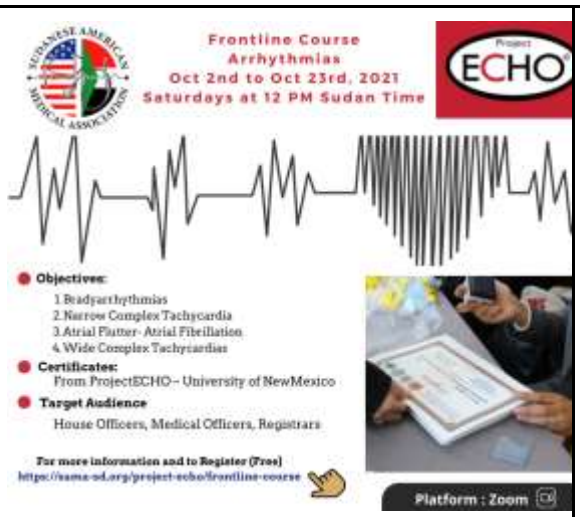<br>September 23 2021<br>lecture on Chronic Kidney Diseases<br>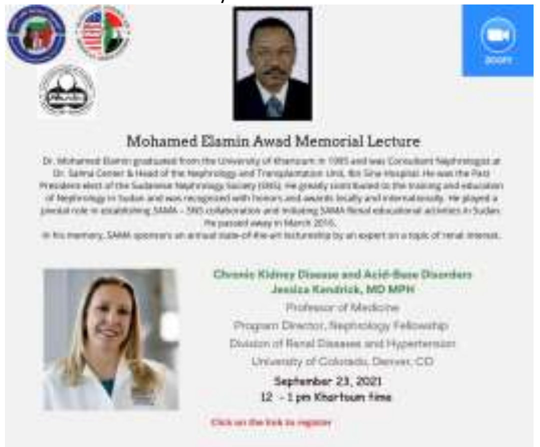<br>Aug,21st to Dec 25th, 2021<br>Saturdays at 12PM Sudan Time<br>Frontline Course, Online by Zoom<br>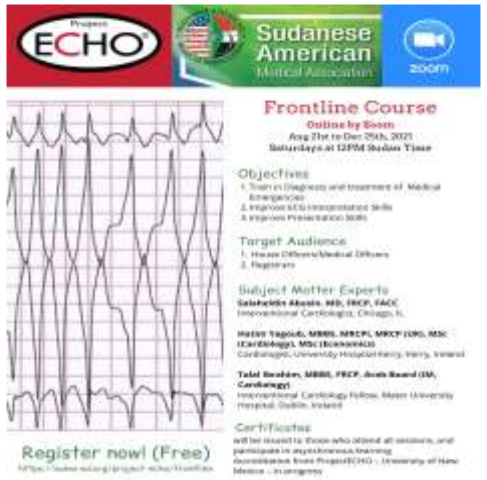	
Sudanese Junior Doctors Association UK	https://www.sjda.uk	SJDA-UK is an academic, social and non- political organisation founded by Sudanese junior doctors in the UK to connect and benefit the Sudanese doctors in training in the UK and Sudan.<br>Aim:	NA	August 7th 2023<br>SJDA Live Webinar **<br>have you joined a training post recently or new to the NHS in general and struggling to understand the E-Portfolio ?<br>If so, Dr Babiker is doing a quick LIVE session tomorrow at 8 pm (UK Summer Time), to go through the basics of the E-Portfolio	18TH January 2023<br>an online one-day CST interview course on Sunday 26/02/23 to help our colleagues who will be shortlisted for the interviews to get higher points, and increase their chances to get training numbers.<br>The course will involve talks about the different stations in addition to mock interviews with detailed feedback.

		Career Guidance<br>To help the Sudanese junior doctors in the UK to get training jobs/ training numbers in their field of interest; by providing advice, career planning and peer support<br>UK Guidance<br>To help Sudanese doctors outside the UK who wish to come here by providing simplified information and Sudanese specific road plan.<br>Connect & Network<br>To connect and network the Sudanese Junior Doctors in the UK ; To use each other as a resource if need be either socially or professionally.		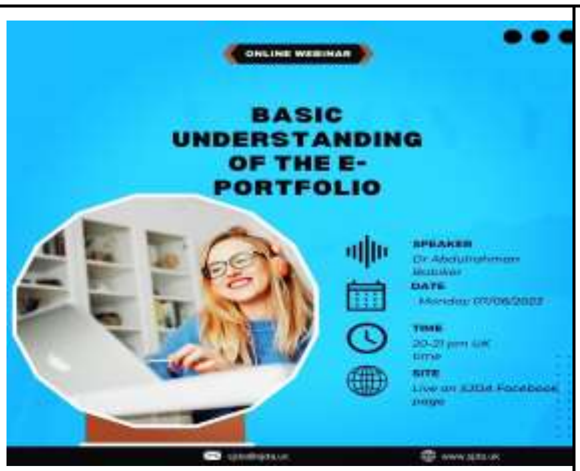<br>Date: 27th May 2023<br>Venue: BCEC, Hill St, Birmingham B5 4EW<br>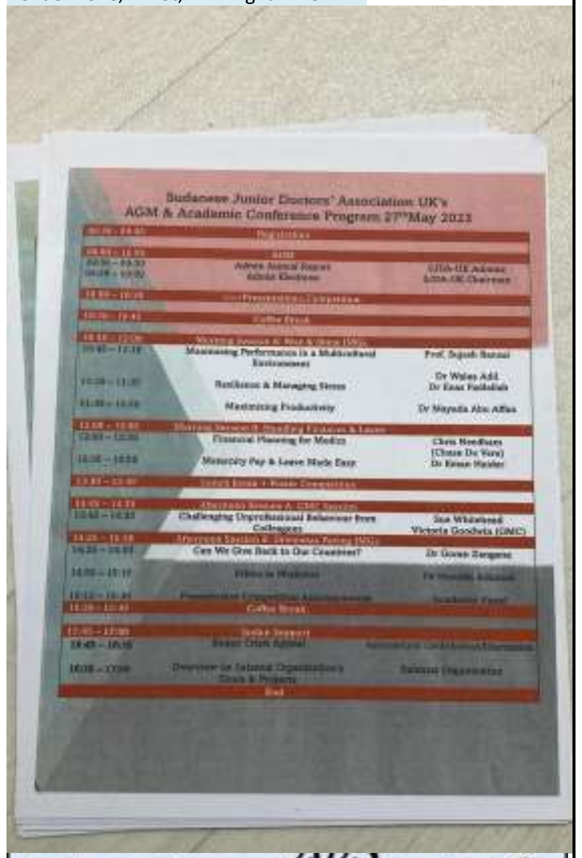<br>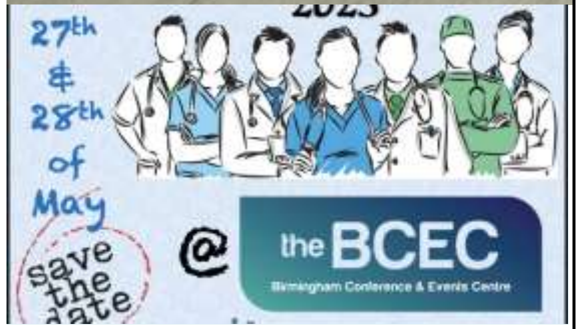<br>1st November-17tht November 2022<br>Surgical and emergencies Teaching webinars	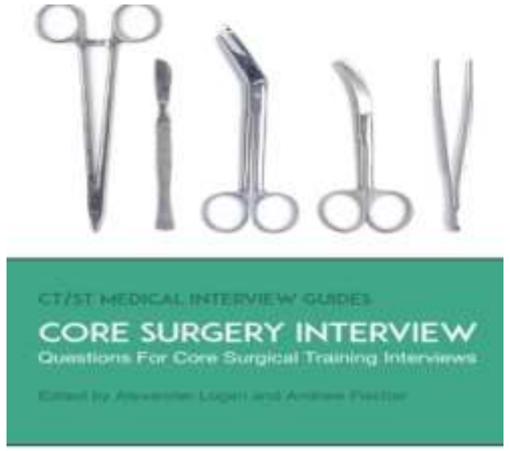<br>16TH December 2022<br>Join us tomorrow!This live video will be useful for those who are planning to take the MSRA exam soon and equally those who are interested in a career in Psychiatry!<br>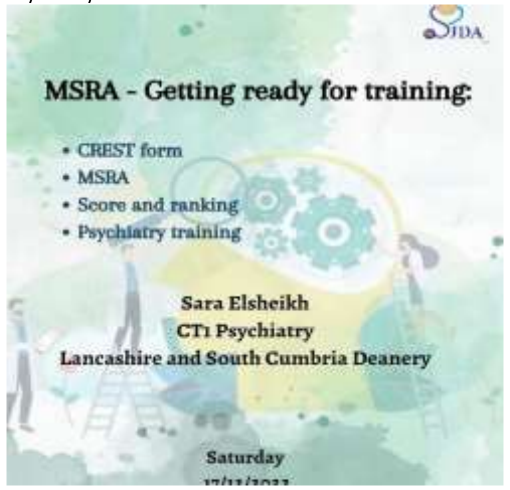<br>3rd December 2022<br>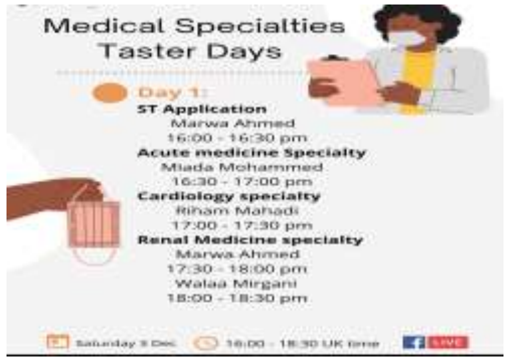

				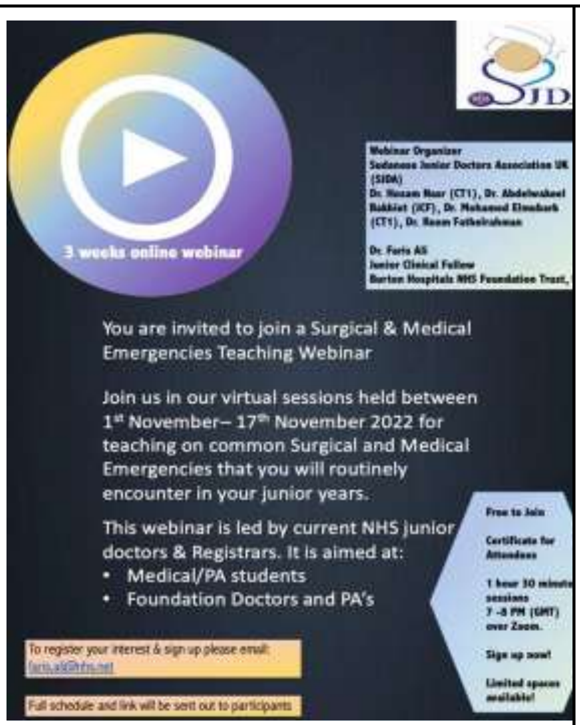<br>Nov 1st, 7th and 14th 2022<br>SJDA Webinar schedules<br>Educational webinars on medical tropics<br>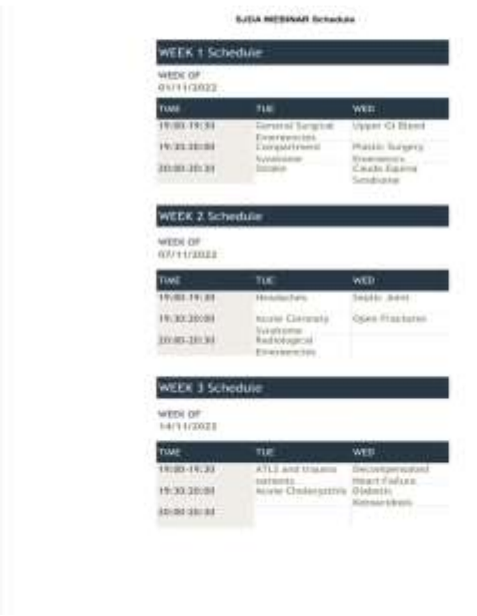<br>Date: 14th May 2022<br>Venue: INNSIDE Manchester, 1 First St, Manchester M15 4RP	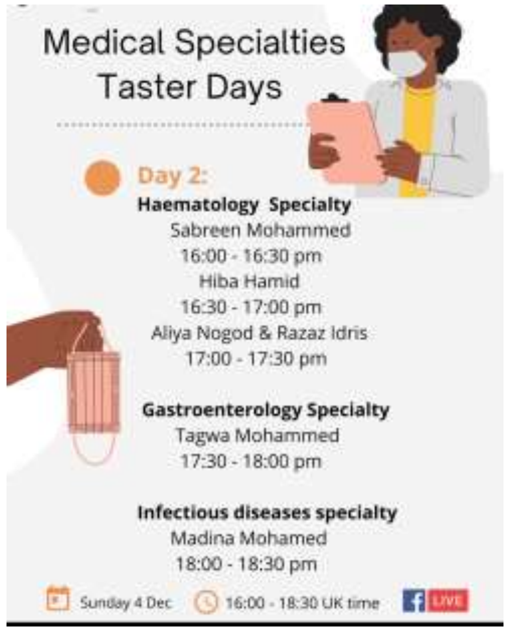<br>25th November 2022<br>This live video will be useful for those who are applying for histopathology training this year or considering it<br>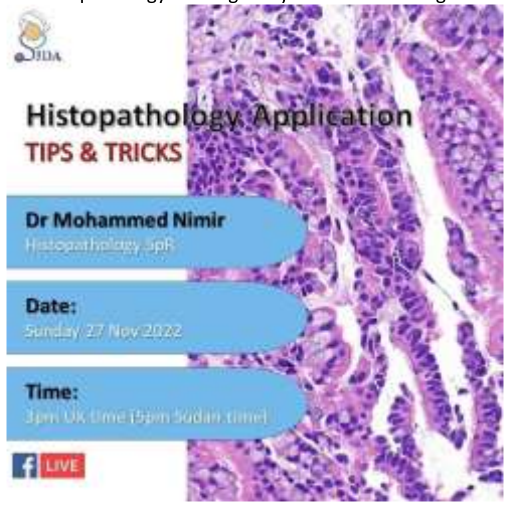<br>3rd November 2022<br>Live on Facebook this weekend!

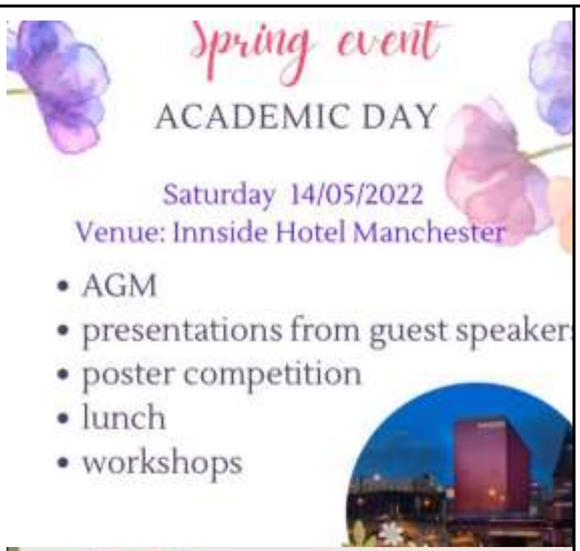

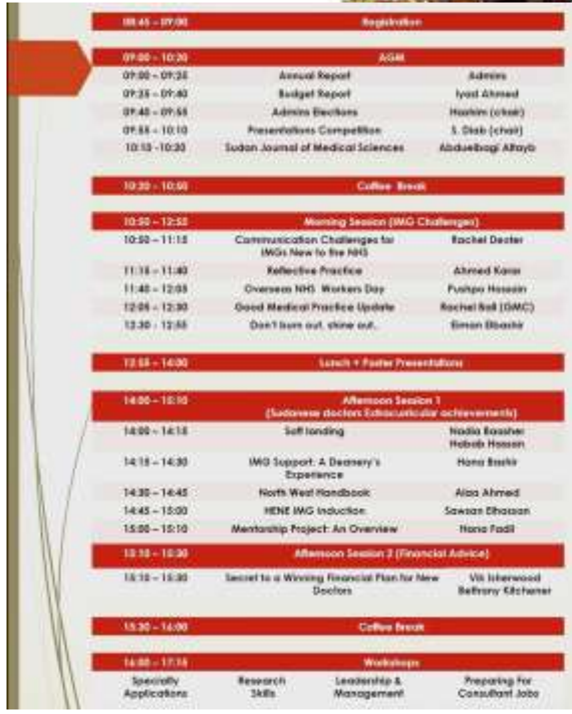

08:45 – 09:00	Registration	
09:00 – 10:20	AGM	
09:00 – 09:25	Annual Report	Admins
09:25 – 09:40	Budget Report	Iyad Ahmed
09:40 – 09:55	Admins Elections	Hashim (chair)
09:55 – 10:10	Presentations Competition	S. Diab (chair)
10:10 - 10:30	Sudan Journal of Medical Sciences	Abduelbagi Altayb
10:30 – 10:50	Coffee Break	
10:50 – 12:55	Morning Session (IMG Challenges)	
10:50 – 11:15	Communication Challenges for IMGs New to the NHS	Rachel Dexter
11:15 – 11:40	Reflective Practice	Ahmed Karar
11:40 – 12:05	Overseas NHS Workers Day	Pushpa Hossain
12:05 – 12:30	Good Medical Practice Update	Rachel Ball (GMC)
12:30 - 12:55	Don't burn out, shine out.	Eiman Elbashir
12:55 – 14:00	Lunch + Poster Presentations	
14:00 – 15:10	Afternoon Session 1 (Sudanese doctors Extracurricular achievements)	
14:00 – 14:15	Soft landing	Nadia Basher Habab Hassan
14:15 – 14:30	IMG Support: A Deanery's Experience	Hana Bashir
14:30 – 14:45	North West Handbook	Alaa Ahmed
14:45 – 15:00	HENE IMG Induction	Sawsan Elhassan
15:00 – 15:10	Mentorship Project: An Overview	Hana Fadil
15:10 – 15:30	Afternoon Session 2 (Financial Advice)	
15:10 – 15:30	Secret to a Winning Financial Plan for New Doctors	Vik Isherwood Bethany Kitchener
15:30 – 16:00	Coffee Break	
16:00 – 17:15	Workshops	

Specialty Applications	Research Skills	Leadership & Management	Preparing For Consultant Jobs

15th March 2021
Internal medicine Educational Programm:Complication of liver disease and transplantation

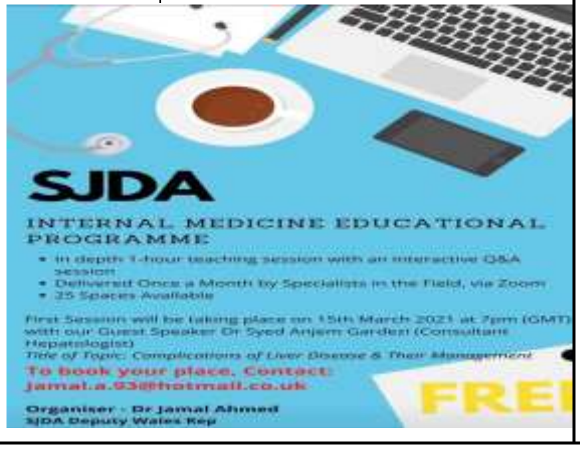

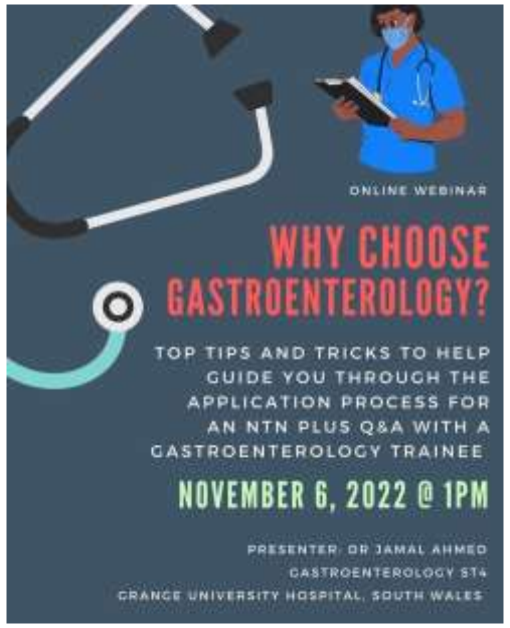

26th October 2022
SJDA is back again with an online interactive zoom session aiming to answer all of your questions re:
Applying to work in the UK
All the paperwork involved
Particular questions on certain specialities etc
Date: Wednesday 26th of October
Time: 7-8pm UK time

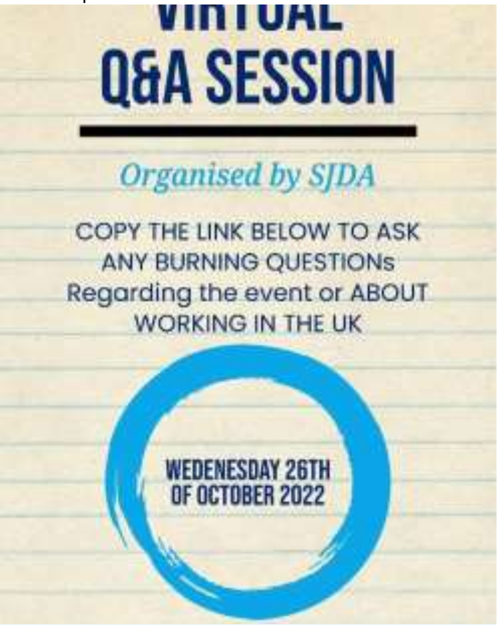

16th October 2022
Live video will be about Plastics surgery training in the UK

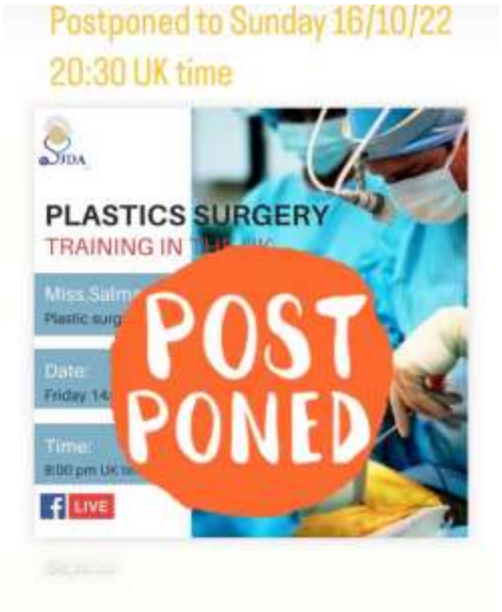

1st October 2022
Orthopeadics Training in the UK

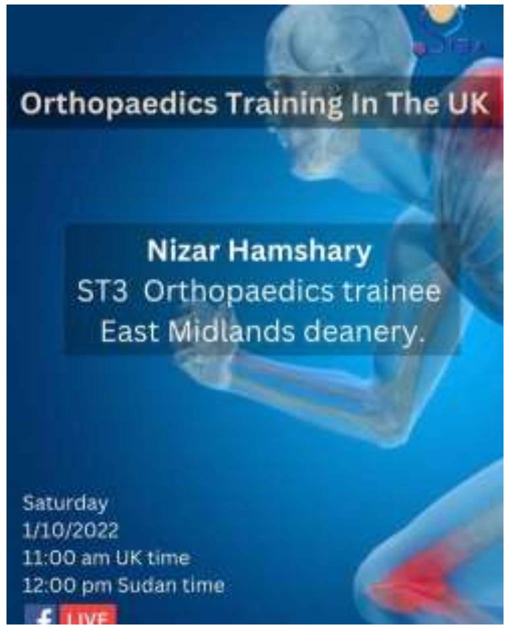

11th September 2022
Join us tomorrow at 6pm UK time 7pm Sudan time
ST3 Surgical training in the UK
Live on Facebook

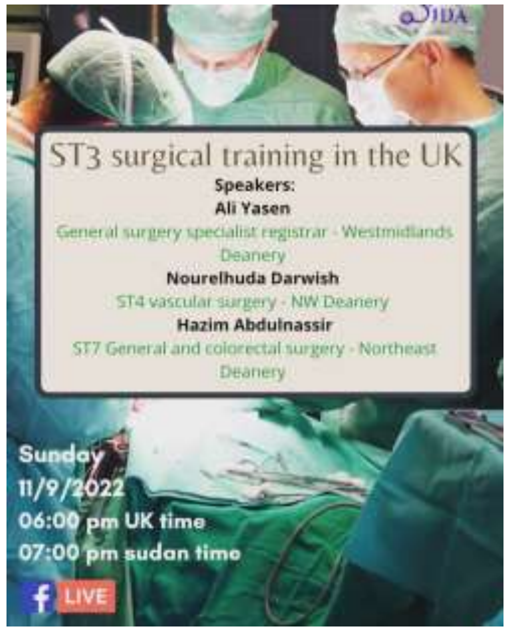

31st July 2022
How to enter urology training in the UK.
Mr Wail Gaffar one of the Sudanese trainees will tell you all about it.

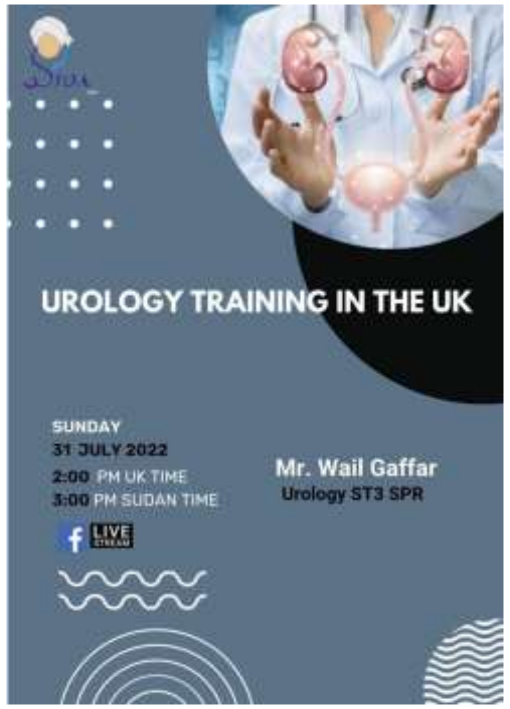

17th July 2022
Facebook Live Stream
Those who are in the process of applying for their GMC registration and those who are in the beginning of their journey don't forget to join us.

					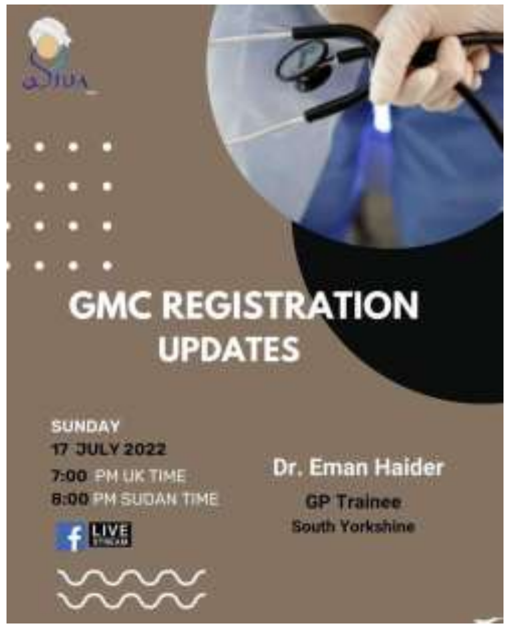<br>20th June 2022<br>Dr Samar Hassan will be presenting live on Facebook: Dermatology training in the UK<br>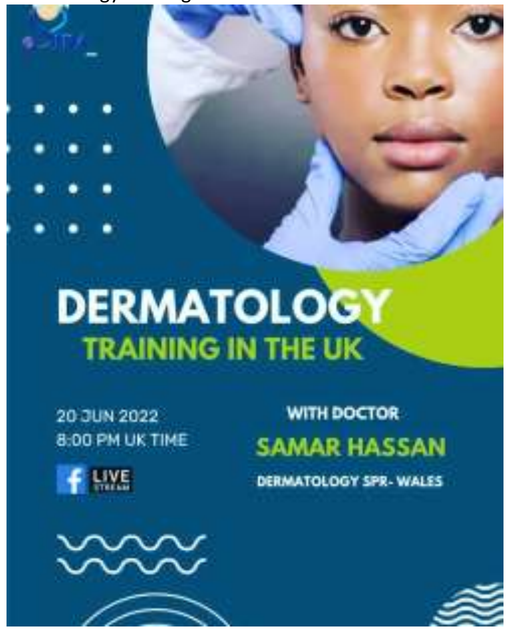<br>January 19th 2022<br>Calling all IMGs interesting in Acute Medicine training in the UK...<br>takeAIM/ Society for Acute Medicine (SAM) in collaborations with Sudanese Junior Doctors Associations in the UK (SJDA UK) is holding a webinar to help address what IMGs need to know to start training in AIM.<br>Please join us on Sunday evening 23rd January 2022 at 18:00 London time, 20:00 Khartoum time.

<table>
<tr><td></td><td></td><td></td><td></td><td></td><td>
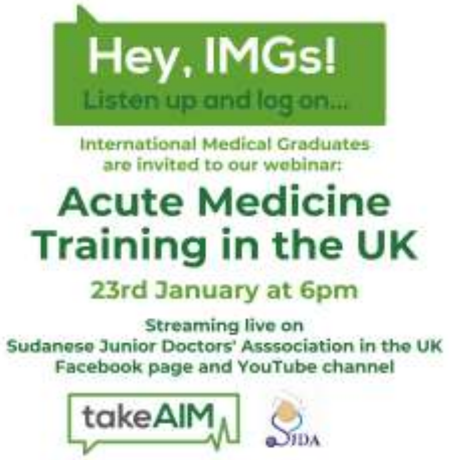

26th-28th November 2021
For those who are applying for training or have an interest to pursue a career in certain medical specialties SJDA is delighted to invite you to our Medical specialties taster days
Please Join our Zoom meeting this weekend !

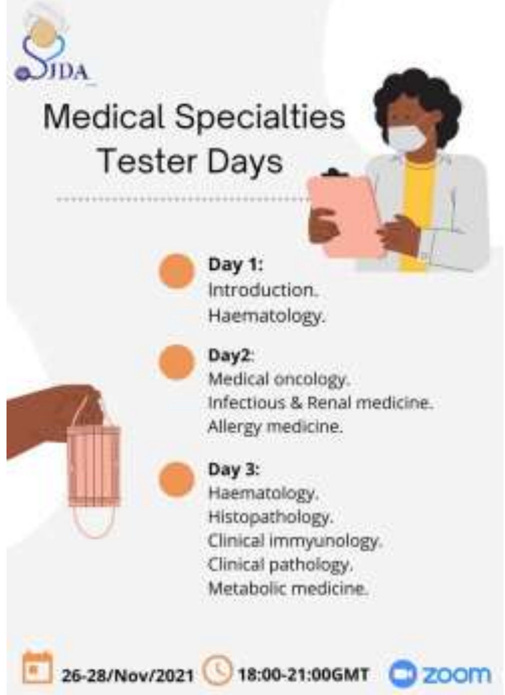

2nd January 2021
Our annual interview workshop will be virtual this year via Zoom
</td></tr>
</table>

					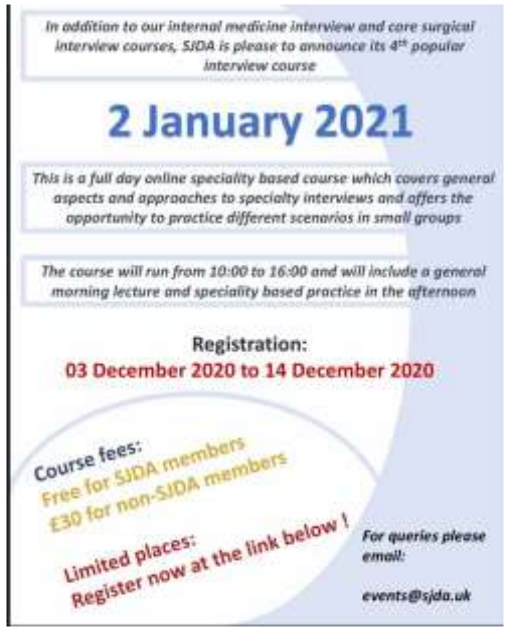<br>vvvv
Sudanese Medical Association UK and Ireland	https://www.facebook.com/groups/sma.ukandire/?ref=group_header	The Association is aiming to be an agent of change and voice of reason by representing the public face of Sudanese medicine and bringing healthcare professionals together in working partnership.	NA	NA	NA
Syrian American Medical Society Foundation	https://www.sams-usa.net	SAMS is dedicated to delivering life-saving services, revitalizing health systems during crisis, and promoting medical education via a network of humanitarians in Syria, the US, and beyond.	NA	June 30th – July 2nd 2023<br><br>We are honored to have your attendance at SAMS 21st International Conference, “Research in Hybrid Crisis: Confronting Health Needs in Conflict and Natural Disasters” in Vancouver, Canada<br>This year, our Scientific Program focuses on utilizing research and medical education to catalyze change in a crisis. The program includes sessions on overcoming treatment barriers and advancing research in disaster nephrology, the effects of the crisis on mental health, data science innovations in hybrid crisis research, medical education during conflict and natural disasters, updates on SAMS specialty programs, the ethical problems of triage in wartime, and much more. The conference will feature lectures by esteemed professionals and includes upwards of 6 hours of CME credit!<br><br>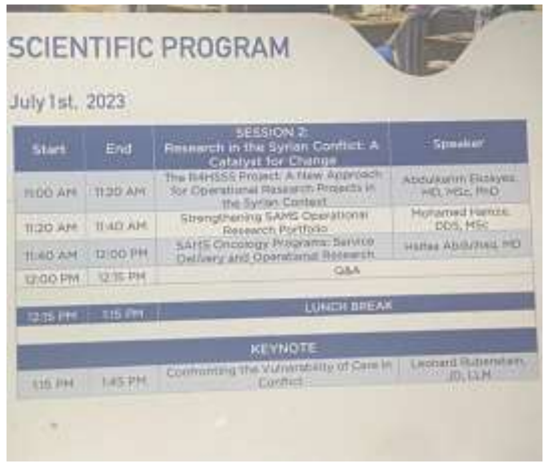	NA<br>(not within timeframe for inclusion criteria)

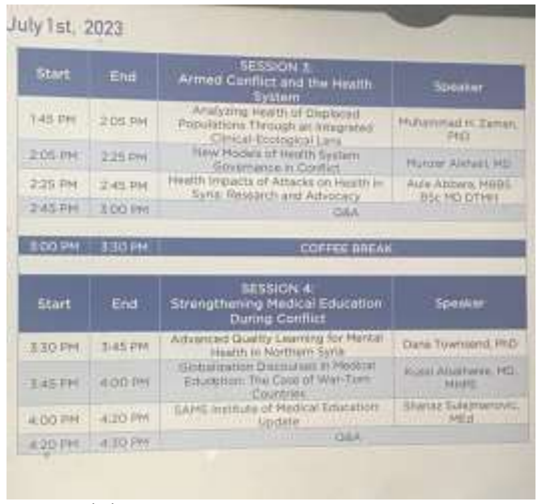

July 1st, 2023

Start	End	SESSION 3: Armed Conflict and the Health System	Speaker
1:45 PM	2:05 PM	Analyzing Health of Displaced Populations Through an Integrated Clinical-Ecological Lens	Muhammad H. Zaman, PhD
2:05 PM	2:25 PM	New Models of Health System Governance in Conflict	[illegible], MD
2:25 PM	2:45 PM	Health Impacts of Attacks on Health in Syria: Research and Advocacy	Aula Abbara, MBBS BSc MD DTMH
2:45 PM	3:00 PM	Q&A	
3:00 PM	3:30 PM	COFFEE BREAK	

Start	End	SESSION 4: Strengthening Medical Education During Conflict	Speaker
3:30 PM	3:45 PM	Advanced Quality Learning for Mental Health in Northern Syria	Dana Townsend, PhD
3:45 PM	4:00 PM	Globalization Discourses in Medical Education: The Case of War-Torn Countries	[illegible], MD, MHPE
4:00 PM	4:20 PM	SAMS Institute of Medical Education Update	[illegible], MEd
4:20 PM	4:30 PM	Q&A	

Sunday 7/2/2023 5:00 PM: Sailaway, Alaska Glacier Experience
Royal Caribbean, Brilliance of the Seas

February 17, 2023 - February 18, 2023

SAMS 6th National Symposium, Tampa. Innovations &Transformations in Healthcare

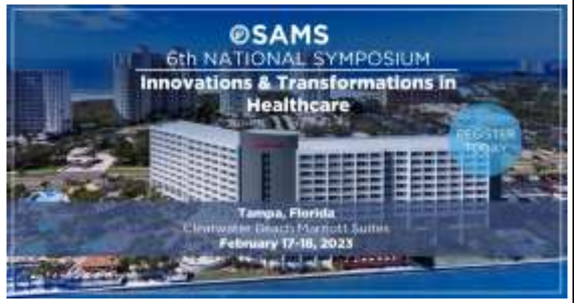

September 3rd to 5th, 2022.
SAMS is hosting its 11th National Conference in Chicago, Illinois
The scientific committee is looking for presentations and speakers who can provide their unique perspectives and experiences that fall under the theme of the event, "Promoting Healthy Communities Through Medical Science, Public Health Policy, and Interventions" or any of the following other areas of expertise:

- Public health policies and preventions
- U.S. healthcare reform;
- Prevention and screening guidelines
- Emerging and re-emerging infectious diseases
- Cancer screening guidelines
- Medical updates;
- Innovations in medicine;
- COVID-19 pandemic-related challenges;
- Management of acute illness and trauma in times of crisis;
- Management of chronic illnesses in times of crisis;
- Psychiatric health at a time of crisis;
- Physical medicine and rehabilitation;

				• Community health outreach and provision of free medical services;<br>• Professional development and well-being of healthcare professionals.<br>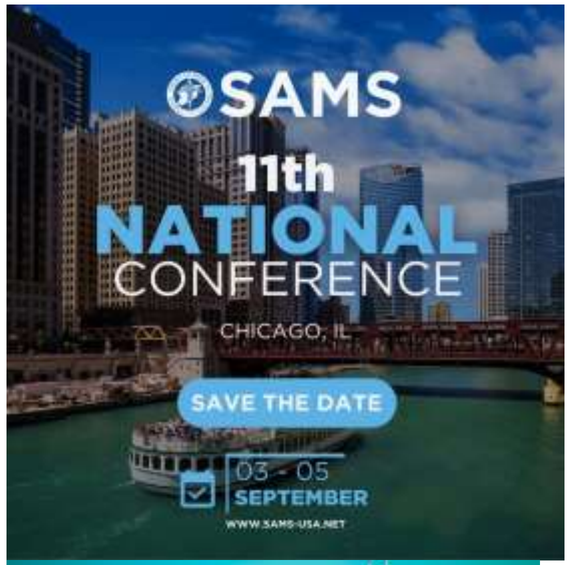<br><br>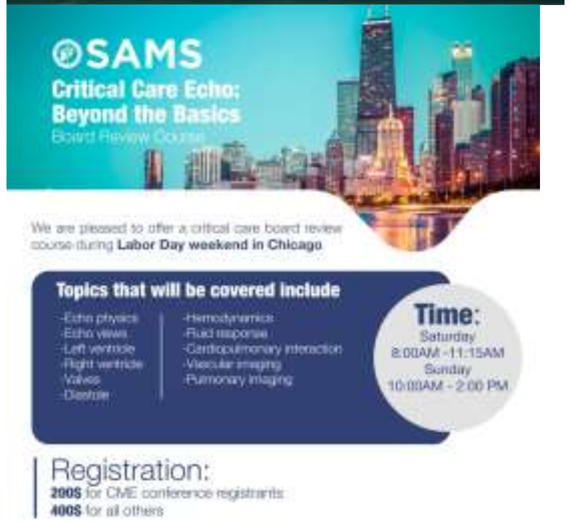<br><br>SAMS is pleased to offer a critical care board review course during Labor Day weekend in Chicago, "SAMS Critical Care Echo: Beyond the Basics" during SAMS 11th National Conference in Chicago, Illinois from September 3rd to 5th, 2022.<br><br>Topics that will be covered include:<br>Echo physics<br>Echo views<br>Left ventricle<br>Right ventricle<br>Valves<br>Distal<br>Hemodynamics<br>Fluid Response<br>Cardiopulmonary interaction<br>Vascular Imaging<br>Pulmonary Imaging<br>The course will take place on September 3, 2022, 8:00 AM - September 4, 2022, 2:00 PM (EDT).	

				June 24 – 26, 2022.<br>SAMS is hosting its 20th International Conference in Istanbul, Turkey<br>The scientific committee is looking for presentations and speakers who can provide their unique perspectives and experiences that fall under the theme of the event, "Closing the Equity Gap in the Pandemic Era and Beyond through Innovation," or any of the following other areas of expertise:<br><br>• Health systems strengthening and early recovery;<br>• Medical updates;<br>• Innovations in medicine; telemedicine; artificial intelligence;<br>• COVID-19 pandemic related challenges;<br>• Public health issues;<br>• U.S. healthcare reform;<br>• Management of acute illness and trauma in times of crisis;<br>• Management of chronic illnesses in times of crisis;<br>• Psychiatric health at a time of crisis;<br>• Physical medicine and rehabilitation;<br>• Community health outreach and provision of free medical services;<br>• Healthcare disparities and inequities;<br>• Professional development and well-being of healthcare professionals.<br><br>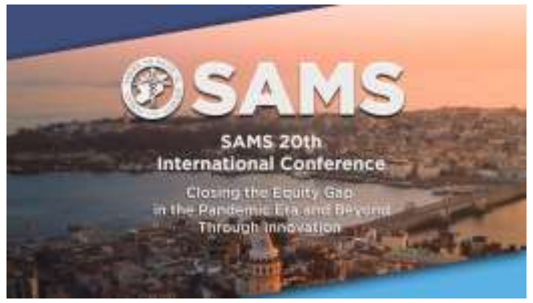<br><br><br>September 3-5, 2021.<br>SAMS is hosting its 10th Annual National Conference in Chicago, Illinois	

				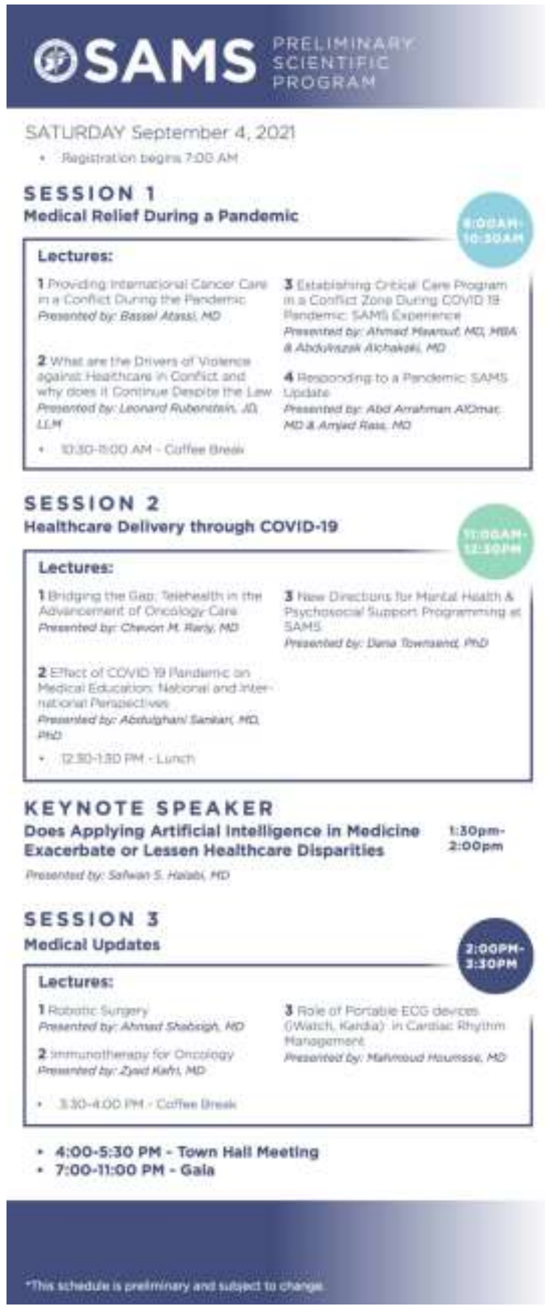<br><br>July 23, 2021.<br>SAMS is hosting its 2nd International Symposium, "COVID-19 Pandemic: Lessons Learned Moving Forward" in Trabzon, Turkey<br>Attendees will learn about the latest updates to treatment and vaccination for COVID-19, its impact on the ground, and SAMS' response to this global health crisis. The symposium will include upwards of 8 hours of CME lectures	

				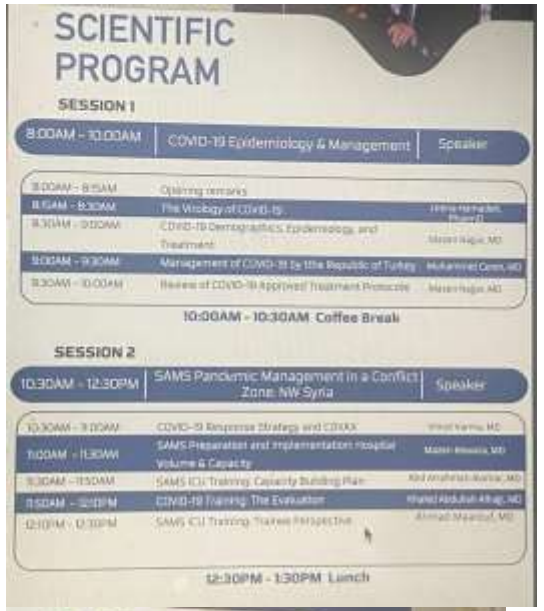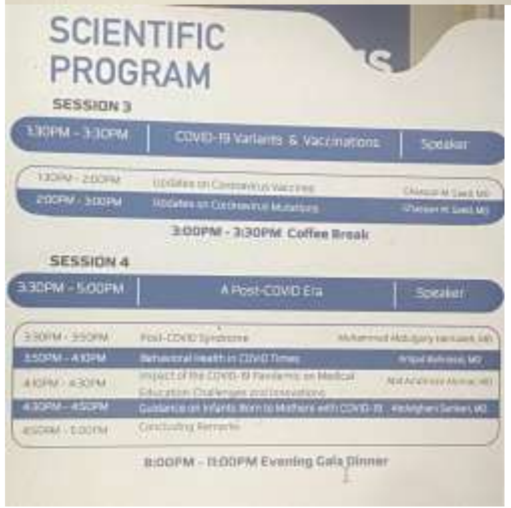	
Syrian British Medical Society	http://sb-ms.org	Its main objectives are to support British-Syrian healthcare professionals with their training and progression as well as Syrian healthcare professionals in Syria or in the diaspora. In particular, to:<br><br>Support Syrian healthcare professionals with training, academic progression, educational opportunities and networking in the UK and abroad.<br><br>Provide worldwide representation of Syrian healthcare professionals working in the UK.<br><br>Promote local and international collaborations/ networks with Syrian and non-Syrian academic and professional organisations which promote and further the	NA	October-December 2021 - General Surgery telemedicine training course with SBOMS (Syrian Board of Medical Specialties) and the David Nott Foundation. SBMS members contributed to the delivery of surgical training to Syrian surgeons with SBOMS.	Saturday 26th November 2022 1800-199 UK<br>We plan a panel of SBMS committee and other members who have recent experience of GMC registration and examinations (IELTs/OETS and PLAB 1 & 2) as well as membership (MRCP, MRCS and others.)<br>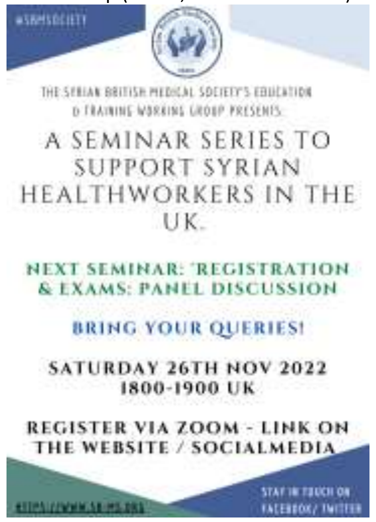

		understanding of Syrian healthcare and Syrian culture.<br><br>Advocate for issues pertinent to the health of Syrians globally including Syrian refugees.<br><br>Contribute to the development of the health system in Syria and other health systems in need.		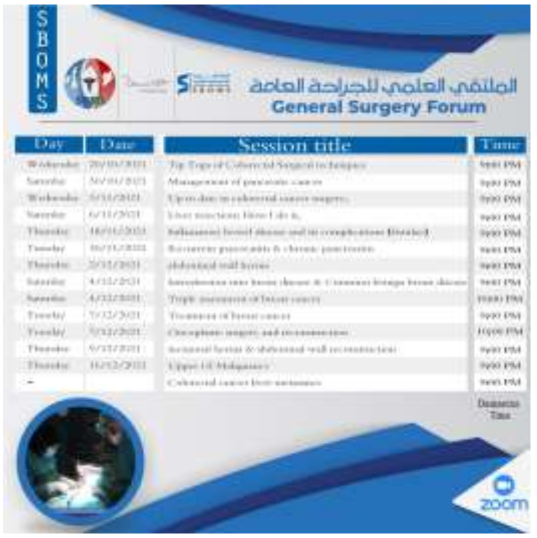	6th February 2022 at 1800 UK time. Aimed at Syrian doctors new to the UK Focus for this seminar is on Medical Portfolios - overview of audit/ QI/ research/ management/ leadership etc<br>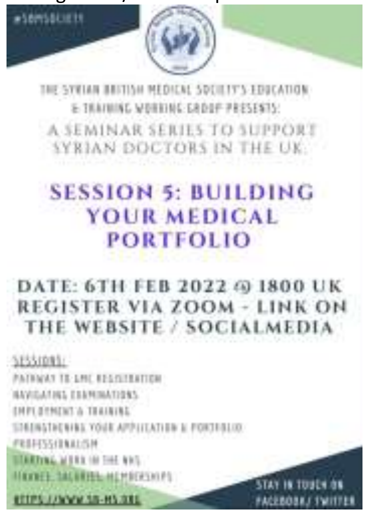<br>9th January 2022<br>We will focus on types of jobs and where to find them. We'll hear from speakers who have successfully navigated the system.Pathway to GMC registration<br>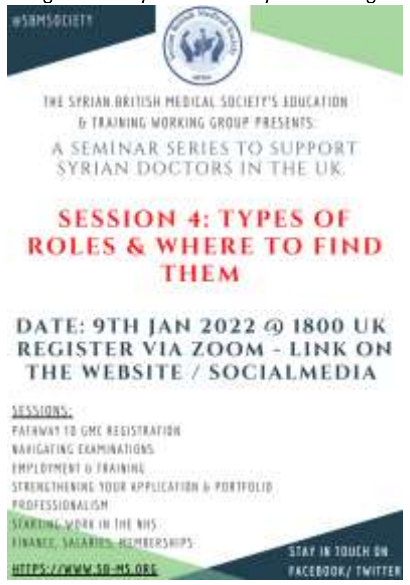<br>12th December 2021<br>SBMS Seminar Series for new doctors in the UK

					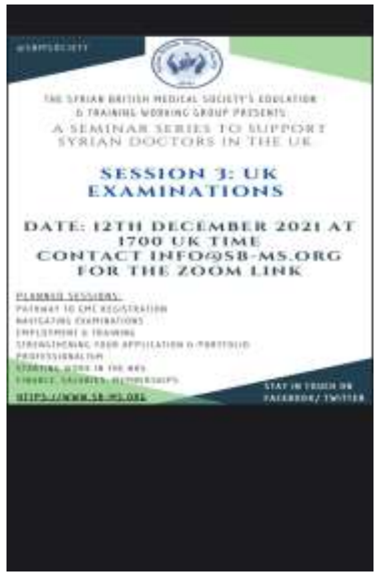<br><br><br>23rd November 2021<br>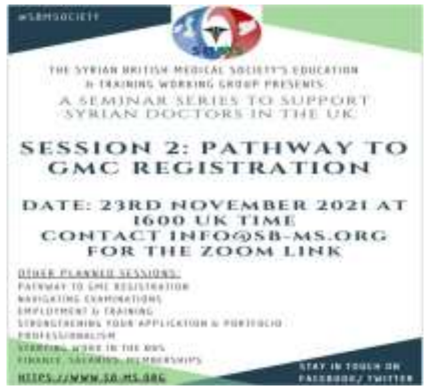<br><br><br>!SUMMARY<br>In October 2021, SBMS launched its seminar series for Syrian doctors who are new to the UK system. Our aim is to provide support, information, networking opportunities and mentorship through the process of examinations (language and medical,) GMC registration and clinical experience.<br><br>Seminar 1 (24th October 2021): Understanding the NHS and How it Functions<br><br>Seminar 2 (23rd November 2021): Pathway to GMC Registration<br><br>Seminar 3 (12th December 2021): UK Examinations (Language Examinations, MRCP, MRCS, MRCPCH etc)<br><br>Seminar 4 (9th January 2022): Types of Roles and Where to Find them<br><br>Video links are found here: Part 1 (Dr Firas Aljanadi) Part 2 (Dr Alaa Haafar) Part 3 (Dr Mohamed Kajouj)<br><br>Seminar 5 (6th February 2022): Building your Medical Portfolio (audits, QI, research, management, leadership etc) Video Links are found here: Part 1 (Dr Firas Aljanadi)

					and Part 2 (Dr Aula Abbara)<br><br>Seminar 6 (18th September 2022): Revalidation and Appraisals for doctors with Dr Atef Hakmi and Dr Abdallah Mawas. Video link is found here<br><br>Seminar 7 (23rd October 2022): Communication, Professionalism and Team work with Dr Abdallah Mawas.
Tanzania UK Healthcare Diaspora Association	http://tuheda.org	TUHEDA was established in order to facilitate the relief of sickness and preservation of health of the people in Tanzania and United Kingdom through:<br>Collaboration in healthcare provisions between UK and Tanzania<br>Providing or assisting in the provision of medical equipment in Tanzania<br>Organising seminars, conferences and training for healthcare professional and the public<br>In addition to all that, we will encourage and support our community and healthcare colleagues in Tanzania to build their knowledge and skills through organised visits or online discussions.	NA	NA	NA
Thai Physicians Association of America	https://tpaa.us	Thai Physicians Association of America is a national organization comprised of over 1,000 Thai doctors dedicated to:<br>Promoting the advancement of medical and scientific knowledge.<br>Maintaining the highest possible medical standards and ethics.<br>Conducting activities directed towards the sustenance and advancement of health care delivery and education in Thailand and the United States.<br>Establishing and promoting cooperation with related organizations in Thailand, the United States, and other countries.<br>Maintaining close association and fraternity among members.	NA	NA	NA
Ukrainian Medical Association of North America	https://www.umana.org	The Ukrainian Medical Association of North America (UMANA), founded in 1950, is a voluntary non-profit association of professionals licensed to practice in their areas of health care in the United States and	NA	20th February 2022<br>Second Scientific Virtual Series Sunday February 20th.	January 23rd 2022<br>Join us for the first of UMANA Virtual Scientific Conference Series with Dr. Luke Tomycz and Maria Soroka (razomforukraine.org)this coming<br>Sunday, January 23rd at 1PM EST<br>discussing the Co-Pilot Project!

		Canada.<br><br>UMANA's objectives are:<br><br>-Unite health care professionals of Ukrainian descent, who share an interest in promoting the health of Ukrainians<br>-Share medical knowledge and scientific research with emphasis on aspects unique to Ukrainians		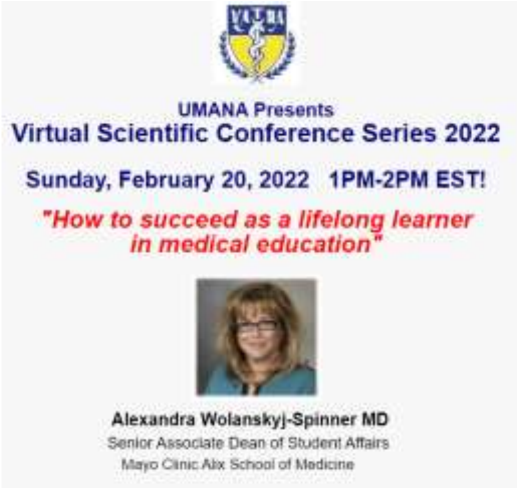<br>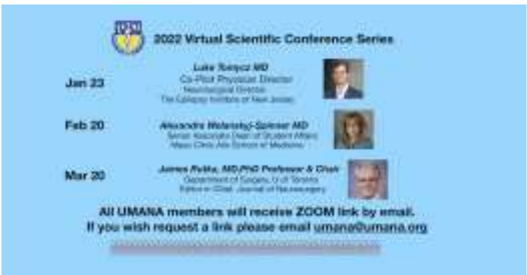	The Co-Pilot Project (CPP) is an initiative that aims to address the significant deficit in high-quality neurosurgical and spine surgery<br><br>training in Ukraine. Just as a co-pilot acts as another set of eyes and ears for the pilot, the mission of the program is to send surgeons from North America to mentor and aid Ukrainian surgeons through difficult cases.<br>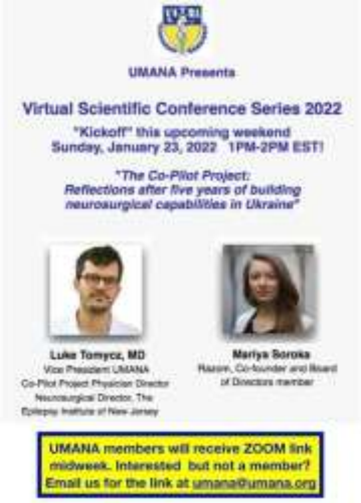
Ukrainian Medical Association of the UK	Added : https://www.umauk.org/<br><br>http://www.umauk.com	Aims:<br>To provide a forum to promote the fellowship of Ukrainian healthcare professionals through social, educational and cultural events.<br><br>To raise awareness of Ukraine, and its healthcare system, within the professional healthcare organisations of the UK.<br><br>To provide help and support to members and non-members in establishing and furthering their careers within the UK healthcare system.<br><br>To establish links with and support organisations (charitable and otherwise) as well as individuals in the UK and elsewhere associated with improving healthcare in Ukraine.<br><br>To develop ties with academic and professional healthcare organisations in the UK and Ukraine to promote social, cultural, educational and research activities.<br><br>To promote the Association	June 7th 2022<br>9:30-19:00<br>13th extraordinary conference<br>(containing medical education for ukranian medical students)<br>The meeting is aimed at Ukrainian medics and those who help Ukraine<br>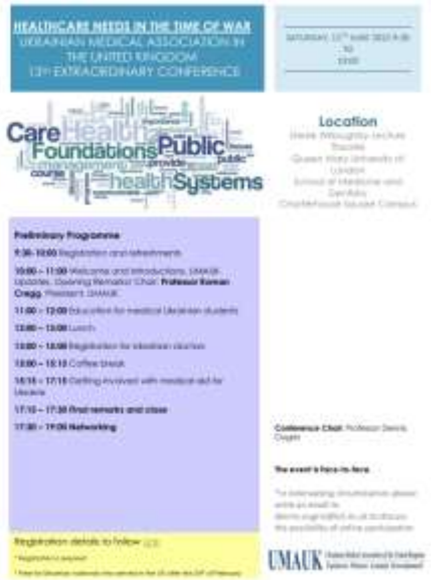	NA	NA

		amongst the Ukrainian community in the UK as a resource to improve access to healthcare advice and information.			
United States Colombian Medical Association	https://uscma.wordpress.com	NA<br>Website found but not 100% in English	NA	NA	NA
University of Santo Tomas Medical Alumni Association in America (Philippine)	https://ustmaaamerica.wildapricot.org	It was founded in 1987 by a group of pioneering Thomasian physicians as an umbrella organization that coordinates the different regional organizations in the United States and form one common body that will provide communications, channel support and coordinate activities with its alma mater, the University of Santo Tomas, especially the Faculty of Medicine and Surgery.	NA	June 22nd 2023<br>30th USTMAAA Grand Reunion and Medical Convention with CME morning sessions<br>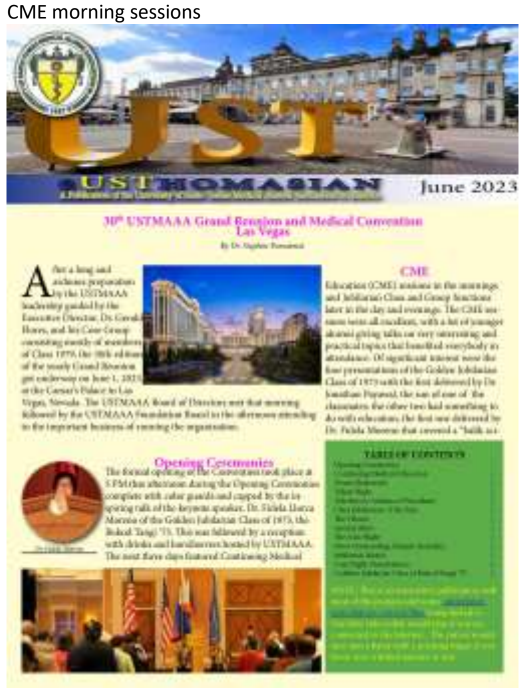<br>July 1-3 2022<br>29th USTMAAA GRAND REUNION AND MEDICAL CONVENTION<br>CME PROGRAM: "Latest Update in Medicine and Surgery"<br>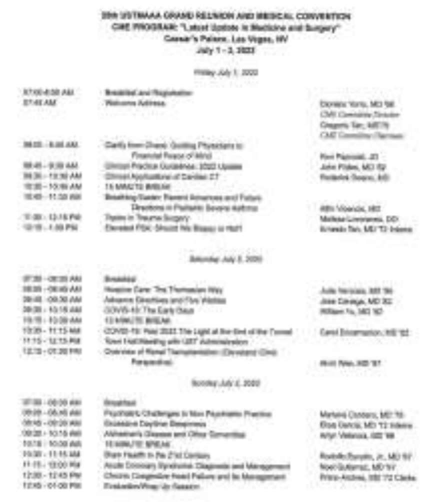<br>29th USTMAAA GRAND REUNION AND MEDICAL CONVENTION<br>CME PROGRAM: "Latest Update in Medicine and Surgery"<br>Caesar's Palace, Las Vegas, NV<br>July 1 - 3, 2022	NA
Venezuelan	https://www.vamainfo.com	Since 1996 we are a non for profit	Website found but programme details not in English	Website found but programme details not in English	Website found but programme details not in English

American Medical Association		organization of Venezuelan physicians residents in the US.<br>We will work to achieve our vision by offering:<br>1)Expert professional guidance to our members, whenever they need it.<br>2)A network where they can connect with each other, through our social media and events.<br>3)Preparation of our doctors to respond to natural disasters or any emergent health crisis scenario in Venezuela.			
Vietnamese American Medical Association	http://www.vamausa.org	The Vietnamese American Medical Association (VAMA) was established in1987 as a non-profit organization for physicians of Vietnamese heritage who live and practice in the United States.The VAMA establishes a venue for members to participate in continuing education programs related to public and private health care issues, diagnoses and treatments of diseases, and community outreach programs.	NA	NA	NA
Vietnamese Physicians Association	Although this representative organization has no website, there are a number of its chapters in each state.<br><br>Added for Northern Carlifonia http://www.vpanc.com/ :	Since its inception in 1989, VPANC has been hosting educational events for physicians and allied medical professionals in the community. Association's primary goals are to promote friendship and professionalism among its members; and to contribute to the development of the Vietnamese American community through professional activities.	NA	NA	NA
